# Political engagement and online political hostility among American X/Twitter users

**DOI:** 10.1371/journal.pone.0360061

**Published:** 2026-11-11

**Authors:** Stig Hebbelstrup Rye Rasmussen, Mathias Osmundsen, Michael Bang Petersen

**Affiliations:** Department of Political Science, Aarhus University, Aarhus, Denmark; Leiden University: Universiteit Leiden, NETHERLANDS, KINGDOM OF THE

## Abstract

Hostility and toxicity permeate political debates on social media, but less is known about which users engage in hostile political expression online. While many early theories of political behavior highlighted the positive democratic role of political engagement, recent research suggests that such engagement may also be associated with more polarized and antagonistic behavior. Combining two years of survey and behavioral data from X/Twitter, we examine associations between online political hostility and indicators of political engagement and its antecedents among American users. Online political hostility is positively associated with these indicators, with the strongest associations for political interest, political knowledge, and online participation. These associations are more pronounced for some indicators, especially online and offline participation, among users with more polarized affect directed towards US political leaders. Finally, the results show that engaged but hostile social media users do not simply cater to fringe audiences; they post more frequently and have more friends and followers. The greater engagement of hostile social media users may thus provide them with a higher reacher than other users.

## Introduction

No more than two decades ago, political debate took place in closed social circles, town hall meetings, and the opinion pages of newspapers. Today, the debate has moved to the public network societies of online social media [1]. This radical transformation of citizens’ political participation was believed to hold enormous potential for the quality and scope of dialogue as it democratized access to the public sphere [2]. Even without formal connections or the ability to write in prose that satisfied the editor of a newspaper, everyone now had a voice and could engage with others online, including politicians. Yet lowering the entry barriers to the public sphere seemingly came with a cost. As any regular user of social media will know, many online political conversations are anything but friendly. Citizens view online political conversations as far more negative and hostile than offline political conversations [3]. Even more concerning, hate speech directed at minority groups, incitements of violence against political enemies, and other uses of hateful and toxic language are commonplace on online platforms [4–7]. Although the proportion of hateful posts out of the total number is small according to some studies [8,9], this does not imply they are not influential in social media networks [10,11], or perceived as problematic [12].

Existing explanations of online hostility can be grouped into at least two distinct literatures. One set of accounts emphasizes socioeconomic marginalization, status threat, and grievance. From this perspective, hostility may be connected to experiences of deprivation, exclusion, or perceived loss of social standing among certain segments of the population (e.g., rural voters with low incomes and no college degrees) [13–15]. A second set of accounts emphasizes personality and emotion-regulation. Psychological research on online aggression, cyberbullying, and trolling often points to antagonistic traits, sadism, or situational triggers of anger and frustration [16].

In this article, we examine a different possibility. Classical research on political behavior emphasizes that education, internal efficacy, political interest, political knowledge, and participation are central to democratic engagement [17–19]. These factors are often normatively valued because they help citizens follow politics and take part in democratic life. Yet even classical theories recognized that engagement could become excessive [20], and more recent work on motivated reasoning and political polarization suggests that politically engaged citizens may also be more likely to process information in biased or antagonistic ways [21]. We extend this revisionist perspective to online political hostility by asking whether hostile political expression on X/Twitter is associated not only with deprivation or personality-based explanations, but also with political engagement and its antecedents.

Empirically, we rely on a dataset of behavioral data on online political hostility from the social media platform X (named Twitter at the time of the data collection). We combine these data with panel survey data on political engagement among American users. On this basis, we provide three sets of analyses. First, using cross-sectional and panel analyses, we estimate associations between online political hostility and several theoretically distinct indicators: distal antecedents of engagement (i.e., education and internal efficacy), motivational and cognitive indicators of engagement (i.e., political interest and political knowledge) and direct measures of participatory engagement (i.e., online and offline political participation). Second, we provide suggestive analyses of whether these associations differ by affective polarization [22], measured as polarized affect towards political leaders. Third, we examine whether politically hostile users are more active and have greater potential reach on X/Twitter, while avoiding claims about formal network influence or centrality.

## Online political hostility: Definition and existing research

We define *online political hostility* as the expression of degrading, malicious, and intimidating online behavior towards individuals or collectives based on political differences. Online political hostility shares commonalities with other forms of online extremism, like hate speech [6] and cyberbullying [23], that have increasingly come under scrutiny by researchers across different scientific disciplines. It involves hostile behaviors expressed through various means – comments on visual or auditory content, memes, posts and shared posts, and postings on social media platforms – that transgress the boundaries of what ordinary citizens consider acceptable input into political conversations. Of critical concern, the key distinguishing feature of online political hostility is the explicit focus on hostile content that enters the realm of *politics*. Accordingly, toxic online exchanges in the aftermath of, say, a sports game fall outside the purview of what we examine here.

As conversations about politics and other social issues rapidly have moved into the online world, studies show that online political hostility is widespread [3,15,24] and research teams across the globe are focusing on developing counter-strategies that can shield against the detrimental effects of hostility. As a consequence of these developments, our knowledge of the dynamics of online hostility is constantly expanding. We now know that social media users typically become *victims* of online hostility based on a host of personal attributes, like gender, social class, ethnicity, or sexual orientation [25–27]. We also know that people who are visible on social media [28,29] and who confront the hostile behavior of others [28,30–32] often fall victim to online hostility themselves.

At the same time, we know less about the characteristics of the *users who express* online hostility; that is, the set of features that may be associated with social media users expressing hostility towards others in online political discussions [3,33]. As [6, p. 60] succinctly summarizes: “Less is known about the actors in informal communities dedicated to producing harmful content, or the accounts that produce hate speech on mainstream platforms.”

Existing work has emphasized at least two different explanations for hostile online behavior. Psychological accounts focus on personality and emotion-regulation, including antagonistic traits, sadism, negative mood, and situational triggers of anger or frustration. A central finding in the psychological literature on cyberbullying and so-called trolling is that the most severe cases of hate can be attributed to a (relatively) small segment of social media users with extreme personality traits, like those scoring high on the “Dark Triad” [34] and sadism [35], which are intimately coupled with a lack of emotion regulation, while other research highlights how challenges of emotion regulation imply that “anyone can become a troll” [16,29,32]. Socioeconomic and grievance-based accounts instead focus on marginalization, deprivation, status threat, and perceptions of being left behind, especially among demographic groups – such as rural, white males with low incomes and little formal education – who perceive themselves to be economically and socially threatened by immigration, globalization, and new social norms [13–15,36,37].

The present study does not seek to adjudicate between these literatures. Instead, it asks whether online political hostility is also associated with political engagement and its antecedents. This focus is broader than studies of cyberbullying or trolling that are not necessarily political, and it is also broader than studies focused on specific forms of hate speech directed at marginalized groups [6]. We examine hostile expression in political exchanges on X/Twitter and ask whether such expression is associated with the same forms of engagement that are typically viewed as central to democratic citizenship.

### Political engagement, its antecedents, and online political hostility

While research on online hostility is in its infancy, there is a solid literature about the sources and conceptualization of political engagement [17,18]. Political engagement, typically conceptualized as the motivation to become and stay informed about political affairs and pursue one’s self-interests through political participation [17,18], is a cornerstone of the democratic process [18].

One classical answer is that civic engagement rests on two key building blocks: the *motivation* and the *capacity* to engage in politics [18,19]. Two of the most studied, and important, constructs affecting the motivation and capacity to participate in politics are (1) formal education and (2) internal political efficacy. Traditionally, education has been linked to a host of “participation-enhancing skills such as cognitive ability, civic skills, and civic orientations” [38, p. 613]. It has been termed the “universal solvent” [39] for its ability to influence most constructs deemed important for citizens’ political activities, such as the motivation to become politically interested, and cognitive factors such as knowledge of politics and cognitive sophistication (cf. also [17,40,41]). And although it seems that the effect size of education on political engagement was likely overstated in early studies [38,42,43], its continued research focus testifies to its ongoing importance. In a similar vein, internal political efficacy is one of the strongest predictors of political engagement and one of the most studied constructs in the literature on political engagement [44]. A particularly important form of efficacy is *internal efficacy*, which is the subjective feeling of competence in politics [45,46].

Within the classical literature, political engagement has been seen as a boon to democracy. Formal education and a strong sense of internal efficacy potentially equip citizens with the motivation and capacity to partake in respectful conversations about topics of political importance online. At the same time, even in this classical literature, it was explicitly acknowledged that political engagement could go too far. In particular [20], argued that engagement needed to be tempered with a more parochial relationship to politics such that “the democratic citizen is called on to pursue contradictory goals: he must be active, yet passive; involved, yet not too involved; influential, yet deferential.” ([20], p. 478–479). Recent research has more strongly emphasized this revisionist view on the costs and benefits of political engagement. Most clearly, the literature on political information processing has argued and found that indicators of political engagement (e.g., partisanship) can be associated with biased assessments of political arguments and news such that arguments and news that are aligned with an individual’s political opinions are evaluated less critically by the engaged than arguments and news that are not aligned [21,47]. Importantly, this literature also provided evidence that biased information processing is further exacerbated among those high in political knowledge [48,49].

The research focus on the potential negative sides of political engagement has increased as many Western democracies are affected by processes of political dissatisfaction [50] and political polarization [22]. This includes the United States, the site of the present study [22]. In such settings, politically engaged citizens may use their political competences and motivation not to make political exchanges less but more contentious. Accordingly, political engagement and its antecedents may be associated with rather than diminish online political hostility. Currently, there is already some evidence that dissatisfaction is associated with increased political engagement, as those who are dissatisfied want to make their voices and preferences heard [51]. Similarly, increasing levels of affective polarization have been found to be associated with increased political participation [52]. This potential negative side of political engagement is the key focus of our empirical investigation. Importantly, prior research has only to a limited extent applied this revisionist perspective om political engagement to politics on social media and mainly in the context of misinformation sharing [53]. Here, we extend and integrate the study of the negative side of political engagement into the study of online politics with a specific focus on online political hostility.

When investigating the correlates of political engagement it is important to note that the existing literature, as is clear from the above discussion, utilizes a number of different indicators of political engagement. Education and internal efficacy are distal antecedents that may facilitate engagement by increasing civic skills, confidence, and perceived competence. Less distal measures include political interest and political knowledge that are better conceptualized as motivational and cognitive indicators of engagement [17,40]. Online and offline participation are more direct indicators of engagement. While some studies investigate the relationships between these different types of indicators and measures [18,19], we will in the present study treat them as separate indicators as they may not operate through the same pathways.

### Affective polarization as a conditional context

As noted above, political polarization may shape whether political engagement translates into hostility. In research, polarization is often measured as affective polarization, i.e., the tendency to view one’s in-group favorably and out-groups unfavorably [22,54]), and it has previously been found to make both the political system and people’s everyday lives less “consensual.” For example, prior research shows that economic pricing decisions can be affected by whether the customer is an in-group or out-group partisan [55], that affective polarization can negatively affect political trust over time [56], and that out-party animus can even lead to a reluctance to follow life-saving health guidelines during a global pandemic [57].

Affective polarization may condition the association between political engagement and online political hostility because it shapes the emotional orientation of political involvement. Engagement provides attention, motivation, skills, and opportunities to participate in political discussion. Affective polarization may shape whether such participation is expressed in antagonistic terms toward political opponents. Under this logic, the association between engagement and hostility should be stronger among users who evaluate opposing political elites more negatively.

## Empirical expectations

On the basis of the literature and arguments reviewed above, we derive three expectations.

**Expectation 1.** Indicators of political engagement and its antecedents are positively associated with online political hostility.

As is clear from existing research, political engagement is reflected in many different indicators and not all indicators have the same theoretical status. Education and internal efficacy are best understood as distal antecedents of engagement: they may provide citizens with skills, confidence, and perceived competence in politics. Political interest and political knowledge are more proximate motivational and cognitive indicators of engagement. Online and offline participation are more direct expressions of engagement. These factors may all be associated with online political hostility, but potentially for different reasons. Expectation 1 reflects that citizens who are motivated, informed, and accustomed to political participation may be more likely to enter political exchanges on social media, including hostile exchanges. At the same time, we will make no attempt to adjudicate among alternative causal models regarding the relationships between these different indicators and measures.

**Expectation 2.** The associations between online political hostility and indicators of political engagement and its antecedents are stronger among users with higher levels of affective polarization.

This conditional argument is not a claim of causal identification. Affective polarization may itself increase political interest or participation; political sophistication may increase both affective polarization and hostility; and hostile online interactions may increase affective polarization over time. We therefore interpret the moderation analyses as descriptive evidence about conditional associations rather than as evidence that affective polarization causally moderates the effect of engagement on hostility.

A final question is whether the potential engagement-hostility nexus matters for overall conversations? An increasing concern is that social media discussions are shaped by the most hostile and extreme voices [3,58] and the role of engagement for hostility may help explain this. To investigate the implications of the other findings of the study, we therefore develop a final expectation:

**Expectation 3.** Online political hostility is associated with greater activity and potential reach on X/Twitter, measured by posting frequency and numbers of friends and followers.

We treat this final expectation as exploratory in nature given the lack of existing research.

## Materials and methods

### Ethics statement

This study was conducted in accordance with the Code of Conduct for Research Integrity of Aarhus University and associated policies of the National Committee for Health Research Ethics. The study was exempt from full ethical review under Danish regulations governing research using publicly available social media data and survey research with adult participants providing informed consent.

All participants were recruited through YouGov’s standing web panel in the United States. Participation was voluntary, and participants provided informed digital consent at multiple stages. Before participating in any survey, potential respondents were informed about: (1) the content and goals of the research project, (2) the identity and contact information of the research team, (3) their right to withdraw consent at any time, (4) the voluntary nature of participation, and (5) the option to decline participation.

At the time of the study, the focal social media platform was named Twitter but has since changed name to X. In the rest of the paper, we will use the new name, X. Participants were specifically asked if they wished to share their X handle to link their publicly available posts to their survey responses. Before providing their X handle, participants received a detailed description of how their X data would be used, including assurances that no data would be presented outside the research project in any form that would make them personally identifiable. Only publicly available posts were collected, in compliance with X’s Terms of Service at the time of data collection.

All participants were adults (18 years or older); no minors were included. No waivers of consent were granted. Participants received 500 YouGov points (approximately $6 USD) for completing the 15-minute surveys, consistent with standard YouGov compensation rates.

The data were processed in accordance with the General Data Protection Regulation (GDPR) under the oversight of the Aarhus University Data Protection Unit.

### Data

We rely on a data infrastructure combining panel survey data with online behavioral data from X (see also [59]). To obtain our data, we commissioned the survey agency YouGov to invite panelists from their standing web panel to participate in a series of panel surveys. We recruited the original set of panelists in January 2019. Here, participants were asked to complete a survey with measures of our set of potential antecedents of online political hostility and, importantly, to provide their X ID and give consent to let us scrape their publicly available X data and link it to their survey answers. These participants, along with an additional set of panelists who were included to increase the study’s power in September 2020, were then asked to participate in five additional survey waves, administered at various time points until the final survey in January 2021. The total number of panelists where we were able to link survey responses to their X handle in this period was 2567. For the analyses where we only use X data the maximum number of respondents is 3643. Full number of respondents can be seen in tables in the main text or the appendix. The six surveys did not include the same questions, and, consequently, not all measures described below are available in each wave. Further, as participants were free to opt in and out of the different surveys, the final sample size for each wave differs. Tables in S1 Appendix outline in greater detail which measures and how many participants were available for each wave.

The data collection focused on the United States specifically because the associations of interest may be especially visible in highly polarized political environments. The United States provided an ideal context given its well-documented high levels of affective polarization and political distrust during the study period.

The scraping of X commenced on March 4, 2019 and stopped in January 2021. During this period, we managed to scrape 9,250,180 posts from our panelists. All measures based on X data are aggregated at the level of the individual panelist since our unit of analysis is individual panelists and not posts.

Data and scripts to reproduce the main analyses can be found on OSF.

### Measures

#### Online political hostility (primary measure).

Our key measure of online political hostility is based on a word embeddings approach termed the Super-Unsupervised (SU) approach [60]. The gist of the method is that through their use of language in posts, respondents *themselves* define what constitutes the linguistic space of political hostility. This method has been validated in [60], where its face validity, convergent and discriminant validity, criterion validity, external validity and ecological validity were established.

The method consists of four steps. First, we create *word vectors* based on posts from our panelists using the word2vec method [61,62]. Word vectors are numerical representations of a word in a multidimensional space. Word vectors can therefore be used to measure the distance between words in semantic space. Second, we combine the word vectors “political” and “hate” into a combined vector that captures the co-occurrence of political and hostile language in the corpus. Third, we use this combined measure to calculate the cosine similarity between individual posts and the “political hate” word vector: If a post contains many words that are close to the “political hate” word vector, it receives a higher hostility score; if the post is further away from the word vector, it receives a lower hostility score. Fourth and finally, we create a measure of a respondent’s general level of political hostility by averaging the hostility scores of all their posts; for the panel models we compute this average per time unit investigated (wave or month, depending on the analysis). This measure of overall political hostility constitutes our primary dependent variable. We prefer to use the term “political hostility” rather than “political hate” since we do not wish to confuse this more general measure of online political hostility with the narrower concept of “hate speech” which we are not purporting to measure.

We conducted (and replicate here in summary) five complementary validity assessments. Full methodological detail and diagnostics are available in the prior methods paper.

##### Face validity of the overall measure.

To assess the face validity of the overall measure [60], inspected the posts with the highest and lowest political hostility scores. The posts with the highest scores clearly contained hostile language directed at political out-groups (e.g., derogatory characterizations of political opponents, calls for violence against political groups, and moralized criticism). In contrast, the posts with the lowest scores were either neutral or positive in tone and did not contain any hostile language. This pattern indicates that the overall measure captures posts that are genuinely hostile in political contexts, thereby establishing its face validity. They found that posts with high Perspective API scores concentrate on profanity and insults, whereas high political hostility scores combine political referents with hostility (e.g., elites/parties plus derogation), matching the construct.

##### Convergent and discriminant validity.

The SU approach compared this score to other available text-based indicators in our data: a lexicon-based sentiment score (AFINN), Google Perspective API toxicity, and a supervised classifier trained directly on the same expert labels. We use this validated score as our main indicator of political hostility in the analyses below. We know from [60] that the SU score correlates more strongly with, e.g., political interest (0.43) and political knowledge (0.44) than both AFINN (−0.28 and −0.29) and Perspective API toxicity scores (0.24 and 0.22), suggesting that the SU score captures politically relevant hostility better than these more general hostility measures.

At the same time convergent validity was established through correlations with related measures: Google’s Perspective API toxicity scores (r = 0.59), and the AFINN sentiment lexicon (r = −0.62, negative because AFINN codes negativity as negative values) [60]. The correlation with Perspective API demonstrates that our measure captures hostility in a manner consistent with established toxicity measures, while the moderate correlation magnitude reflects that our measure specifically targets political hostility rather than general toxicity across all contexts. A full correlation matrix from the data used in this paper can be found in S1 Appendix.

The SU approach was also validated against 2,469 hand-labeled posts rated by four expert raters using the definition of “Political Hostility” as: “Degrading, harassing and intimidating online content grounded in political differences.” [60]. The expert-coded set was approximately balanced across the two classes, so a naive baseline (always predicting the majority class) would achieve about 0.50 accuracy. We trained the score on a training split of these labelled posts and evaluated it on a held-out test split from the same domain (i.e., panelist posts), thereby avoiding in-sample inflation.

Here, it reached 74% accuracy against a 50% baseline when dichotomized on a held-out test set. With “Political hostility” as the positive class, the approach demonstrated precision of 68.82% (when the classifier predicts a post contains political hostility, it is correct 68.82% of the time), recall of 77.57% (the classifier identifies 77.57% of all hostile political posts), and an F1 score of 0.7293, indicating strong balanced performance. The confusion matrix showed 854 true positives, 981 true negatives, 247 false positives, and 387 false negatives. We use the supervised classifier as an additional robustness check in S1 Appendix.

##### Criterion validity.

Among the 20,000 most politically hostile posts by SU, political actors and institutions are over-represented: e.g., “Trump” appears 4,163 times vs 831 among the 20,000 most toxic (Perspective) posts — consistent with SU identifying political hostility specifically. In addition applied to a prelabelled dataset of offensive and hateful posts [63], a simple percentile mapping of SU Hate yields balanced accuracy of.47 (chance = .33) and identifies the rare “hate speech” class 3× better than random — demonstrating criterion-linked ordering absent any supervision [60].

**External validity.** Two tests indicate portability beyond this corpus: After aligning with public TwitterGloVe embeddings, SU “political hate” scores correlate r = .83 with scores computed in this external space and using SU to score news-domain quality, SU distances from (nytimes+washingtonpost) correlate .84 with expert fact-checker ratings at the domain level and .90 at the user-average level — illustrating transfer to a different task and unit of analysis.

##### Ecological validity.

Democrats’ “political hate” space is relatively closer to “fearmongering/right-wing/Trumpian,” whereas Republicans’ is closer to “left/Democrat,” matching well-known partisan schemata. Dependency parsing of the top 100k hostile posts shows the out-party as the grammatical actor in the large majority of cases (e.g., for Democrats: GOP/Republicans/Trump are subjects in 51–78% of top posts; for Republicans: Democrats/Dems/Obama in 75–87%).

And in Denmark, Sweden, Germany, and Italy, national Political Hostility aggregates rise toward election day and peak exactly on that day, then decline, as expected if the measure tracks political conflict salience.

#### Robustness measures of online hostility.

The above validation exercises support the use of the SU score as our primary measure, but they do not eliminate all construct-validity concerns. Because the measure is based on proximity to an embedding space built from political and hostile terms, it may partly capture proximity to politically salient conflict language or hate-related political vocabulary rather than hostility alone. We therefore interpret the SU score as an aggregate indicator of online political hostility and assess the robustness of the findings using additional measures. Specifically, to assess the robustness and generality of the findings, we also used two additional measures of online hostility or negativity.

First, we use a classical dictionary-based sentiment approach, the AFINN dictionary [64], which assigns sentiment scores ranging from −5 (very negative) to 5 (very positive) to individual words. While the AFINN dictionary is a standard tool for measuring sentiment [65], it has known limitations on social media text and should be viewed as a secondary robustness measure rather than our primary operationalization of political hostility. The correlation between AFINN scores and our embedding-based measure is r = −0.62 (negative because AFINN codes negativity as negative values), indicating moderate alignment while also highlighting the differences between general sentiment and political hostility specifically.

Second, we use Google’s Perspective API [66] to detect online toxicity. The Perspective API was developed using Wikipedia’s editorial discussions and, despite some criticisms [67,68], represents one of the most prominent approaches for measuring online toxicity. The correlation between Perspective API toxicity scores and our embedding-based measure is r = 0.59, demonstrating substantial convergent validity. We use the term “toxicity” specifically when referring to Perspective API scores to distinguish it from our primary measure of “political hostility.”

It is important to stress that although a general notion of online hostility works in some cases, in many situations a much greater conceptual and empirical delineation of types and targets of hate speech is called for [69]. We thus do not mean to imply that the ways of measuring online hostility presented here are the only ways to measure it.

#### Political engagement and antecedents.

From the surveys, we obtained individual-level measures of respondents’ education, sense of internal efficacy, political knowledge, political interest, self-reported participation in offline settings, and self-reported participation in online settings. We organize the explanatory variables into three conceptual groups. First, education and internal efficacy are distal antecedents of political engagement. Second, political interest and political knowledge are motivational and cognitive indicators of engagement. Third, online and offline political participation are behavioral indicators of engagement. This distinction follows the theoretical framework above and avoids treating all variables as interchangeable indicators of a single resource construct. Treating these variables separately is important because they need not operate through the same pathway and because a single undifferentiated scale may obscure meaningful theoretical differences. This decision to treat the indicators separately is also consistent with the analyses in S1 Appendix where a factor analysis does not support a one-factor measure of political engagement.

Descriptive statistics for all measures can be found in S1 Appendix. All details on the measurement and scaling of independent variables can also be found in S1 Appendix.

**Internal efficacy** is measured using the standard Center for Political Studies (CPS) questions [46]: “Sometimes politics seem so complicated that a person like me can’t really understand what’s going on” and “I feel that I have a pretty good understanding of the important political issues facing our country.” We also use a third item from the European Social Survey that asks whether “I often think it is difficult to make up my mind about political issues.” Cronbach’s alpha is 0.70 for our measure of internal efficacy.

**Education** is measured using six different educational categories: No HS, High school graduate, Some college, 2-year, 4-year, Post-grad.

**Political interest** is measured using a standard American National Election Studies (ANES) question asking respondents about their level of political interest on a five-point scale ranging from “Extremely interested” to “Not interested at all.”

**Political knowledge** is measured using five factual questions from the ANES, scored as the number of correct answers.

**Online and offline political participation** is measured as both online and offline political participation to fully capture the many ways citizens engage in politics. To measure traditional *offline* participation, we created an 11-item battery based on previous research [70,71], asking participants on scales ranging from “1 = Never” to “7 = Several times a day” how often they participate in eleven offline political activities, like “Contact a government official in person, by phone, or by letter,” “Work or volunteer for a political party, candidate or a social cause,” and “Wear clothes or use items in support of a political or social cause.” To measure *online* participation, we asked participants on the same response scale how often they engaged in ten online political activities [72,73], including “Comment on others’ online posts about a political or social cause (including online news articles),” “Like or follow a page of a political group, party or candidate on social media,” and “Start or join a political group or group supporting a cause on a social networking site.”

#### Affective polarization.

To investigate Expectation 2, we also included a measure of affective polarization. Specifically, the survey allows us to construct a measure of leader-directed affective polarization, i.e., a measure using feeling thermometer ratings of political leaders [22]. Respondents rated three Republican politicians (Ted Cruz, Donald Trump, and Mike Pence) and three Democratic politicians (Joe Biden, Elizabeth Warren, and Bernie Sanders) on a 0–100 feeling thermometer. We used respondents’ party identification to distinguish in-party from out-party leaders and calculated the difference between average in-party and out-party leader ratings. Higher values indicate more polarized feelings toward opposing party elites. The question asked was: “We’d like to get your feelings toward some of our political leaders and other people who are in the news these days on a’feeling thermometer.’ A rating of zero degrees means you feel as cold and negative as possible. A rating of 100 degrees means you feel as warm and positive as possible. You would rate the politician at 50 degrees if you don’t feel particularly positive or negative toward the politician.”

This measure captures affect toward party elites rather than affect toward parties as institutions or ordinary party supporters, as is standard in research on affective polarization. The survey did not include parallel feeling thermometer ratings of party supporters. We therefore interpret the moderation analyses as evidence about leader-directed affective polarization and discuss this measurement limitation below.

#### Control variables.

In assessing the empirical support for our expectations, it is key to account for alternative explanatory factors. We include the Big Five personality traits as broad control variables for stable individual differences in motivational, emotional, and cognitive dispositions [74–76]. These controls are not intended as a direct test of Dark Triad, sadism, or other antagonistic-trait accounts of online hostility. The survey did not include measures of those more specific traits, and we therefore cannot adjudicate those accounts directly. Instead, the Big Five controls help assess whether the associations between political engagement and online political hostility remain after accounting for broad personality differences. We rely on the Big Five Inventory (BFI) consisting of 44 items to measure the five major personality traits [76]. The alpha reliability was 0.82 for openness, 0.84 for conscientiousness, 0.87 for extroversion, 0.82 for agreeableness, and 0.87 for neuroticism.

In addition to personality measures, we also include a measure of party identification and standard demographic controls (age, gender, and income). Party identification is measured using a standard question from the American National Election Study on a seven-point scale ranging from “Strong Democrat” to “Strong Republican.” Standard demographics of age, gender and income were obtained from YouGov’s background information about its panelists.

### Analytical approach

As we discuss in more detail below, the different analyses rely on slightly different measures and approaches. Accordingly, for ease of exposition and coherence, we present the analyses’ methods individually before presenting the results.

Generally speaking, we use Mplus [77] for all statistical models and the statistical computing environment R [78] to connect to Mplus using the MplusAutomation package [79]. Most graphs use the plotting library ggplot2 [80], except the network graphs, where we use R’s built-in plotting functionality using the igraph package [81].

For the cross-sectional analyses, we use ordinary least squares (OLS) regression with cluster robust standard errors to account for the fact that a single X user can participate across multiple waves. This dataset is organized as a Time-Series Cross-Section (TSCS) dataset using pooled OLS. Not all measures are available in each survey wave and we therefore use multiple imputation and full information maximum likelihood to estimate missing values in these analyses. We elaborate on this in S1 Appendix.

For the panel analyses, we use Random-Intercept Cross-Lagged Panel Models (RI-CLPM). These models separate stable between-person differences from within-person change and therefore reduce concerns that the associations are entirely driven by stable time-invariant characteristics such as long-standing socioeconomic factors or broad personality traits. However, RI-CLPM estimates do not by themselves establish causal effects. Time-varying confounding, measurement error, and reciprocal relationships may remain. We therefore interpret the RI-CLPM results as temporal within-person associations that strengthen the descriptive evidence but do not provide causal identification. Details of the RI-CLPM approach are provided in S1 Appendix.

Because several models test related variants of the same general expectations, we also assess the robustness of key conclusions to multiplicity adjustments. We define four separate families of tests: the primary association models, the robustness outcome models, the affective-polarization interaction models, and the exploratory activity/reach models. Within each family we apply a Benjamini–Hochberg false-discovery-rate (FDR) correction and, for the more conservative reading requested by the reviewers, also report Bonferroni-adjusted 95% confidence intervals. The main figures report conventional 95% confidence intervals for readability, while the full multiplicity-adjusted results are reported in S1 Appendix. We interpret patterns that do not survive adjustment as exploratory.

All tables report 95% confidence intervals, and intervals that do not include zero are indicated with a *. Unless otherwise noted, independent variables are rescaled to range from zero to one, and the reported association estimates (Figs 1–4) are fully standardized (STDYX) coefficients, in which both predictors and outcomes are standardized. The exploratory activity and reach models are an exception: these are negative-binomial count models, and their estimates are reported on the original count scale.

**Fig 1:**
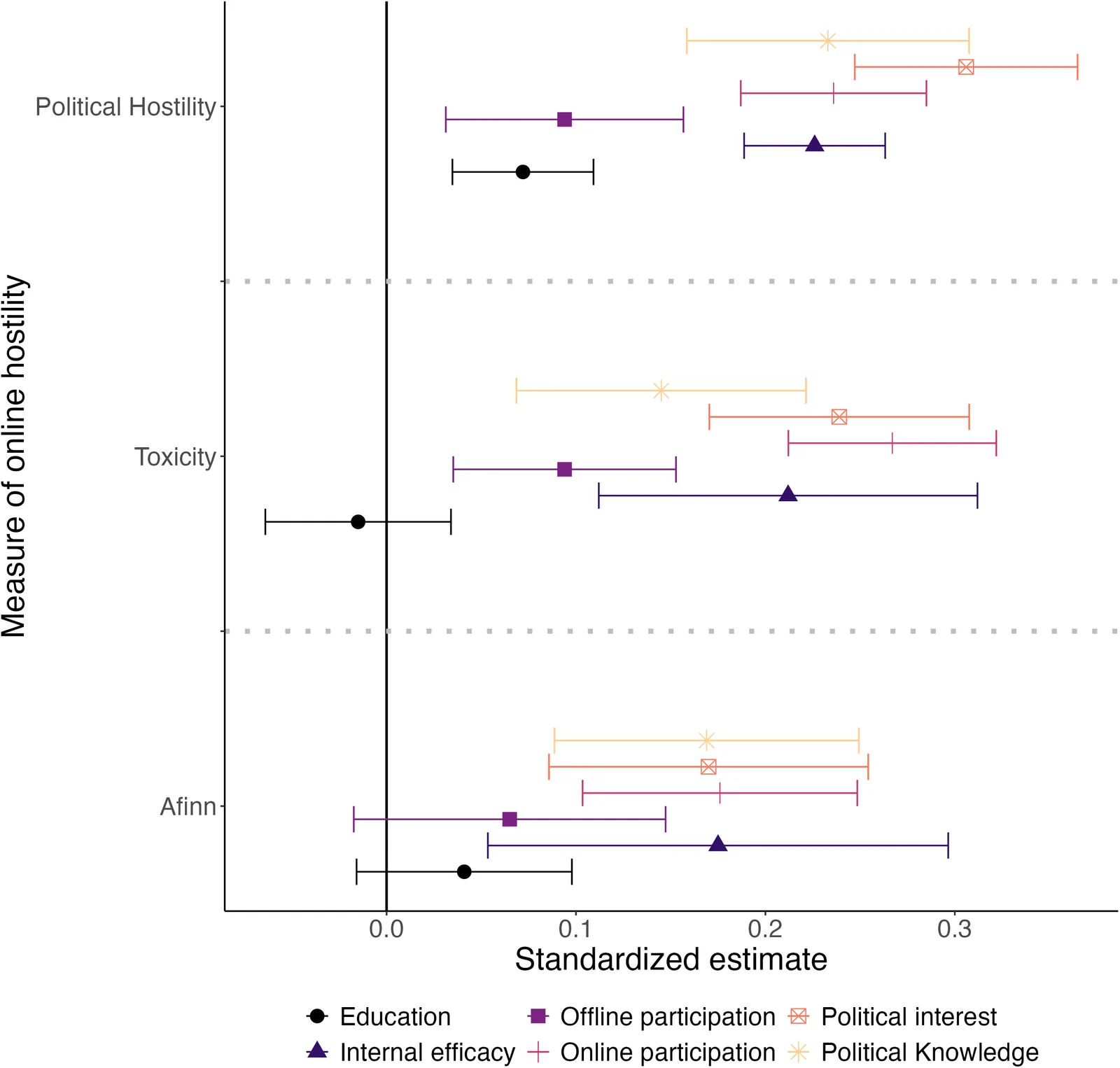
The association between indicators of political engagement, its antecedents, and online political hostility. All models control for the Big Five personality traits, income, age, gender, and party identification. The full results can be found in S1 Appendix. Error bars represent 95% confidence intervals.

**Fig 2:**
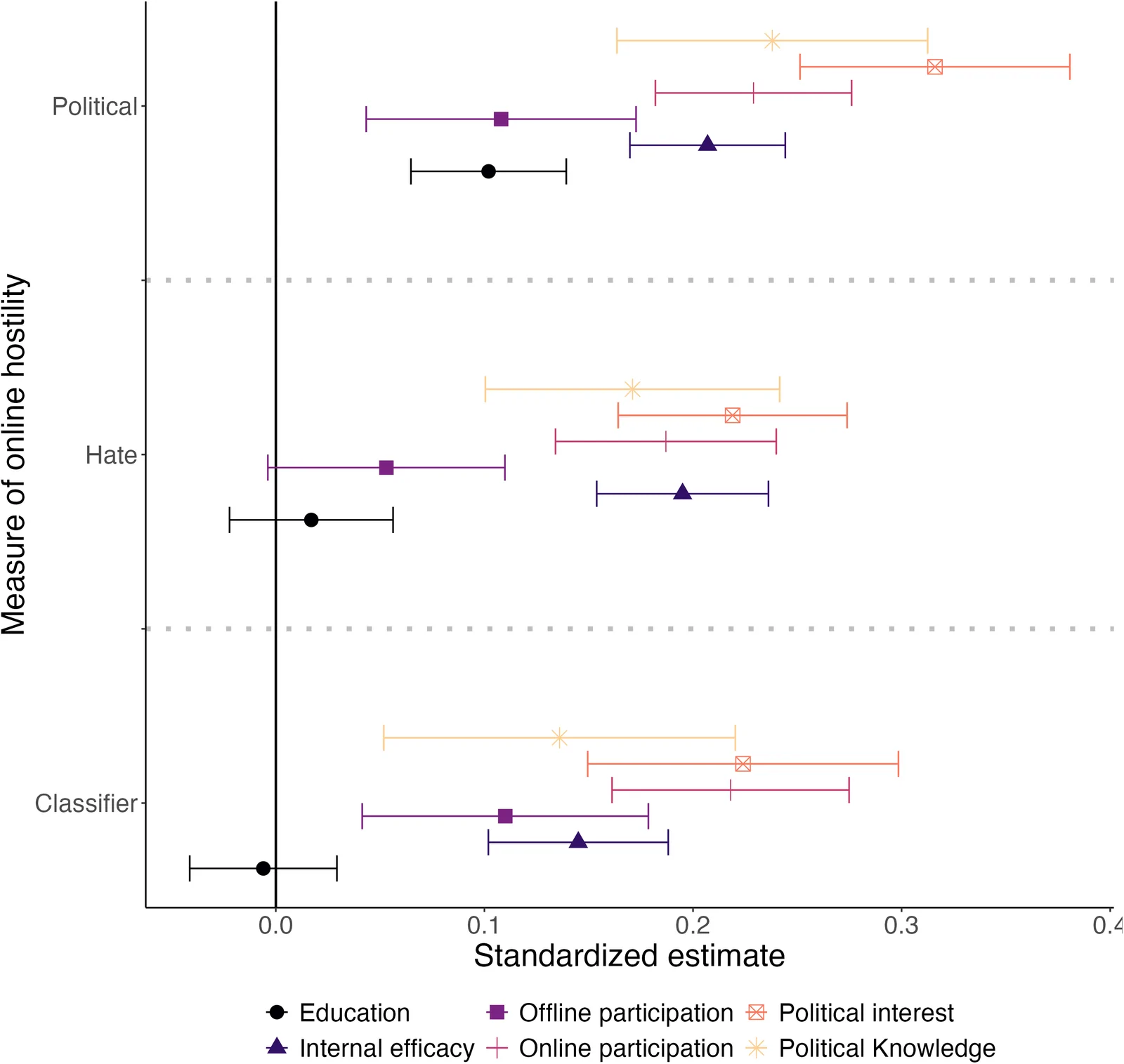
The association between indicators of political engagement, its antecedents, and components of the political hostility variable, “political” and “hate,” as well as an alternative classifier of political hostility. All models control for the Big Five personality traits, income, age, gender, and party identification.

**Fig 3:**
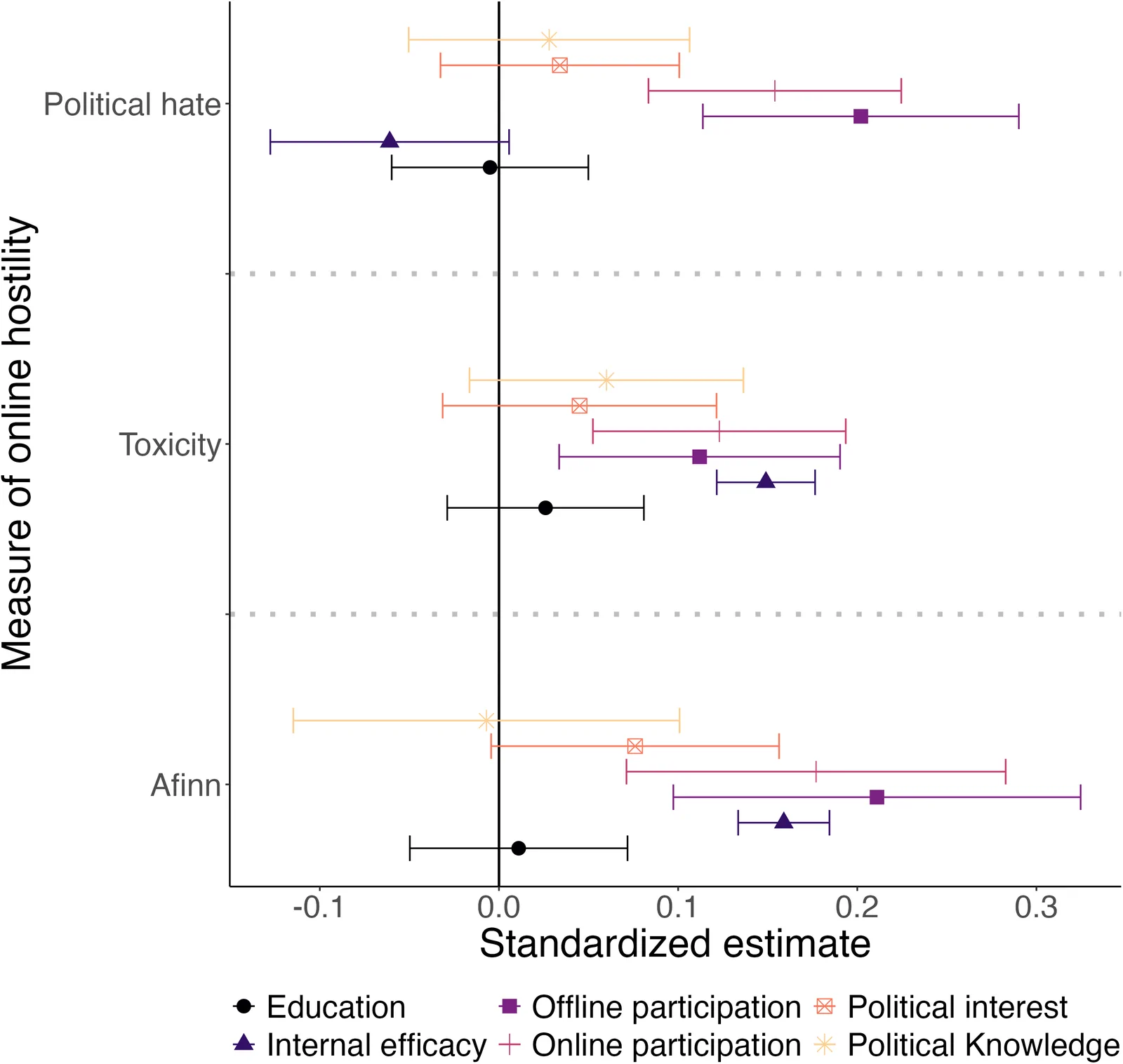
Formal interaction coefficients between indicators of political engagement, its antecedents, and leader-directed affective polarization. All models control for age and gender but not the full set of controls to reduce problems of multicollinearity. The figure illustrates the *interaction* term from each model from the interaction between the engagement indicators and leader-directed affective polarization. Full results can be found in S1 Appendix.

**Fig 4:**
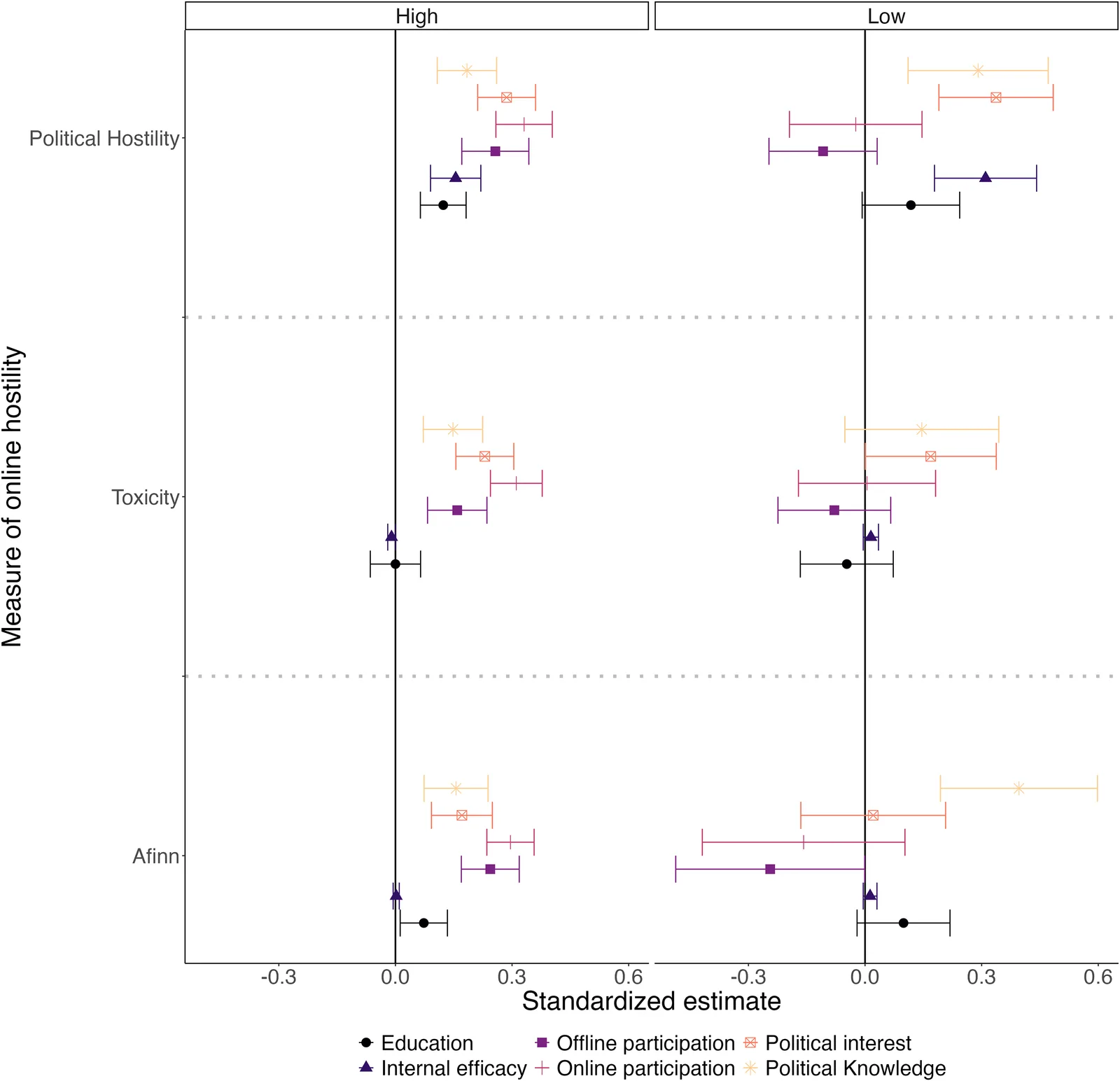
The association between indicators of political engagement, its antecedents, and online political hostility in groups high and low in leader-directed affective polarization. All models control for age and gender but not the full set of controls to reduce problems of multicollinearity. The figure illustrates the associations for those high and low on leader-directed affective polarization. Error bars represent 95% confidence intervals.

## Results

### Are political engagement and its antecedents associated with online political hostility?

To assess Expectation 1, we first present OLS regressions estimating associations between online political hostility and the three groups of indicators: distal antecedents of engagement, motivational and cognitive indicators of engagement, and participatory engagement. We estimate these associations using our primary measure of online political hostility and the two secondary robustness measures, AFINN sentiment scores and Perspective API toxicity scores.

Fig 1 demonstrate positive and statistically significant associations between online political hostility and several indicators of political engagement and its antecedents (consistent with Expectation 1). The associations were obtained after controlling for broad personality traits, income, age, gender, and party identification. The largest associations are generally observed for the primary measure of political hostility rather than for the more general AFINN or Perspective API measures. The associations with online participation and political interest are, for example, 0.236 (95% CI [0.186; 0.285]) and 0.306 (95% CI [0.247; 0.366]) for online political hostility, whereas they are 0.176 (95% CI [0.103; 0.249]) and 0.170 (95% CI [0.086; 0.253]) for AFINN scores and 0.267 (95% CI [0.212; 0.322]) and 0.239 (95% CI [0.170; 0.308]) for Perspective API scores. This pattern is consistent with the argument that the relevant behavior is specifically political hostility rather than negativity or toxicity in general.

In terms of magnitude, the standardized coefficients for the associations between indicators of political engagement and the measure of online political hostility are generally above 0.2. This puts them on par with the association generally observed between education and political participation [18]. The associations between political hostility and the control variables is not our focus here but detailed estimates are reported in S1 Appendix.

### Robustness tests

Fig 2 provides a replication of the analyses above where the composite dependent variable of online political hostility is subdivided into the two components of “political” and “hate” (i.e., general hostility). In addition, as described above, we also use an alternative supervised classifier of online political hostility based on a sample of hand labelled posts.

The robustness analyses are broadly consistent with the main pattern, with significant and sizeable associations across all three measures. The associations are, not surprisingly, larger for the “political” measure since this is a direct measure of political engagement.

As a further robustness test we also created a model where we interact the Perspective API toxicity scores and AFINN scores with a political relevance score (based on the cosine similarity between individual posts and a “politics” word vector created using the same word embedding approach as described above). The results from these analyses are presented in S1 Appendix and show that at high levels of political relevance, the association between toxicity and political hostility is significantly larger than at low levels of political relevance, except for education. This is consistent with the main association between political engagement and online political hostility.

Additionally, we have also created a model where we control for the share of political posts among all posts posted by each user. The results from these analyses are presented in S1 Appendix and show that the main results are robust to this additional control; if anything the results are slightly stronger when controlling for the share of political posts. This suggests that the results are not simply driven by more politically engaged users posting more about politics.

### Leader-directed affective polarization and conditional associations

Expectation 2 concerns whether the associations between engagement and hostility are stronger among users with higher leader-directed affective polarization. Fig 4 displays the associations separately for users above and below the midpoint of the leader-directed affective-polarization scale. The descriptive pattern suggests that the association between engagement and hostility is often stronger among more affectively polarized users: for those low on affective polarization only four out of eighteen associations, across our three measures of online hostility, are significant, whereas for those high on affective polarization fourteen out of eighteen are significant.

However, the moderation evidence is not uniform across all indicators. Formal interaction models (Fig 3) show the clearest support for online and offline participation, while the evidence is weaker or inconsistent for more distal antecedents such as education (see S1 Appendix for full results). Thus, the results are partially consistent with Expectation 2 rather than a uniform confirmation of it. This distinction is especially important because leader-directed affective polarization may be an antecedent, correlate, or consequence of hostile online interaction. We therefore interpret these analyses as suggestive evidence of conditional associations, not as causally identified moderation effects. The multiplicity-adjusted results reported in S1 Appendix lead to the same cautious interpretation: of the eighteen interaction terms, eight remain significant after a Benjamini–Hochberg correction, and these are concentrated among the participatory engagement indicators. The conditional pattern should therefore be treated as exploratory for the remaining indicators.

### Temporal within-person associations from RI-CLPM models

The previous analyses are associational and primarily cross-sectional. To examine whether the same pattern appears in temporal within-person models, we use RI-CLPMs with behavioral measures of online political engagement and online hostility. These models account for stable time-invariant differences between users, but they do not rule out time-varying confounding or establish causal effects.

The full RI-CLPM results are reported in S1 Appendix. In brief, within-person increases in online political engagement are followed by higher levels of online political hostility across the three hostility measures. We also observe some evidence of the reverse temporal association, in which hostility is followed by higher political engagement, although this pattern is weaker and less consistent. These results are consistent with the main associations while underscoring that the relationship may be reciprocal.

### Exploratory implications for activity and potential reach

The traditional literature on political engagement often finds that more engaged citizens have greater opportunities to make their voices heard [18,19]. Given the associations above, we therefore turn to our exploratory expectation and examine whether users who express more online political hostility are also more active on X/Twitter and have greater potential reach. These analyses are exploratory and descriptive.

We investigate the visibility of politically hostile users by first showing results from count models that examine whether those who are politically hostile (a) post more, (b) have more friends, and (c) have more followers. A “friend” in X’s usage is someone I follow, and a “follower” is someone who follows me. For completeness, we include exploratory descriptive network visualizations of the study participants’ follower/friend graph in S1 Appendix but do not draw inferences about centrality or community structure.

The count-model results indicate that higher political hostility is associated with more posts and with larger numbers of followers and, to a lesser extent, friends. A change from the mean level of hostility to 1.5 standard deviations above the mean is associated with an increase in the number of posts from around 420 to around 900 and an increase in the number of followers from around 220 to around 370. These results suggest greater activity and potential reach, but they should not be interpreted as evidence of formal network influence or centrality.

## Discussion

The advent of the Internet has transformed contemporary political life. Platforms such as X/Twitter, Facebook, and YouTube have expanded opportunities for citizens to participate in political debate, while also making hostile exchanges between political opponents highly visible. The goal of this study has been to identify correlates of hostile political expression among American X/Twitter users.

Building on classical theories of political engagement [17,19] and recent work on the darker sides of engagement, we find that online political hostility is positively associated with several indicators of political engagement and its antecedents. These include distal antecedents such as education and internal efficacy, motivational and cognitive indicators such as political interest and political knowledge, and more direct indicators such as online and offline participation. These findings suggest that online political hostility is not only associated with deprivation, marginalization, or personality-based explanations, as highlighted in prior work [14,15]. It is also associated with forms of engagement that are often considered central to democratic citizenship.

This pattern is consistent with recent research on motivated reasoning, partisan animus, and the darker sides of political engagement [21,33,47,82]. In a polarized political context, politically engaged citizens may be more likely to enter conflictual political exchanges, and affective polarization may make those exchanges more antagonistic. At the same time, our evidence does not establish that engagement causes hostility. Political sophistication, partisan involvement, affective polarization, or hostile online interactions themselves may all shape the observed associations.

The exploratory analyses of the activity and reach of hostile users add a visibility implication. Users who express more online political hostility also post more frequently and have more friends and followers. This suggests that hostile political expression may be more visible on X/Twitter not only because hostile content attracts attention, but also because users who express such hostility are more active and have greater potential reach [3]. This implications notwithstanding, we do not draw conclusions about formal network influence, centrality, or community structure.

Several limitations are important. First, the data come from American X/Twitter users. This platform is a public, text-based, and highly political platform, and the associations observed here may not generalize directly to platforms with different affordances, audiences, moderation systems, or norms of interaction. Cross-platform research is needed to assess whether similar patterns appear on Facebook, Reddit, YouTube, TikTok, Instagram, or private messaging environments. Some tentative evidence of broader applicability is provided in S1 Appendix, where online political hostility in a sample of Danish X/Twitter users is also positively associated with users’ political interest, internal efficacy, and news consumption, and where the relationship extends – to a lesser extent – to users with negative views of the political system [14,50].

Second, the analyses are observational. The RI-CLPM analyses reduce concerns about stable time-invariant confounding by focusing on within-person change, but they do not rule out time-varying confounding, reciprocal relationships, or measurement error. Political sophistication or partisan involvement may be common causes of both participation and hostility. Affective polarization may increase engagement, engagement may increase affective polarization, and hostile online interaction may itself intensify affective polarization. Consistent with reciprocal influences, recent research suggests that the association of a user’s hostility with subsequent partisan extremity is bidirectional, although the association from the former to the latter is stronger than the reverse [83]. The findings should overall be interpreted as associational rather than causal.

Third, and relatedly, the present findings are mute on the exact causal relationships between the different indicators of political engagement and their antecedents. We interpret education and internal efficacy should be interpreted as distal antecedents of engagement rather than as direct causes of hostility. Political interest, political knowledge, and participation are more proximate to hostile political expression. An important caveat in this respect is that the analyses should not be taken to imply that, for example, more educated users are more hostile absent their generally higher levels of political engagement.

Fourth, although the primary measure of political hostility is supported by several validation exercises and robustness checks, no automated text-based measure perfectly captures the underlying construct. The measure may partly capture proximity to politically salient conflict language or hate-related political vocabulary rather than hostility alone. We therefore interpret the results in light of the validation evidence and robustness checks, while acknowledging this remaining construct-validity limitation.

Fifth, due to data availability, our measure of affective polarization captures feelings toward party elites rather than toward parties as institutions or ordinary party supporters, as is standard [22]. The moderation analyses should therefore be interpreted as evidence about *leader-directed* affective polarization. Future research should examine whether the same conditional patterns appear using party-directed or supporter-directed measures.

Finally, while we argue that political engagement is associated with political hostility over and beyond personality, as highlighted in prior research, the utilized Big Five controls do not directly test personality-based accounts centered on more antisocial traits such as Dark Triad traits, sadism, or other antagonistic traits. Such traits were not not measured in the survey. Future work should examine how political engagement interacts with these more specific personality constructs.

These findings have implications for efforts to reduce online political hostility. Platform design, moderation, ranking systems, and friction-based interventions may all matter. However, if online political hostility is also associated with political engagement and out-party animus, platform-level interventions alone may be insufficient. Reducing hostile political engagement online may also require attention to broader political polarization and the role of political elites in shaping norms of partisan conflict.

## Supporting information

S1 Appendix Supplementary methods and results. This appendix contains: (1) Reproducing traditional relationships regarding political engagement; (2) Additional methods and results; (3) Exploratory results for network analyses; (4) Additional results for count models; (5) Missing data; (6) Ethical considerations; (7) Random Intercept Cross-Lagged Panel Models for Behavioral Measures of Online Engagement; and (8) Additional correlations for Denmark.
(PDF)
