# Supplementary material for: Political engagement and online political hostility among American X/Twitter users
Source: PLoS One. 2026 Nov 11;21(11):e0360061. doi: 10.1371/journal.pone.0360061 (PMC13754149; doi:10.1371/journal.pone.0360061)
Supplement: Supplementary file 1 [file pone.0360061.s001.pdf]

S1 Appendix: Supplementary Methods and Results

Contents

1 Appendix 1: Reproducing traditional relationships regarding political engagement 2

2 Appendix 2: Additional methods and results 4
2.1 Measures of political engagement . . . 4
2.1.1 Internal efficacy . . . 4
2.1.2 Education . . . 4
2.1.3 Political interest . . . 4
2.1.4 Political knowledge . . . 4
2.1.5 Political participation . . . 4
2.2 Measures used for statistical control . . . 5
2.2.1 Personality traits . . . 5
2.2.2 Party identification . . . 5
2.2.3 Demographics . . . 5
2.3 Measure of affective polarization . . . 5
2.4 Additional results for the association between indicators of political engagement, its antecedents, and online hostility . . . 6
2.5 Multiple-comparison adjustments . . . 10
2.6 Factor analysis of the items for political engagement . . . 12
2.7 Additional correlations . . . 15
2.8 Additional results and outputs . . . 18

3 Appendix 3: Exploratory results for network analyses 35
3.1 Measures . . . 35
3.2 Results . . . 35

4 Appendix 4: Additional results for count models 38
4.1 Methods and measures . . . 38
4.2 Results . . . 38
4.2.1 Counts of tweets, friends, and followers . . . 38

5 Appendix 5: Missing data 40

6 Appendix 6: Ethical considerations 41

7 Appendix 7: Random Intercept Cross-Lagged Panel Models for Behavioral Measures of Online Engagement 42
7.1 Methods and measures . . . 42
7.2 Results . . . 43

8 Appendix 8: Additional correlations for Denmark 45

1 Appendix 1: Reproducing traditional relationships regarding political engagement

In this section we examine the traditionally expected patterns of correlations regarding political engagement: Do education and internal efficacy propel citizens to become more likely to engage in traditional forms of (offline) political participation?

To this end, we provide a simple correlation table to reproduce the results from the numerous studies demonstrating the strong relationships between distal antecedents of political engagement and measures of political participation [1]. We report Spearman correlations to avoid assuming that all measures are necessarily normally distributed. The correlations are based on a dataset grouped at the level of the individual X user and thus do not take the panel aspect into account. We use pairwise deletion to include as many observations as possible for the full table.

Results that are not statistically significant at a .05 level are reported in gray and results that are statistically significant are reported in white.

As expected, there is a strong relationship between internal efficacy and a classical measure of political engagement, namely "offline" participation (r=0.34). Higher levels of education are also associated with higher levels of "offline" participation (r=0.24). Both education (r=0.22) and internal efficacy (r=0.53) are also positively correlated with political interest. Further, the figure also shows that both variables correlate positively with *online* forms of political participation, providing the first clue that these factors may also shape political behaviors in social media interactions.

It is also worth noting that income, openness, and extroversion are all significantly and positively correlated with offline participation, which is also in line with previous studies [2,3].

Generally, the findings demonstrate that this sample of participants is similar to the samples from numerous other studies suggesting that internal efficacy and education are relevant predictors of political participation.

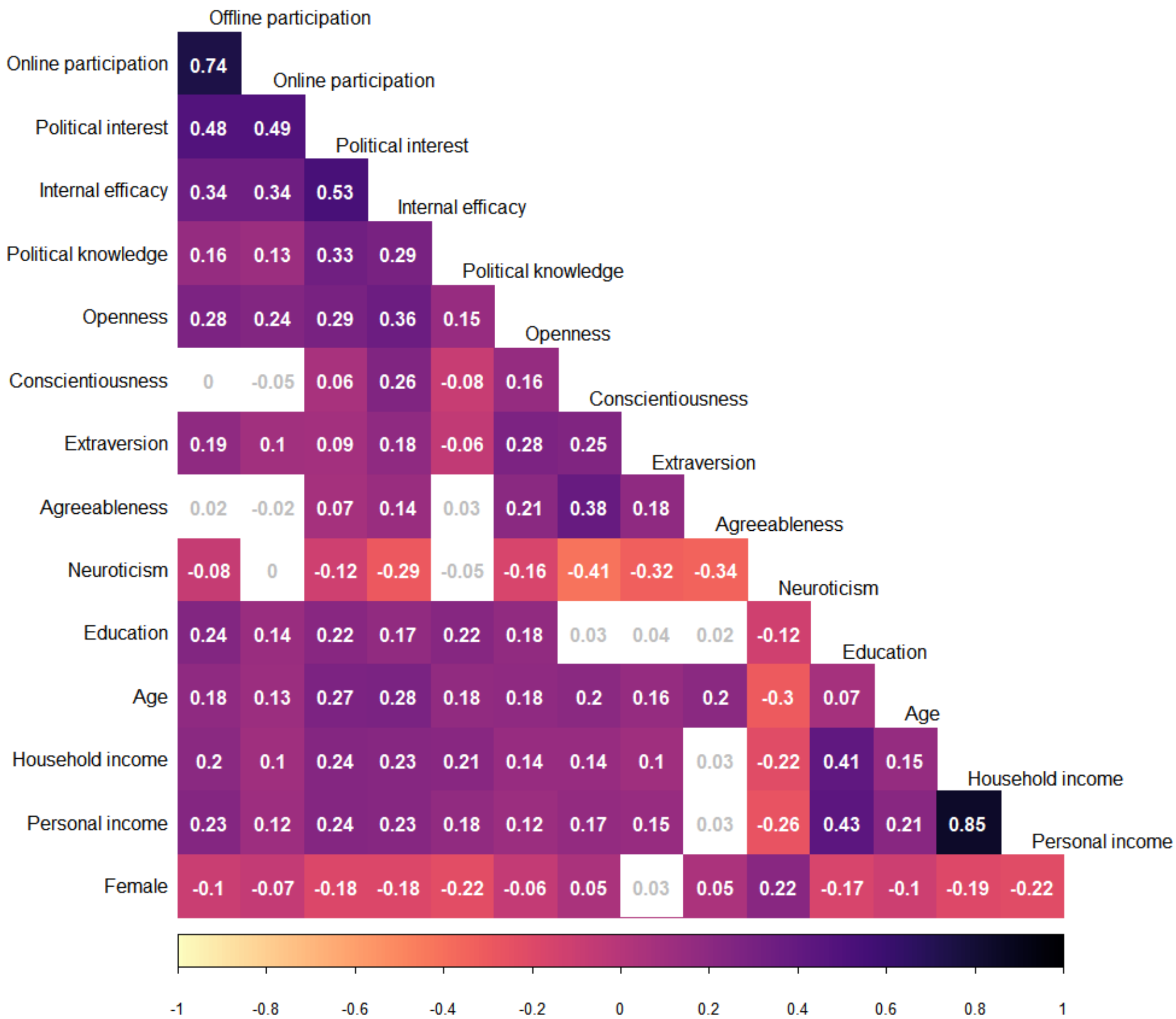

	Offline participation	Online participation	Political interest	Internal efficacy	Political knowledge	Openness	Conscientiousness	Extraversion	Agreeableness	Neuroticism	Education	Age	Household income	Personal income
Online participation	0.74													
Political interest	0.48	0.49												
Internal efficacy	0.34	0.34	0.53											
Political knowledge	0.16	0.13	0.33	0.29										
Openness	0.28	0.24	0.29	0.36	0.15									
Conscientiousness	0	-0.05	0.06	0.26	-0.08	0.16								
Extraversion	0.19	0.1	0.09	0.18	-0.06	0.28	0.25							
Agreeableness	0.02	-0.02	0.07	0.14	0.03	0.21	0.38	0.18						
Neuroticism	-0.08	0	-0.12	-0.29	-0.05	-0.16	-0.41	-0.32	-0.34					
Education	0.24	0.14	0.22	0.17	0.22	0.18	0.03	0.04	0.02	-0.12				
Age	0.18	0.13	0.27	0.28	0.18	0.18	0.2	0.16	0.2	-0.3	0.07			
Household income	0.2	0.1	0.24	0.23	0.21	0.14	0.14	0.1	0.03	-0.22	0.41	0.15		
Personal income	0.23	0.12	0.24	0.23	0.18	0.12	0.17	0.15	0.03	-0.26	0.43	0.21	0.85	
Female	-0.1	-0.07	-0.18	-0.18	-0.22	-0.06	0.05	0.03	0.05	0.22	-0.17	-0.1	-0.19	-0.22

Table 1: Correlations with political engagement

2 Appendix 2: Additional methods and results

Generally speaking, we use Mplus [4] for all statistical models and the statistical computing environment R [5] to connect to Mplus using the MplusAutomation package [6]. Most graphs use the plotting library ggplot2 [7], except the network graphs, where we use R's built-in plotting functionality using the igraph package [8].

2.1 Measures of political engagement

In order to make the investigation of the associations between indicators of political engagement, its antecedents, and online political hostility as comprehensive as possible, we investigate a broad range of measures of political engagement. In essence, we can distinguish between more distal antecedents of political engagement, such as education and internal efficacy, and more proximate indicators of political engagement such as online and offline political participation.

2.1.1 Internal efficacy

Internal efficacy is measured using the standard Center for Political Studies (CPS) questions [9]: "Sometimes politics seem so complicated that a person like me can't really understand what's going on" and "I feel that I have a pretty good understanding of the important political issues facing our country." We also use a third item from the European Social Survey that asks whether "I often think it is difficult to make up my mind about political issues." Cronbach's alpha is 0.70 for our measure of internal efficacy.

2.1.2 Education

Education is measured using six different educational categories: No HS, High school graduate, Some college, 2-year, 4-year, Post-grad.

2.1.3 Political interest

Political interest is measured using a standard American National Election Studies (ANES) question asking respondents about their level of political interest on a five-point scale ranging from "Extremely interested" to "Not interested at all."

2.1.4 Political knowledge

Political knowledge is measured using five factual questions from the ANES, scored as the number of correct answers.

2.1.5 Political participation

To fully capture the many ways citizens engage in politics, we include measures of both online and offline political participation. To measure traditional *offline* participation, we created an 11-item battery based on previous research [**?**,]]Oser2012, GildeZiga2014, asking participants on scales ranging from "1 = Never" to "7 = Several times a day" how often they participate in eleven offline political activities, like"Contact a government official in person, by phone, or by letter," "Work or volunteer for a political party, candidate or a social cause," and "Wear clothes or use items in support of a political or social cause." To measure *online* participation, we asked participants on the same response scale how often they engaged in ten online political activities [**?**,]]Feezell2016, Valenzuela2019, including "Comment on others' online posts about a political or social cause (including online news articles)," "Like or follow a page of a political group, party or candidate on social media," and "Start or join a political group or group supporting a cause on a social networking site."

2.2 Measures used for statistical control

2.2.1 Personality traits

In assessing the empirical support for our expectations, it is key to account for alternative explanatory factors. As described in the main text, a widespread account stresses that online political hostility reflects the presence of emotional, aggressive, and volatile individuals. We therefore include the Big Five personality traits as broad controls for stable individual differences in motivational, emotional, and cognitive dispositions to think, act, and feel [10–12]. These controls are not a direct test of Dark Triad, sadism, or other antagonistic-trait accounts, which were not measured in the survey. The Big Five nonetheless capture affectively relevant dispositions: neuroticism, with its proneness to experience negative emotionality, anxiety, and sensitivity towards punishment, as well as extroversion, with its tendency to experience positive emotionality and reward sensitivity, have a strong affective component [13, 14], and both neuroticism and extroversion have consistently been found to relate to mood [14–17].

We rely on the Big Five Inventory (BFI) consisting of 44 items to measure the five major personality traits [12,].The alpha reliability was 0.82 for openness, 0.84 for conscientiousness, 0.87 for extroversion, 0.82 for agreeableness, and 0.87 for neuroticism.

2.2.2 Party identification

In addition to the measures described above, we also include a measure of party identification in order to rule out the possibility that our findings on political engagement reflect underlying partisan dispositions. To measure partisanship, we used a standard question from the American National Election Study, asking "Generally speaking, do you usually think of yourself as a Republican, Democrat, Independent, Other?" Participants who answered either "Republican" or "Democrat" were then asked whether they saw themselves as "Strong" or "Not very strong" supporters of their party, while those answering "Independent" or "Other" were asked whether they saw themselves as closer to one of the parties. Based on participants' responses, we then created our partisanship measure on a seven-point scale, ranging from "Strong Democrat" to "Strong Republican."

2.2.3 Demographics

Standard demographics of age, gender and income were obtained from YouGov's background information about its panelists. Age is measured in age brackets of five-year intervals starting with 18-22 and ending with 83+. Our gender variable "Female" is a binary variable containing the categories "Male" and "Female" and indicates whether someone self-reported as female or not. Household income is measured in intervals of \$10,000 starting from "less than \$10,000" and ending with "\$500,000 and more." Controlling for income in particular is important, as alternative theoretical perspectives highlight the lack of more tangible resources (such as money) as a main driver of political engagement.

2.3 Measure of affective polarization

To explore conditional associations between online hostility and political engagement, we use a measure of leader-directed affective polarization. Affective polarization typically relates to animosity towards opposing political groups, especially party elites [18], and is here measured using feeling thermometer ratings of political leaders, calculating the difference between self-reported in-party and out-party leader ratings [19]. A higher score means the person is *more* polarized and vice versa. We asked questions about three Republican politicians (Ted Cruz, Donald Trump, and Mike Pence) and three Democratic politicians (Joe Biden, Elizabeth Warren, and Bernie Sanders). We then use the respondents' answers on party identification to distinguish whether they self-identify as Republicans or Democrats. Because the survey did not include parallel feeling thermometer ratings of ordinary party supporters, this measure captures affect toward party elites rather than parties as institutions or party supporters. The question asked was: "We'd like to get your feelings toward some of our political leaders and other people who are in the news these days on a 'feeling thermometer.' A rating of zero degrees means you feel as cold and negative as possible. A rating of 100 degrees means you feel as warm and positive as possible. You would rate the politician at 50 degrees if you don't feel particularly positive or negative toward the politician."

2.4 Additional results for the association between indicators of political engagement, its antecedents, and online hostility

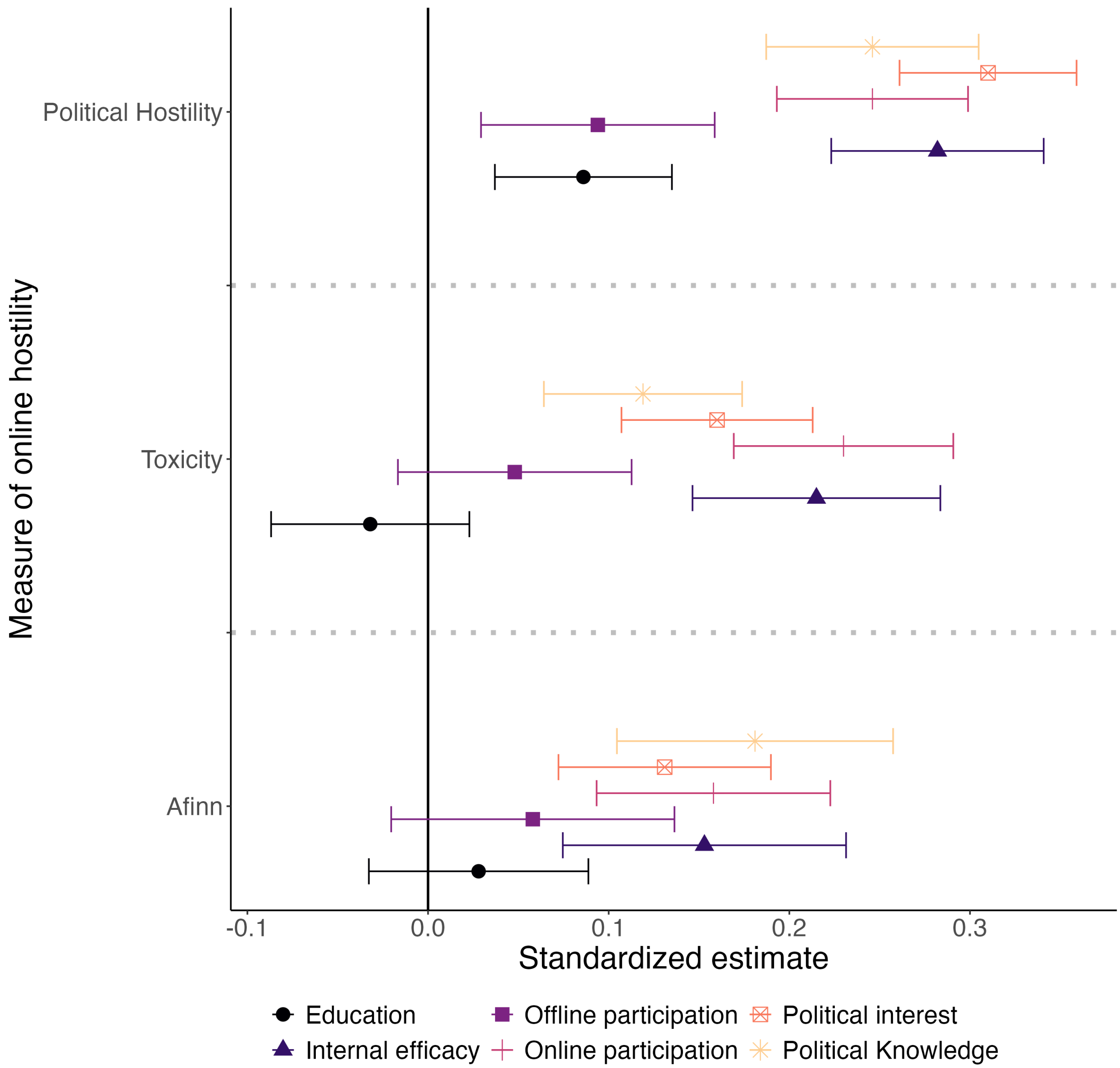

Figure 1: **The association between indicators of political engagement, its antecedents, and online political hostility while also controlling for share of political tweets.** All models control for the Big Five personality traits, income, age, gender, and party identification as well as the share of political tweets. Error bars represent 95% confidence intervals. The share of politcal tweets variable has been constructed by using the political SU vector and labeling all those above the median value in non-aggregated tweet dataset as "political" and gave it a score of one. The sum of this is divided by the total number of tweets, for a given user, to calculate the proportion of political tweets for a user

Figure 2 illustrates the interaction models between Perspective API scores and Political using the SU approach. If the interaction term is significant the association between Perspective API scores and political hostility is stronger at higher levels of Political. This is what we see for all measures, although the inter-

action term is only significant when it comes to Internal Efficacy 0.146 (95 % CI [0.128;0.164]) and Online Participation 0.012 (95 % CI [0.043;0.173]). The exception is education, a slightly more distal antecedent of engagement -.076 (95 % CI [-0.121;-0.03])

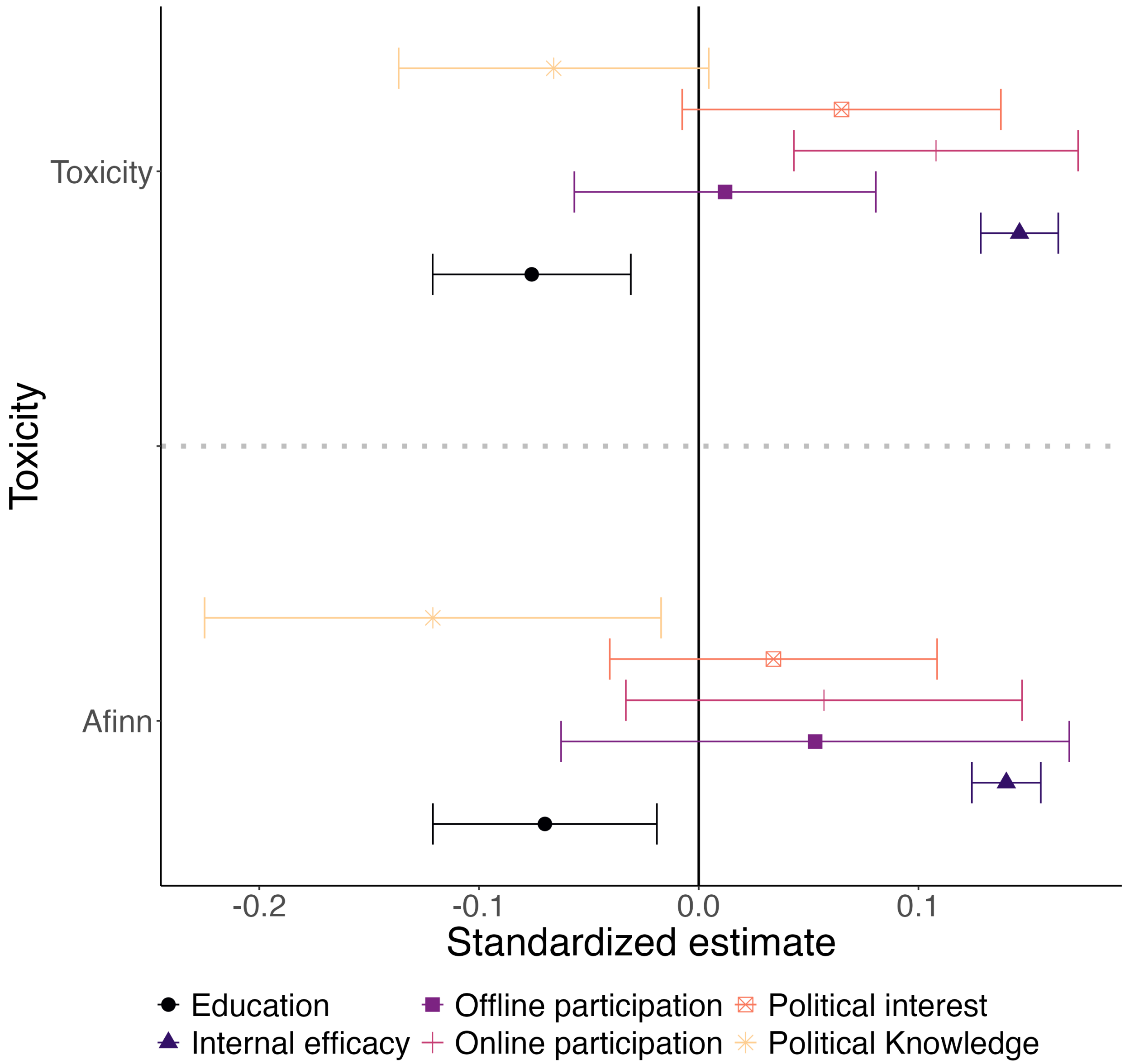

Figure 2: Interaction coefficients for Perspective API scores and political relevance (*Political*). All models control for age and gender but not the full set of controls to reduce problems of multicollinearity.

Figure 3 displays the coefficient from the *interaction* between the engagement indicators from Figure **??** and the measure of leader-directed affective polarization; if the interaction term is significant, the association of that indicator with online hostility differs by levels of leader-directed affective polarization. A positive value indicates that the association of the indicator with online hostility is larger when affective polarization is higher. From Figure 3 the evidence is partial rather than uniform: eight out of eighteen interaction coefficients are significant and in the expected direction. In general, the indicators most strongly associated with online hostility, i.e. online participation and offline participation, are also those that are consistently significant

across model specifications. This is not surprising since these also have the highest statistical power. The same eight interaction terms remain significant after a Benjamini–Hochberg multiplicity correction within this family (see Table 2 and the dedicated multiple-comparisons section below). Because affective polarization may itself be an antecedent, correlate, or consequence of hostile online interaction, these interaction patterns should be read as descriptive conditional associations rather than as causally identified moderation.

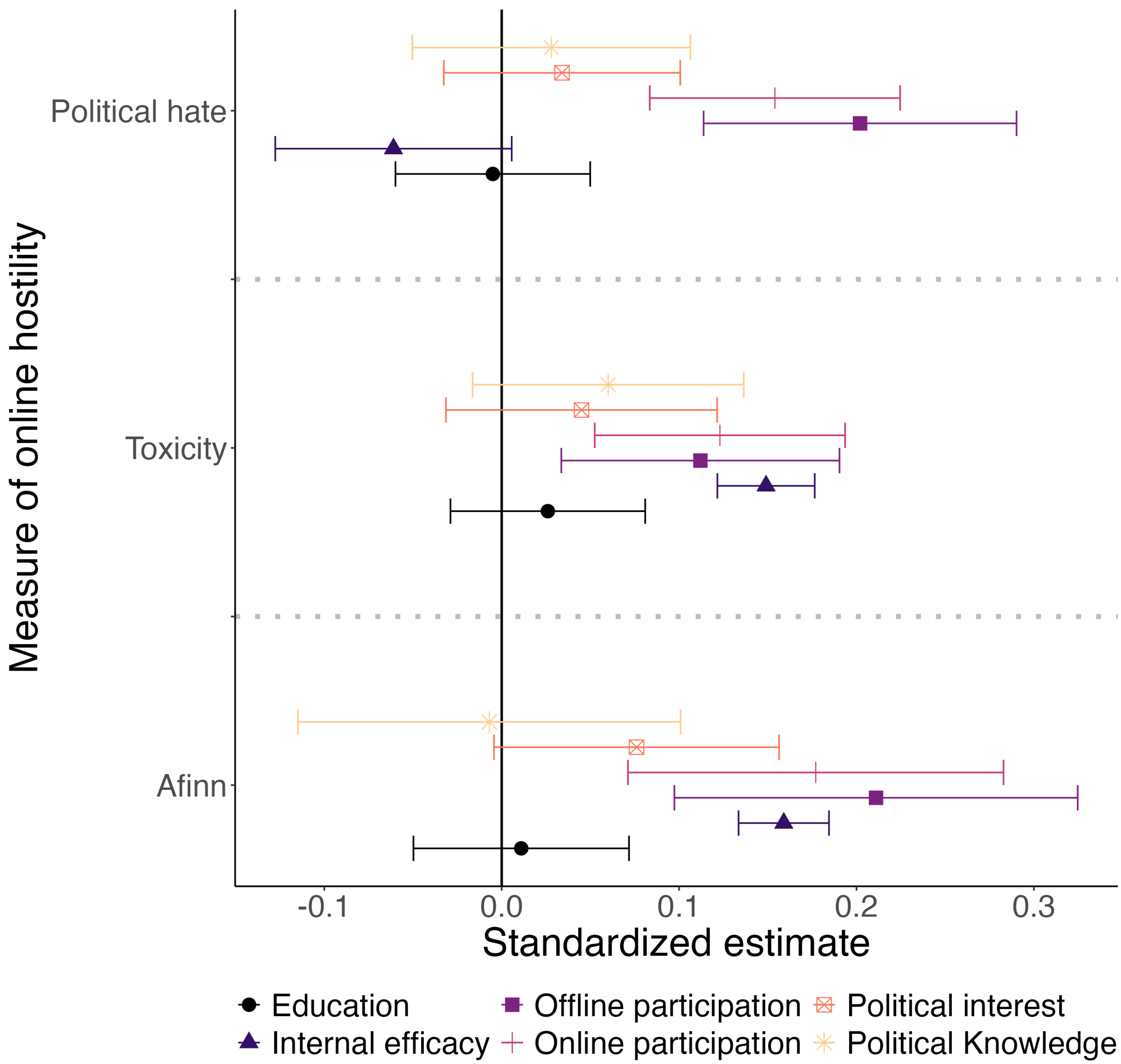

Figure 3: Interaction coefficients between indicators of political engagement, its antecedents, and leader-directed affective polarization. All models control for age and gender but not the full set of controls to reduce problems of multicollinearity. The figure illustrates the *interaction* coefficient from each model. Full results can be found in Table 10 in appendix 2

A sense of the size of the interaction coefficient can also be assessed from the graph (full marginal plots with more information are available in Figure 16 in Appendix 2). For instance, the standardized association of offline participation with toxicity changes from -0.004 (95 % CI [-0.130;0.121] to 0.471 (95 % CI [0.300;0.641])

when going from 1.5 standard deviations below to above the mean of the moderating variable. This is a standardized and large conditional association. Not all associations are of this size, but the conditional associations of online and offline participation across our measures of online hostility are quite large.

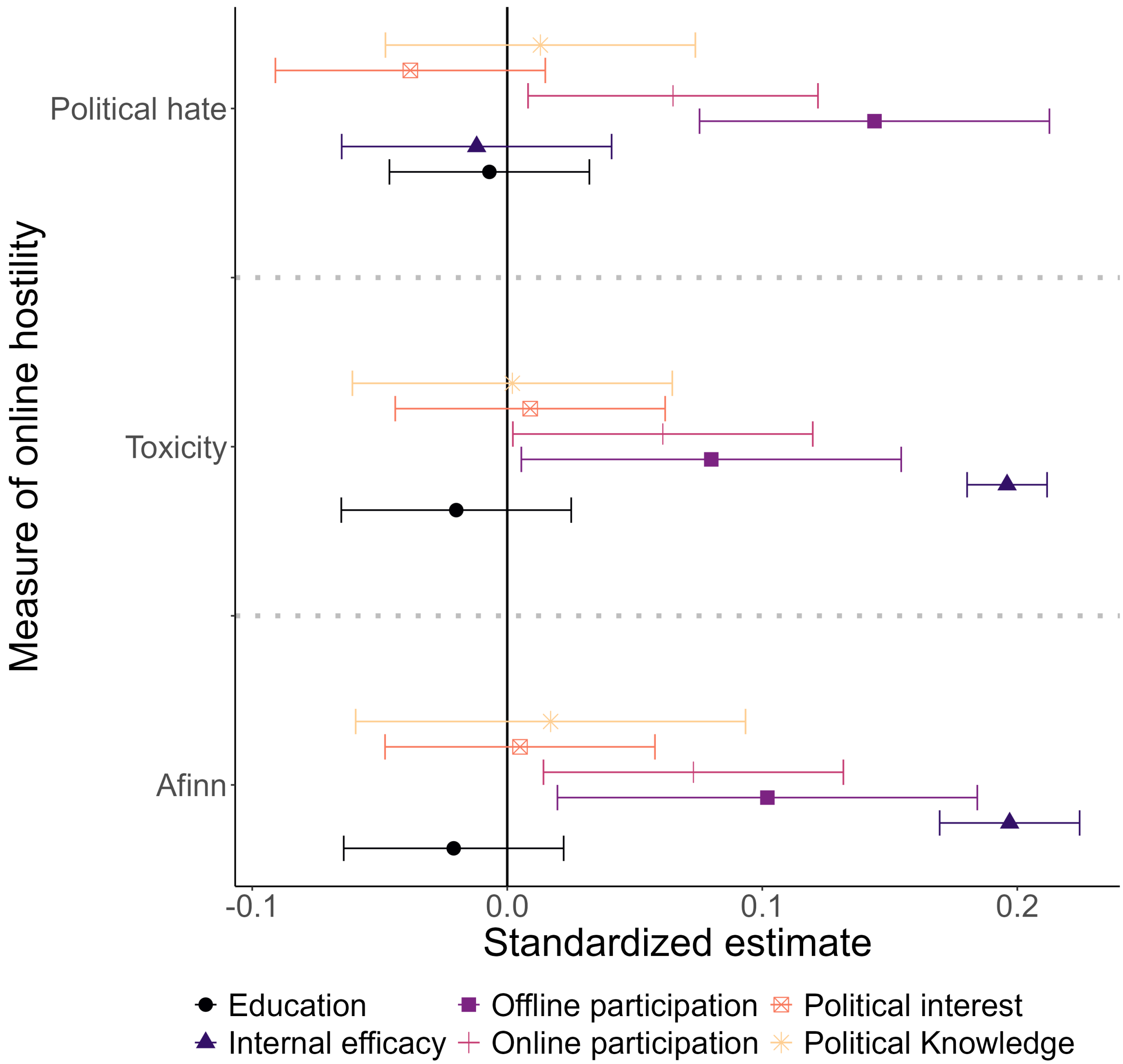

Figure 4: Interaction coefficients between indicators of political engagement, its antecedents, and *cynicism*. All models control for age and gender but not the full set of controls to reduce problems of multicollinearity.

Figure 4 provides a replication of the interaction analyses using a measure of political cynicism rather than affective polarization. We measured political cynicism via an eight-item survey instrument [20], asking participants whether they agreed or disagreed with statements like "Politics in the United States is sick" ($\alpha = 0.74$).

2.5 Multiple-comparison adjustments

Because the analyses involve many tests of closely related expectations, we assess the robustness of the key conclusions to multiple-comparison adjustments. We define four families of tests: (1) the primary association models using the SU measure of online political hostility; (2) the robustness outcome models using AFINN sentiment, Perspective API toxicity, and the supervised classifier; (3) the leader-directed affective-polarization interaction models; and (4) the exploratory activity/reach count models. Within each family we apply a Benjamini–Hochberg false-discovery-rate (FDR) correction to the (already estimated) two-tailed p-values, and we additionally report Holm-adjusted p-values and Bonferroni-adjusted 95% confidence intervals as more conservative alternatives. No models are re-estimated; the adjustments are computed from the estimates and standard errors reported by the original models.

Table 2 reports the results. All six primary associations survive every adjustment. Among the robustness outcomes, fourteen of eighteen associations remain significant under FDR (the exceptions are education throughout and offline participation for the AFINN measure). For the affective-polarization interactions, eight of eighteen interaction terms survive FDR, and these are concentrated among the participatory engagement indicators (online and offline participation) together with internal efficacy for the AFINN and toxicity measures; the remaining interactions should be interpreted as exploratory. All three exploratory activity/reach associations survive adjustment.

Table 2: Multiplicity-adjusted results by family. Unadjusted 95% CIs and p-values are shown alongside Benjamini–Hochberg q-values, Holm-adjusted p-values, and Bonferroni-adjusted 95% CIs computed within each family. "Surv. FDR" indicates the coefficient survives the Benjamini–Hochberg correction ($q < 0.05$). For the affective-polarization family, the coefficient is the interaction term; for the count models, it is the unstandardized slope of political hostility.

**Outcome**	**Predictor**	**Est.**	**95% CI**	p	q **(BH)**	**Holm** p	**Bonf. 95% CI**	**Surv. FDR**
**1. Primary (SU political hostility)**								
Political hostility (SU)	Education	0.072	[0.035, 0.109]	¡0.001	¡0.001	¡0.001	[0.022, 0.122]	Yes
Political hostility (SU)	Internal efficacy	0.226	[0.189, 0.263]	¡0.001	¡0.001	¡0.001	[0.176, 0.276]	Yes
Political hostility (SU)	Political interest	0.306	[0.247, 0.365]	¡0.001	¡0.001	¡0.001	[0.227, 0.385]	Yes
Political hostility (SU)	Online participation	0.236	[0.187, 0.285]	¡0.001	¡0.001	¡0.001	[0.170, 0.302]	Yes
Political hostility (SU)	Offline participation	0.094	[0.031, 0.157]	0.004	0.004	0.004	[0.010, 0.178]	Yes
Political hostility (SU)	Political knowledge	0.233	[0.159, 0.307]	¡0.001	¡0.001	¡0.001	[0.133, 0.333]	Yes
**2. Robustness outcomes**								
AFINN	Education	0.041	[-0.016, 0.098]	0.149	0.168	0.508	[-0.046, 0.128]	No
AFINN	Internal efficacy	0.175	[0.053, 0.297]	0.005	0.006	0.025	[-0.010, 0.360]	Yes
AFINN	Political interest	0.170	[0.086, 0.254]	¡0.001	¡0.001	¡0.001	[0.041, 0.299]	Yes
AFINN	Online participation	0.176	[0.103, 0.249]	¡0.001	¡0.001	¡0.001	[0.065, 0.287]	Yes
AFINN	Offline participation	0.065	[-0.017, 0.147]	0.127	0.152	0.508	[-0.061, 0.191]	No
AFINN	Political knowledge	0.169	[0.089, 0.249]	¡0.001	¡0.001	¡0.001	[0.046, 0.292]	Yes
Perspective toxicity	Education	-0.015	[-0.064, 0.034]	0.557	0.590	1.000	[-0.090, 0.060]	No
Perspective toxicity	Internal efficacy	0.212	[0.112, 0.312]	¡0.001	¡0.001	¡0.001	[0.059, 0.365]	Yes
Perspective toxicity	Political interest	0.239	[0.170, 0.308]	¡0.001	¡0.001	¡0.001	[0.134, 0.344]	Yes
Perspective toxicity	Online participation	0.267	[0.212, 0.322]	¡0.001	¡0.001	¡0.001	[0.183, 0.351]	Yes
Perspective toxicity	Offline participation	0.094	[0.035, 0.153]	0.002	0.003	0.016	[0.004, 0.184]	Yes
Perspective toxicity	Political knowledge	0.145	[0.069, 0.221]	¡0.001	¡0.001	¡0.001	[0.028, 0.262]	Yes
Supervised classifier	Education	-0.006	[-0.041, 0.029]	0.734	0.734	1.000	[-0.060, 0.048]	No
Supervised classifier	Internal efficacy	0.145	[0.102, 0.188]	¡0.001	¡0.001	¡0.001	[0.079, 0.211]	Yes
Supervised classifier	Political interest	0.224	[0.150, 0.298]	¡0.001	¡0.001	¡0.001	[0.110, 0.338]	Yes
Supervised classifier	Online participation	0.218	[0.161, 0.275]	¡0.001	¡0.001	¡0.001	[0.131, 0.305]	Yes
Supervised classifier	Offline participation	0.110	[0.041, 0.179]	0.002	0.003	0.016	[0.005, 0.215]	Yes
Supervised classifier	Political knowledge	0.136	[0.052, 0.220]	0.002	0.003	0.016	[0.007, 0.265]	Yes
**3. AP interaction (leader-directed)**								
Political hostility (SU)	Education	-0.005	[-0.060, 0.050]	0.866	0.900	1.000	[-0.089, 0.079]	No
Political hostility (SU)	Internal efficacy	-0.061	[-0.128, 0.006]	0.074	0.133	0.666	[-0.163, 0.041]	No
Political hostility (SU)	Political interest	0.034	[-0.033, 0.101]	0.317	0.439	1.000	[-0.068, 0.136]	No
Political hostility (SU)	Online participation	0.154	[0.083, 0.225]	¡0.001	¡0.001	¡0.001	[0.046, 0.262]	Yes
Political hostility (SU)	Offline participation	0.202	[0.114, 0.290]	¡0.001	¡0.001	¡0.001	[0.067, 0.337]	Yes
Political hostility (SU)	Political knowledge	0.028	[-0.050, 0.106]	0.489	0.587	1.000	[-0.092, 0.148]	No
AFINN	Education	0.011	[-0.050, 0.072]	0.732	0.824	1.000	[-0.082, 0.104]	No
AFINN	Internal efficacy	0.159	[0.134, 0.184]	¡0.001	¡0.001	¡0.001	[0.120, 0.198]	Yes
AFINN	Political interest	0.076	[-0.004, 0.156]	0.066	0.132	0.660	[-0.047, 0.199]	No
AFINN	Online participation	0.177	[0.071, 0.283]	0.001	0.003	0.013	[0.015, 0.339]	Yes
AFINN	Offline participation	0.211	[0.097, 0.325]	¡0.001	¡0.001	¡0.001	[0.038, 0.384]	Yes
AFINN	Political knowledge	-0.007	[-0.115, 0.101]	0.900	0.900	1.000	[-0.172, 0.158]	No
Perspective toxicity	Education	0.026	[-0.029, 0.081]	0.347	0.446	1.000	[-0.058, 0.110]	No

continued on next page

Table 2 continued

**Outcome**	**Predictor**	**Est.**	**95% CI**	p	q **(BH)**	**Holm** p	**Bonf. 95% CI**	**Surv. FDR**
Perspective toxicity	Internal efficacy	0.149	[0.122, 0.176]	¡0.001	¡0.001	¡0.001	[0.107, 0.191]	Yes
Perspective toxicity	Political interest	0.045	[-0.031, 0.121]	0.252	0.378	1.000	[-0.072, 0.162]	No
Perspective toxicity	Online participation	0.123	[0.052, 0.194]	0.001	0.003	0.013	[0.015, 0.231]	Yes
Perspective toxicity	Offline participation	0.112	[0.034, 0.190]	0.006	0.013	0.066	[-0.008, 0.232]	Yes
Perspective toxicity	Political knowledge	0.060	[-0.016, 0.136]	0.120	0.196	0.960	[-0.057, 0.177]	No
**4. Exploratory activity/reach (count NB)**								
Number of followers	Political hostility	2.575	[2.148, 3.002]	¡0.001	¡0.001	¡0.001	[2.053, 3.097]	Yes
Number of friends	Political hostility	1.449	[1.084, 1.814]	¡0.001	¡0.001	¡0.001	[1.004, 1.894]	Yes
Number of posts	Political hostility	3.663	[3.104, 4.222]	¡0.001	¡0.001	¡0.001	[2.981, 4.345]	Yes

2.6 Factor analysis of the items for political engagement

We here assess whether our indicators of political engagement can reasonably be seen as reflecting a unidimensional concept and, hence, whether it is preferable to combine the measures into a single scale or whether to treat them as separate measures as is the case in the main text. To this end, we perform a factor analysis of the items for political engagement. Specifically, we use Exploratory Structural Equation Modeling (ESEM), instead of traditional Exploratory Factor Analysis (EFA), which represents a significant advancement in factor analysis techniques, blending elements from EFA and Confirmatory Factor Analysis (CFA) [21]. Traditional EFA is primarily used for exploring the underlying factor structure without imposing a priori constraints. In contrast, CFA is used for testing a predefined factor structure. ESEM bridges these two by allowing for exploratory analysis within a structural equation modeling framework, which incorporates the flexibility of EFA with the rigor of CFA. One of the key differences is the availability of fit indices in ESEM. Traditional EFA does not provide indices like the Comparative Fit Index (CFI), Tucker-Lewis Index (TLI), or Standardized Root Mean Square Residual (SRMR). ESEM, however, enables the computation of these fit indices, offering a more comprehensive evaluation of the model fit. This feature allows researchers to quantitatively assess how well the model represents the data, which is a significant advantage over traditional EFA. ESEM also uses more sophisticated estimation techniques that are common in structural equation modeling, like maximum likelihood or generalized least squares. This contrasts with traditional EFA methods, which often rely on simpler estimation methods such as principal axis factoring or principal component analysis. In terms of fit indices a value greater than 0.96 for CFI is recommended [22], though a value of 0.90 may also be acceptable [23,]. For TLI values should be greater than 0.90 [23] or greater than 0.95 [24,]. For SRMR a value less than 0.08 is indicative of a good fit [23]. Based on this, we can clearly reject the one factor solution, and thus the idea that our measure of political engagement is unidimensional since all fit indices indicate a very poor fit. See Figure 3.

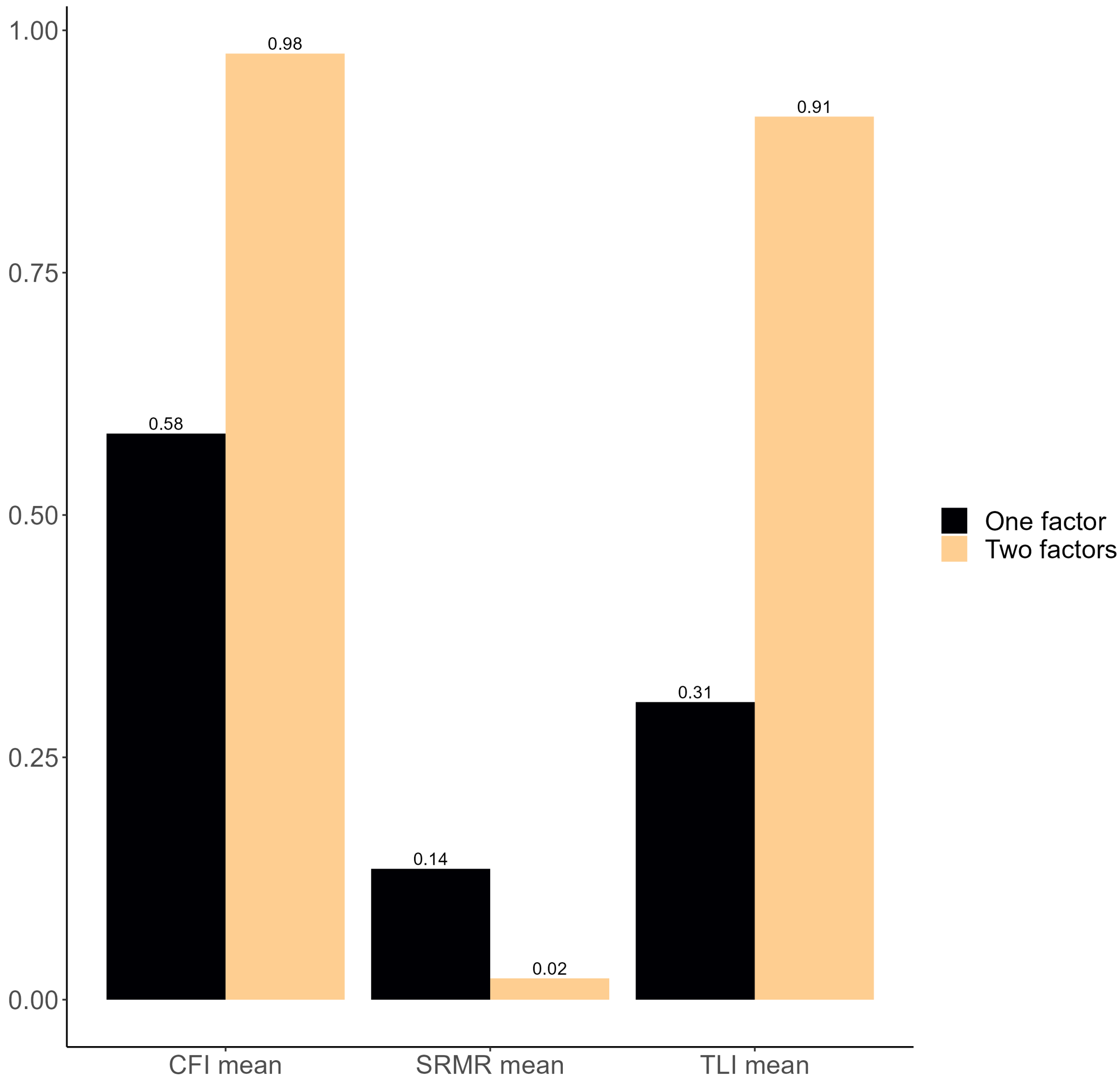

Table 3: Fit statistics for ESEM

Item	Estimates	factor
Education	0.189 (0.037)**	F1
Internal efficacy	0.617 (0.143)**	F1
Political interest	0.538 (0.120)**	F1
Online participation	0.856 (0.131)**	F1
Offline participation	0.764 (0.122)**	F1
Political Knowledge	0.138 (0.161)	F1

Table 4: EFA with one factor for political engagement

Item	Estimates	factor
Education	0.222 (0.045)**	F1
Internal efficacy	0.684 (0.143)**	F1
Political interest	0.586 (0.145)**	F1
Online participation	0.378 (0.370)	F1
Offline participation	0.251 (0.432)	F1
Political Knowledge	0.558 (0.399)	F1
Education	-0.023 (0.165)	F2
Internal efficacy	-0.029 (0.372)	F2
Political interest	-0.040 (0.321)	F2
Online participation	0.530 (0.378)	F2
Offline participation	0.668 (0.374)#	F2
Political Knowledge	-0.526 (0.330)	F2

Table 5: EFA with two factors for political engagement

2.7 Additional correlations

In this section, we provide a comprehensive correlation matrix between the measures utilized for the main analyses. To asssess the stability of these correlations, we provide both correlations for the overall sample as well as for Democrats and Republicans separately.

	Political hate	Political	Hate	Toxicity score	Afinn score	Self-reported hostility	Offline participation	Online participation	Internal efficacy	Political interest	Political knowledge	Openness	Conscientiousness	Extraversion	Agreeableness	Neuroticism	Education	Age	Household income	Personal income
Political	0.92																			
Hate	0.84	0.58																		
Toxicity score	0.54	0.42	0.6																	
Afinn score	-0.49	-0.48	-0.4	-0.61																
Self-reported hostility	0.18	0.14	0.2	0.24	-0.17															
Offline participation	0.29	0.32	0.18	0.18	-0.21	0.34														
Online participation	0.38	0.37	0.29	0.33	-0.27	0.42	0.75													
Internal efficacy	0.31	0.33	0.21	0.24	-0.21	0.05	0.34	0.35												
Political interest	0.39	0.42	0.25	0.25	-0.22	0.11	0.48	0.49	0.53											
Political knowledge	0.27	0.3	0.18	0.16	-0.18	0.01	0.16	0.13	0.29	0.33										
Openness	0.13	0.14	0.09	0.09	-0.05	0.02	0.28	0.24	0.35	0.29	0.15									
Conscientiousness	-0.05	-0.03	-0.07	-0.08	0.05	-0.17	0	-0.05	0.26	0.06	-0.08	0.16								
Extraversion	0.02	0.02	0.01	-0.01	-0.02	-0.02	0.19	0.1	0.19	0.09	-0.06	0.28	0.25							
Agreeableness	-0.02	-0.01	-0.04	-0.07	0.07	-0.23	0.02	-0.02	0.14	0.07	0.02	0.21	0.38	0.18						
Neuroticism	-0.04	-0.08	0.05	0.04	0	0.11	-0.08	-0.01	-0.29	-0.11	-0.05	-0.16	-0.41	-0.32	-0.35					
Education	0.16	0.22	0.05	0.02	-0.06	0.04	0.24	0.14	0.17	0.22	0.22	0.18	0.03	0.04	0.02	-0.12				
Age	0.19	0.23	0.07	0.1	-0.11	-0.08	0.18	0.13	0.28	0.27	0.18	0.18	0.2	0.16	0.2	-0.31	0.07			
Household income	0.15	0.2	0.06	0.05	-0.09	0.01	0.2	0.1	0.23	0.24	0.21	0.14	0.14	0.11	0.03	-0.22	0.41	0.15		
Personal income	0.12	0.18	0.02	0.02	-0.06	0.03	0.23	0.11	0.23	0.24	0.18	0.12	0.17	0.15	0.03	-0.26	0.43	0.21	0.85	
Female	-0.1	-0.16	0.01	-0.04	0.12	-0.11	-0.1	-0.07	-0.18	-0.18	-0.22	-0.07	0.05	0.03	0.05	0.22	-0.17	-0.1	-0.19	-0.22

Table 6: Correlations between measures of online hostility and self-reported measures in survey

	Political hate	Political	Hate	Toxicity score	Afinn score	Self-reported hostility	Offline participation	Online participation	Internal efficacy	Political interest	Political knowledge	Openness	Conscientiousness	Extraversion	Agreeableness	Neuroticism	Education	Age	Household income	Personal income
Political	0.91																			
Hate	0.79	0.5																		
Toxicity score	0.49	0.35	0.57																	
Afinn score	-0.44	-0.42	-0.37	-0.58																
Self-reported hostility	0.17	0.12	0.2	0.21	-0.16															
Offline participation	0.25	0.28	0.14	0.11	-0.17	0.33														
Online participation	0.33	0.32	0.26	0.28	-0.24	0.44	0.72													
Internal efficacy	0.28	0.3	0.17	0.21	-0.17	0.04	0.3	0.3												
Political interest	0.38	0.43	0.22	0.2	-0.2	0.11	0.45	0.47	0.49											
Political knowledge	0.3	0.33	0.18	0.14	-0.18	0	0.09	0.08	0.31	0.35										
Openness	0.11	0.13	0.06	0.07	-0.02	0.04	0.23	0.22	0.38	0.25	0.11									
Conscientiousness	-0.09	-0.06	-0.13	-0.09	0.07	-0.14	0.01	-0.06	0.22	0.02	-0.09	0.16								
Extraversion	-0.03	-0.03	-0.03	-0.03	-0.03	-0.01	0.2	0.1	0.14	0.04	-0.07	0.29	0.21							
Agreeableness	-0.04	-0.02	-0.05	-0.07	0.08	-0.18	0.05	-0.01	0.11	0.05	0	0.22	0.34	0.18						
Neuroticism	0.03	-0.02	0.11	0.13	-0.08	0.13	-0.12	0	-0.2	-0.06	-0.03	-0.19	-0.34	-0.28	-0.35					
Education	0.12	0.19	0	-0.02	-0.04	0.01	0.22	0.09	0.16	0.2	0.2	0.14	-0.01	0.04	-0.01	-0.1				
Age	0.15	0.2	0.01	0.06	-0.11	-0.09	0.21	0.12	0.21	0.23	0.17	0.21	0.16	0.14	0.17	-0.27	0.1			
Household income	0.14	0.18	0.04	0.04	-0.1	-0.02	0.17	0.05	0.2	0.2	0.23	0.13	0.07	0.07	0	-0.19	0.4	0.16		
Personal income	0.11	0.17	-0.01	0	-0.07	0	0.21	0.07	0.19	0.2	0.19	0.11	0.11	0.12	-0.01	-0.23	0.44	0.23	0.83	
Female	-0.09	-0.16	0.03	0.04	0.07	-0.07	-0.07	-0.02	-0.14	-0.15	-0.21	-0.07	0.11	0.06	0.07	0.14	-0.16	-0.02	-0.17	-0.18

Table 7: Correlations between measures of online hostility and self-reported measures in survey for those self-identifying as Democrats

	Political hate	Political	Hate	Toxicity score	Afinn score	Self-reported hostility	Offline participation	Online participation	Internal efficacy	Political interest	Political knowledge	Openness	Conscientiousness	Extraversion	Agreeableness	Neuroticism	Education	Age	Household income	Personal income
Political	0.93																			
Hate	0.87	0.65																		
Toxicity score	0.6	0.5	0.61																	
Afinn score	-0.57	-0.59	-0.46	-0.67																
Self-reported hostility	0.24	0.2	0.24	0.32	-0.2															
Offline participation	0.27	0.32	0.15	0.28	-0.23	0.33														
Online participation	0.37	0.38	0.27	0.41	-0.33	0.42	0.71													
Internal efficacy	0.26	0.28	0.15	0.25	-0.26	0.12	0.45	0.41												
Political interest	0.24	0.29	0.11	0.25	-0.19	0.11	0.5	0.46	0.55											
Political knowledge	0.18	0.2	0.11	0.14	-0.12	0.01	0.19	0.1	0.31	0.19										
Openness	0.05	0.08	-0.02	0.09	-0.02	0.02	0.32	0.23	0.3	0.28	0.19									
Conscientiousness	0	0.02	-0.03	-0.06	0.01	-0.2	0.12	0.06	0.26	0.14	0.01	0.2								
Extraversion	0.05	0.05	0.02	0.03	0	-0.07	0.25	0.17	0.3	0.2	0.05	0.36	0.31							
Agreeableness	-0.11	-0.09	-0.14	-0.11	0.16	-0.25	0.04	0.01	0.1	0.05	0.05	0.2	0.41	0.2						
Neuroticism	-0.13	-0.19	-0.01	-0.11	0.14	0.08	-0.18	-0.11	-0.39	-0.22	-0.26	-0.22	-0.44	-0.34	-0.33					
Education	0.16	0.2	0.07	0.06	-0.08	0.12	0.25	0.15	0.17	0.17	0.26	0.14	0.11	0.05	0.02	-0.18				
Age	0.3	0.32	0.17	0.23	-0.19	-0.03	0.21	0.18	0.38	0.34	0.26	0.13	0.16	0.21	0.15	-0.3	0.04			
Household income	0.15	0.19	0.07	0.07	-0.09	0.09	0.27	0.14	0.21	0.26	0.23	0.09	0.2	0.12	-0.01	-0.25	0.42	0.07		
Personal income	0.12	0.15	0.04	0.1	-0.1	0.1	0.29	0.16	0.23	0.27	0.23	0.11	0.23	0.16	0.02	-0.3	0.42	0.11	0.86	
Female	-0.1	-0.14	-0.03	-0.17	0.22	-0.19	-0.13	-0.13	-0.24	-0.19	-0.35	-0.07	0.08	0.04	0.08	0.25	-0.2	-0.18	-0.19	-0.21

Table 8: Correlations between measures of online hostility and self-reported measures in survey for those self-identifying as Republican

2.8 Additional results and outputs

In this section, we provide the full statistical models and diagnostics for key analyses in the main text as well as descriptive statistics for the key measures.

	Political hostility						Afinn						Toxicity					
Construct	Model 1	Model 2	Model 3	Model 4	Model 5	Model 6	Model 7	Model 8	Model 9	Model 10	Model 11	Model 12	Model 13	Model 14	Model 15	Model 16	Model 17	Model 18
Age	0.139	0.095	0.083	0.135	0.137	0.088	0.114	0.080	0.083	0.111	0.112	0.077	0.161	0.123	0.121	0.160	0.163	0.132
	(0.018)**	(0.018)**	(0.018)**	(0.017)**	(0.018)**	(0.020)**	(0.024)**	(0.024)**	(0.024)**	(0.024)**	(0.024)**	(0.026)**	(0.023)**	(0.025)**	(0.024)**	(0.023)**	(0.023)**	(0.024)**
Household Income	0.074	0.080	0.050	0.099	0.092	0.053	0.051	0.050	0.038	0.065	0.060	0.032	0.049	0.023	0.004	0.041	0.034	0.013
	(0.019)**	(0.017)**	(0.018)**	(0.018)**	(0.019)**	(0.019)**	(0.026)*	(0.023)*	(0.024)	(0.023)**	(0.023)**	(0.028)	(0.026)	(0.024)	(0.024)	(0.023)	(0.024)	(0.027)
Female	-0.059	-0.035	-0.025	-0.069	-0.065	-0.011	-0.054	-0.034	-0.035	-0.061	-0.057	-0.019	-0.035	-0.007	-0.004	-0.041	-0.035	-0.002
	(0.018)**	(0.018)	(0.018)	(0.017)**	(0.018)**	(0.021)	(0.032)	(0.034)	(0.030)	(0.033)	(0.033)	(0.029)	(0.024)	(0.027)	(0.023)	(0.025)	(0.025)	(0.024)
Party Identification	0.076	0.059	0.037	0.050	0.069	0.063	0.080	0.066	0.058	0.060	0.074	0.069	0.088	0.066	0.052	0.052	0.074	0.075
	(0.021)**	(0.021)**	(0.020)	(0.021)*	(0.021)**	(0.022)**	(0.025)**	(0.026)*	(0.024)*	(0.025)*	(0.026)**	(0.025)**	(0.024)**	(0.026)**	(0.024)*	(0.024)*	(0.025)**	(0.024)**
Openness	0.057	0.017	0.003	0.021	0.058	0.038	-0.083	-0.116	-0.113	-0.112	-0.084	-0.098	-0.114	-0.164	-0.166	-0.169	-0.125	-0.134
	(0.021)**	(0.021)	(0.025)	(0.022)	(0.022)**	(0.021)	(0.075)	(0.077)	(0.083)	(0.079)	(0.075)	(0.078)	(0.038)**	(0.037)**	(0.040)**	(0.036)**	(0.037)**	(0.034)**
Conscientiousness	-0.075	-0.103	-0.047	-0.064	-0.070	-0.071	-0.223	-0.244	-0.208	-0.215	-0.220	-0.220	-0.075	-0.099	-0.052	-0.060	-0.067	-0.070
	(0.022)**	(0.021)**	(0.026)	(0.023)**	(0.023)**	(0.026)**	(0.085)**	(0.086)**	(0.083)*	(0.083)**	(0.085)**	(0.079)**	(0.093)	(0.094)	(0.089)	(0.091)	(0.094)	(0.086)
Extraversion	0.015	0.012	0.034	0.001	0.006	0.011	0.126	0.124	0.137	0.116	0.120	0.124	0.113	0.113	0.131	0.100	0.107	0.113
	(0.021)	(0.020)	(0.022)	(0.025)	(0.022)	(0.020)	(0.064)*	(0.064)	(0.063)*	(0.059)*	(0.062)	(0.065)	(0.041)**	(0.041)**	(0.040)**	(0.033)**	(0.038)**	(0.042)**
Agreeableness	-0.048	-0.042	-0.125	-0.029	-0.039	-0.109	-0.047	-0.042	-0.089	-0.031	-0.039	-0.090	-0.029	-0.022	-0.088	-0.004	-0.017	-0.065
	(0.023)*	(0.023)	(0.026)**	(0.031)	(0.027)	(0.030)**	(0.033)	(0.034)	(0.037)*	(0.037)	(0.037)	(0.035)**	(0.036)	(0.037)	(0.042)*	(0.030)	(0.034)	(0.037)
Neuroticism	0.005	0.025	0.009	0.054	0.017	-0.067	-0.035	-0.019	-0.034	0.002	-0.027	-0.087	0.050	0.074	0.057	0.111	0.068	0.009
	(0.021)	(0.020)	(0.023)	(0.023)*	(0.022)	(0.035)	(0.048)	(0.046)	(0.046)	(0.053)	(0.051)	(0.040)*	(0.043)	(0.043)	(0.041)	(0.049)*	(0.048)	(0.039)
Education	0.072						0.041						-0.015					
	(0.019)**						(0.029)						(0.025)					
Internal Efficacy		0.226						0.175						0.212				
		(0.019)**						(0.062)**						(0.051)**				
Political Interest			0.306						0.170						0.239			
			(0.030)**						(0.043)**						(0.035)**			
Political Knowledge						0.233						0.169						0.145
						(0.038)**						(0.041)**						(0.039)**
Online Participation				0.236						0.176						0.267		
				(0.025)**						(0.037)**						(0.028)**		
Offline Participation					0.094						0.065						0.094	
					(0.032)**						(0.042)						(0.030)**	
Intercept	4.267	3.911	4.212	4.118	4.264	4.220	6.662	6.360	6.640	6.529	6.648	6.610	1.649	1.174	1.489	1.314	1.492	1.516
	(0.167)**	(0.166)**	(0.151)**	(0.183)**	(0.172)**	(0.150)**	(0.346)**	(0.303)**	(0.347)**	(0.377)**	(0.357)**	(0.331)**	(0.402)**	(0.409)**	(0.375)**	(0.448)**	(0.435)**	(0.364)**
N	9871	9871	9871	9871	9871	9871	9871	9871	9871	9871	9871	9871	9871	9871	9871	9871	9871	9871

Table 9: Regression table for models for figure **??**

*The table illustrates the regression output used to create figure **??** in the main text, for the three dependent variables Political Hostility, Afinn scores and Toxicity scores. Standard errors are in parentheses. # indicates significant at .1, * indicates significant at .05 and ** indicates significant at .01

Construct	Political hostility						Afinn						Toxicity					
	Model 1	Model 2	Model 3	Model 4	Model 5	Model 6	Model 7	Model 8	Model 9	Model 10	Model 11	Model 12	Model 13	Model 14	Model 15	Model 16	Model 17	Model 18
Age	0.048 (0.023)*	0.019 (0.023)	0.002 (0.022)	0.024 (0.023)	0.023 (0.024)	0.030 (0.023)	0.005 (0.024)	0.003 (0.024)	-0.019 (0.025)	-0.018 (0.024)	-0.016 (0.024)	-0.008 (0.024)	0.036 (0.025)	0.027 (0.025)	-0.005 (0.026)	0.009 (0.025)	0.019 (0.026)	0.021 (0.025)
Female	-0.058 (0.021)**	-0.039 (0.021)#	-0.029 (0.020)	-0.067 (0.020)**	-0.065 (0.021)**	-0.033 (0.021)	-0.059 (0.024)*	-0.070 (0.023)**	-0.050 (0.023)*	-0.067 (0.023)**	-0.066 (0.023)**	-0.035 (0.024)	-0.012 (0.025)	-0.009 (0.025)	0.017 (0.025)	-0.005 (0.024)	-0.008 (0.025)	0.014 (0.026)
Affective Polarization	0.213 (0.026)**	0.128 (0.029)**	0.124 (0.029)**	0.175 (0.027)**	0.202 (0.027)**	0.147 (0.030)**	0.111 (0.032)**	0.148 (0.028)**	0.072 (0.036)*	0.076 (0.034)*	0.097 (0.032)**	0.041 (0.035)	0.164 (0.029)**	0.192 (0.025)**	0.096 (0.032)**	0.116 (0.030)**	0.151 (0.030)**	0.124 (0.033)**
Education	0.139 (0.022)**						0.103 (0.024)**						0.013 (0.025)					
Internal Efficacy		0.214 (0.031)**						0.039 (0.009)**						0.047 (0.009)**				
Political Interest			0.297 (0.032)**						0.167 (0.037)**						0.224 (0.032)**			
Political Knowledge						0.239 (0.039)**						0.213 (0.045)**						0.176 (0.040)**
Online Participation				0.279 (0.032)**						0.250 (0.033)**						0.289 (0.030)**		
Offline Participation					0.253 (0.046)**						0.233 (0.042)**						0.168 (0.041)**	
Affective Polarization x Education	-0.005 (0.028)						0.011 (0.031)						0.026 (0.028)					
Affective Polarization x Internal Efficacy		-0.061 (0.034)#						0.159 (0.013)**						0.149 (0.014)**				
Affective Polarization x Political Interest			0.034 (0.034)						0.076 (0.041)#						0.045 (0.039)			
Affective Polarization x Political Knowledge						0.028 (0.040)						-0.007 (0.055)						0.060 (0.039)
Affective Polarization x Online Participation				0.154 (0.036)**						0.177 (0.054)**						0.123 (0.036)**		
Affective Polarization x Offline Participation					0.202 (0.045)**						0.211 (0.058)**						0.112 (0.040)**	
Intercept	0.063 (0.027)*	0.409 (0.021)**	0.399 (0.026)**	0.180 (0.036)**	0.062 (0.048)	0.367 (0.035)**	0.060 (0.026)*	0.409 (0.022)**	0.390 (0.025)**	0.172 (0.035)**	0.054 (0.046)	0.357 (0.035)**	0.061 (0.027)*	0.408 (0.023)**	0.390 (0.027)**	0.178 (0.034)**	0.059 (0.046)	0.356 (0.036)**

Table 10: Regression table for models for figure 3 in appendix 2

*The table illustrates the regression output used to create figure 3 in appendix 2, for the three dependent variables Political Hostility, Afinn scores and Toxicity scores. Standard errors are in parentheses. # indicates significant at .1, * indicates significant at .05 and ** indicates significant at .01

Model	LL	BIC
Poisson regression	-562743.898	1125504.106
Negative Binomial Regression	-21443.396	42911.255
Zero Inflated Negative Binomial	-21443.396	42927.564

Table 11: Fit statistics for count models for number of followers

Model	LL	BIC
Poisson regression	-782805.547	1565627.38
Negative Binomial Regression	-24141.228	48306.884
Zero Inflated Negative Binomial	-24123.117	48286.948

Table 12: Fit statistics for count models for number of friends

Model	LL	BIC
Poisson regression	-552085.897	1104186.823
Negative Binomial Regression	-12024.56	24071.665
Zero Inflated Negative Binomial	-12024.561	24086.696

Table 13: Fit statistics for count models for number of tweets

Construct	Wave1	Wave2	Wave3	Wave4	Wave5	Wave6
Female	yes	yes	yes	yes	yes	yes
Age	yes	yes	yes	yes	yes	yes
Education	yes	yes	yes	yes	yes	yes
Household Income	yes	yes	yes	yes	yes	yes
Internal efficacy	no	no	no	yes	yes	yes
Party identification	yes	yes	yes	yes	yes	yes
Political interest	yes	no	no	no	no	no
Online participation	yes	no	no	no	no	no
Affective polarization	no	no	yes	yes	yes	yes
Openness	no	no	no	no	yes	yes
Conscientiousness	no	no	no	no	yes	yes
Extraversion	no	no	no	no	yes	yes
Agreeableness	no	no	yes	yes	yes	yes
Neuroticism	no	no	yes	yes	yes	yes

Table 14: Constructs available in different waves

Construct	1	2	3	4	5	6
Affective polarization$_{max}$	1	1	1	1	1	1
Affective polarization$_{mean}$	0.668	0.669	0.674	0.677	0.678	0.679
Affective polarization$_{min}$	0	0	0	0	0	0
Affective polarization$_{sd}$	0.225	0.224	0.222	0.227	0.227	0.227
Affective polarization$_{count}$	1403	1131	1195	1449	1413	1267
Age$_{max}$	1	1	1	1	1	1
Age$_{mean}$	0.347	0.411	0.426	0.423	0.437	0.448
Age$_{min}$	0	0	0	0	0	0
Age$_{sd}$	0.224	0.219	0.218	0.221	0.219	0.216
Age$_{count}$	2212	1446	1406	1695	1643	1469
Agreeableness$_{max}$			1	1	1	1
Agreeableness$_{mean}$			0.671	0.681	0.682	0.684
Agreeableness$_{min}$			0	0.111	0	0.074
Agreeableness$_{sd}$			0.143	0.144	0.15	0.145
Agreeableness$_{count}$	0	0	1406	1695	1643	1469
Conscientiousness$_{max}$					1	1
Conscientiousness$_{mean}$					0.667	0.668
Conscientiousness$_{min}$					0.029	0
Conscientiousness$_{sd}$					0.187	0.192
Conscientiousness$_{count}$	0	0	0	0	1643	1469
Education$_{max}$	1	1	1	1	1	1
Education$_{mean}$	0.658	0.676	0.686	0.695	0.692	0.704
Education$_{min}$	0	0	0	0	0	0
Education$_{sd}$	0.274	0.27	0.27	0.265	0.266	0.257
Education$_{count}$	2212	1446	1406	1695	1643	1469

Table 15: Descriptive statistics for constructs

Construct	1	2	3	4	5	6
Extraversion$_{max}$					1	1
Extraversion$_{mean}$					0.492	0.491
Extraversion$_{min}$					0	0
Extraversion$_{sd}$					0.225	0.223
Extraversion$_{count}$	0	0	0	0	1643	1469
Female$_{max}$	1	1	1	1	1	1
Female$_{mean}$	0.499	0.486	0.494	0.483	0.481	0.474
Female$_{min}$	0	0	0	0	0	0
Female$_{sd}$	0.5	0.5	0.5	0.5	0.5	0.5
Female$_{count}$	2212	1446	1406	1695	1643	1469
Hate$_{max}$	0.851	0.801	0.734	1	0.852	0.849
Hate$_{mean}$	0.482	0.475	0.479	0.493	0.491	0.506
Hate$_{min}$	0.122	0	0.152	0.135	0.19	0.191
Hate$_{sd}$	0.09	0.09	0.083	0.093	0.081	0.083
Hate$_{count}$	1443	934	796	972	916	700
Household income$_{max}$	1	1	1	1	1	1
Household income$_{mean}$	0.384	0.399	0.409	0.418	0.416	0.418
Household income$_{min}$	0	0	0	0	0	0
Household income$_{sd}$	0.234	0.233	0.233	0.234	0.234	0.237
Household income$_{count}$	2071	1366	1327	1609	1553	1389

Table 16: Descriptive statistics for constructs continued

Construct	1	2	3	4	5	6
Internal efficacy$_{max}$				1	1	1
Internal efficacy$_{mean}$				0.768	0.769	0.768
Internal efficacy$_{min}$				0.056	0	0
Internal efficacy$_{sd}$				0.207	0.204	0.21
Internal efficacy$_{count}$	0	0	0	1695	1643	1469
Neuroticism$_{max}$			1	1	1	1
Neuroticism$_{mean}$			0.436	0.451	0.445	0.433
Neuroticism$_{min}$			0	0	0	0
Neuroticism$_{sd}$			0.218	0.224	0.227	0.221
Neuroticism$_{count}$	0	0	1406	1695	1643	1469
Online participation$_{max}$	1					
Online participation$_{mean}$	0.233					
Online participation$_{min}$	0					
Online participation$_{sd}$	0.183					
Online participation$_{count}$	2212	0	0	0	0	0

Table 17: Descriptive statistics for constructs continued

Construct	1	2	3	4	5	6
Openness$_{max}$					1	1
Openness$_{mean}$					0.685	0.688
Openness$_{min}$					0.051	0
Openness$_{sd}$					0.168	0.168
Openness$_{count}$	0	0	0	0	1643	1469
Party identification$_{max}$	1	1	1	1	1	1
Party identification$_{mean}$	0.631	0.639	0.639	0.643	0.639	0.649
Party identification$_{min}$	0	0	0	0	0	0
Party identification$_{sd}$	0.353	0.359	0.36	0.359	0.362	0.351
Party identification$_{count}$	2212	1446	1406	1695	1643	1469
Politica hate$_{max}$	1	0.887	0.836	0.975	0.841	0.891
Politica hate$_{mean}$	0.513	0.516	0.517	0.535	0.533	0.551
Politica hate$_{min}$	0.082	0.115	0.103	0	0.123	0.115
Politica hate$_{sd}$	0.109	0.113	0.105	0.112	0.103	0.107
Politica hate$_{count}$	1443	934	796	972	916	700
Political interest$_{max}$	1					
Political interest$_{mean}$	0.746					
Political interest$_{min}$	0					
Political interest$_{sd}$	0.283					
Political interest$_{count}$	2212	0	0	0	0	0
Political$_{max}$	0.926	0.892	0.858	0.894	1	0.925
Political$_{mean}$	0.487	0.499	0.497	0.511	0.511	0.526
Political$_{min}$	0.094	0.079	0.045	0	0.094	0.135
Political$_{sd}$	0.107	0.116	0.108	0.109	0.111	0.106
Political$_{count}$	1443	934	796	972	916	700

Table 18: Descriptive statistics for constructs continued

Median tweets	Mean tweets	Min tweets	Max tweets	Sd tweets
845.0	2539.166	1.0	136146.0	6380.274

Table 19: Descriptive statistics for number of tweets in the panel

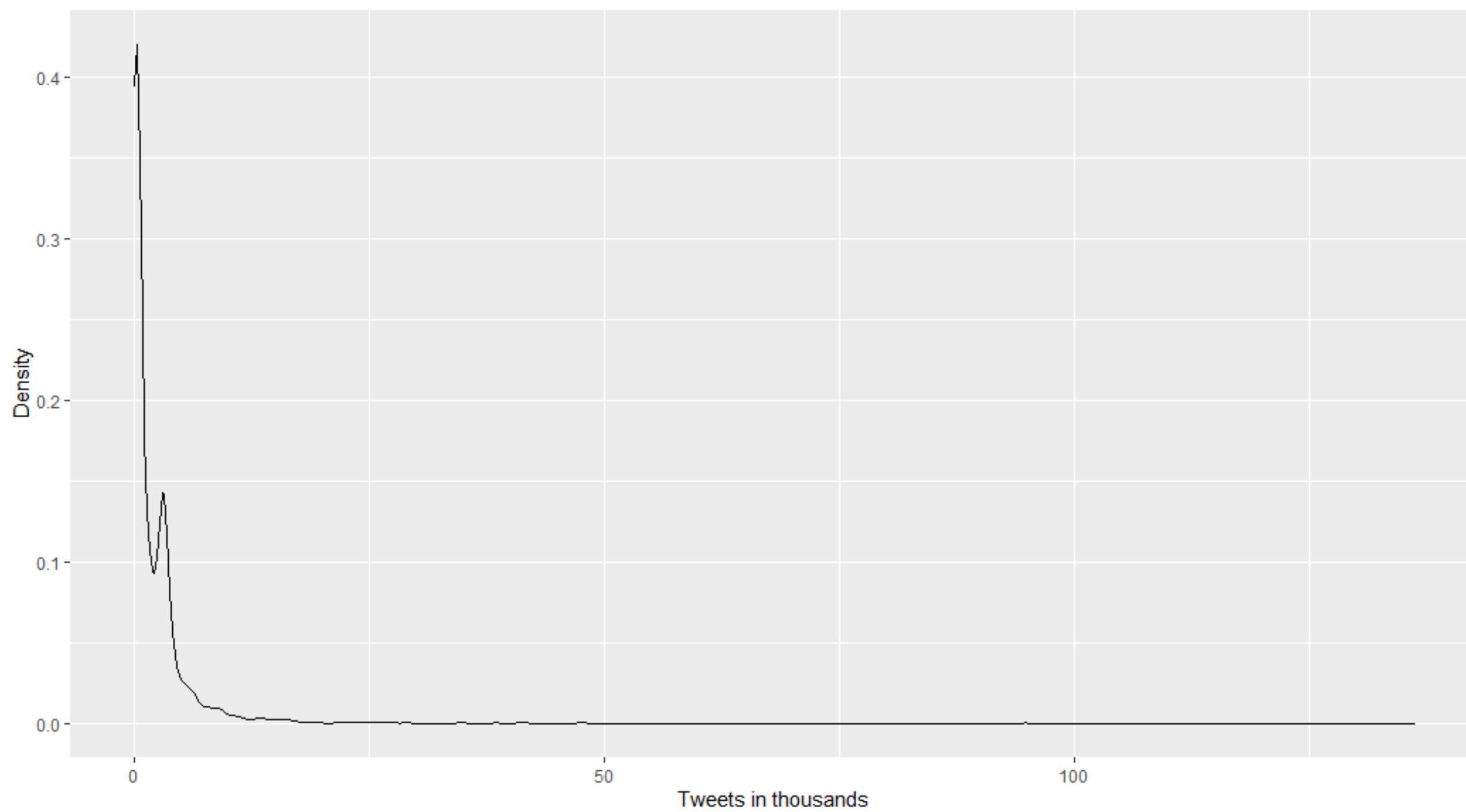

Figure 5: Density plot for number of tweets in the panel

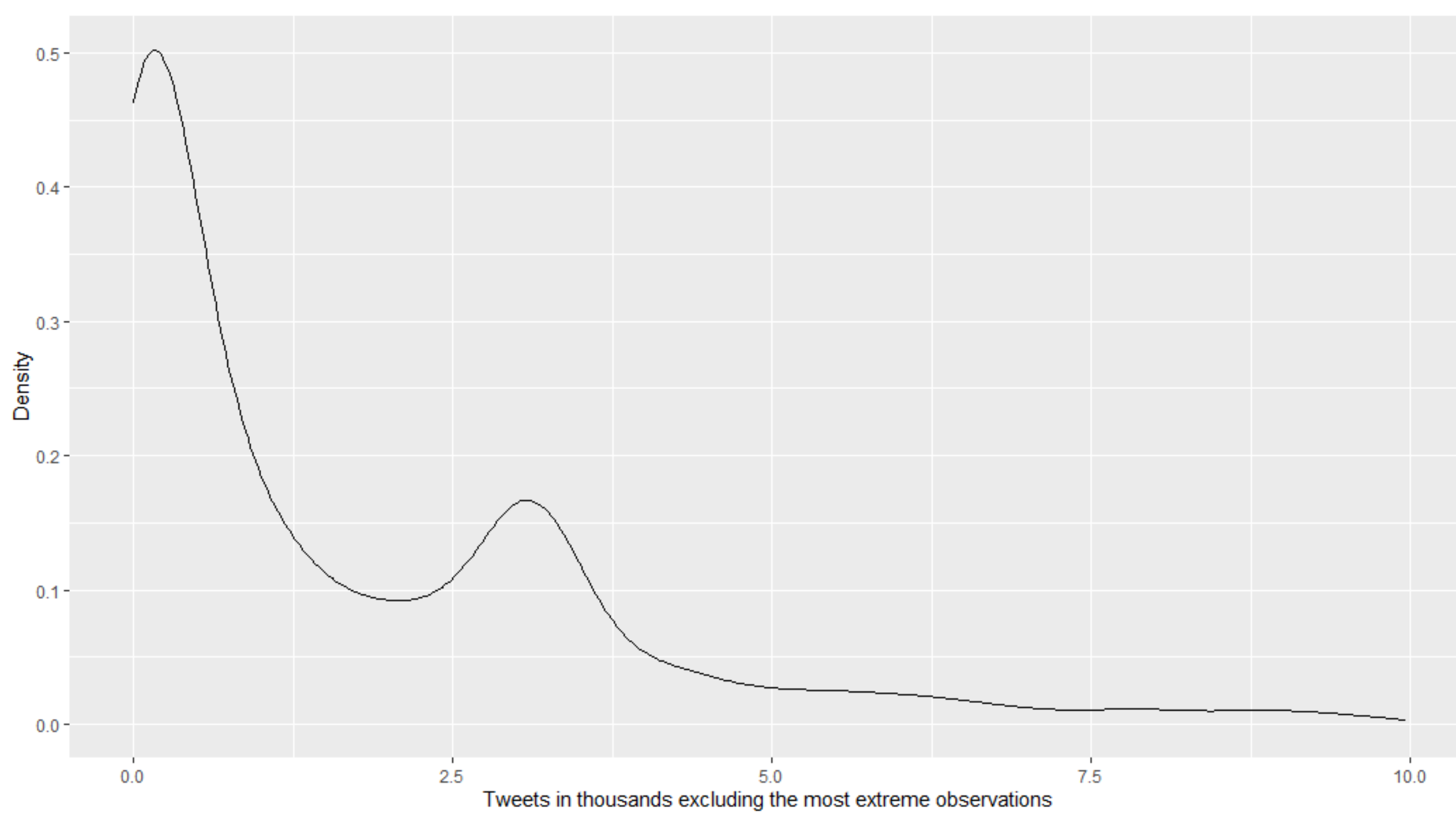

Figure 6: Density plot for number of tweets in the panel excluding those with more than 10,000 tweets

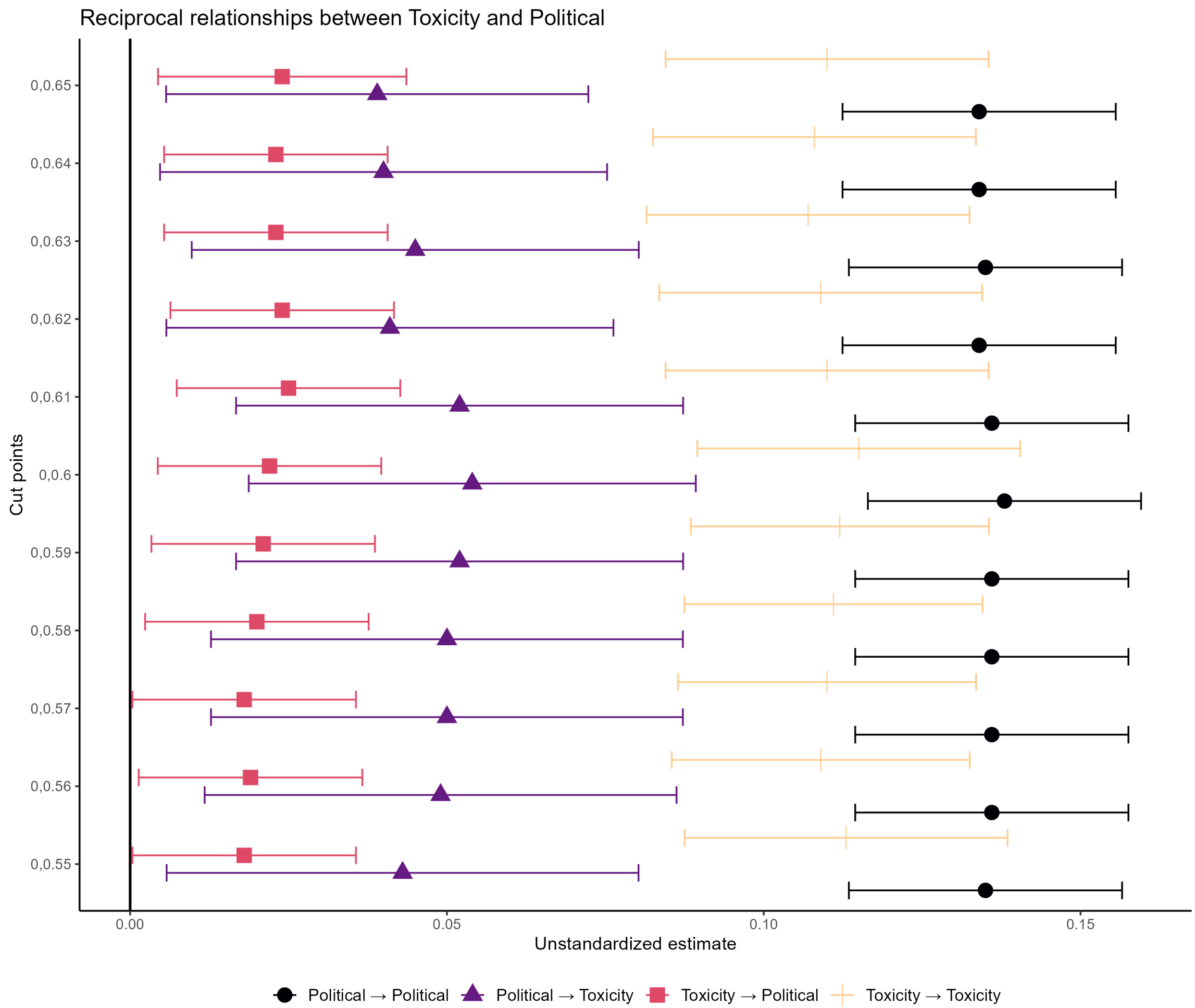

Figure 7: RI-CLPM for toxicity exploring the robustness of the cutpoints

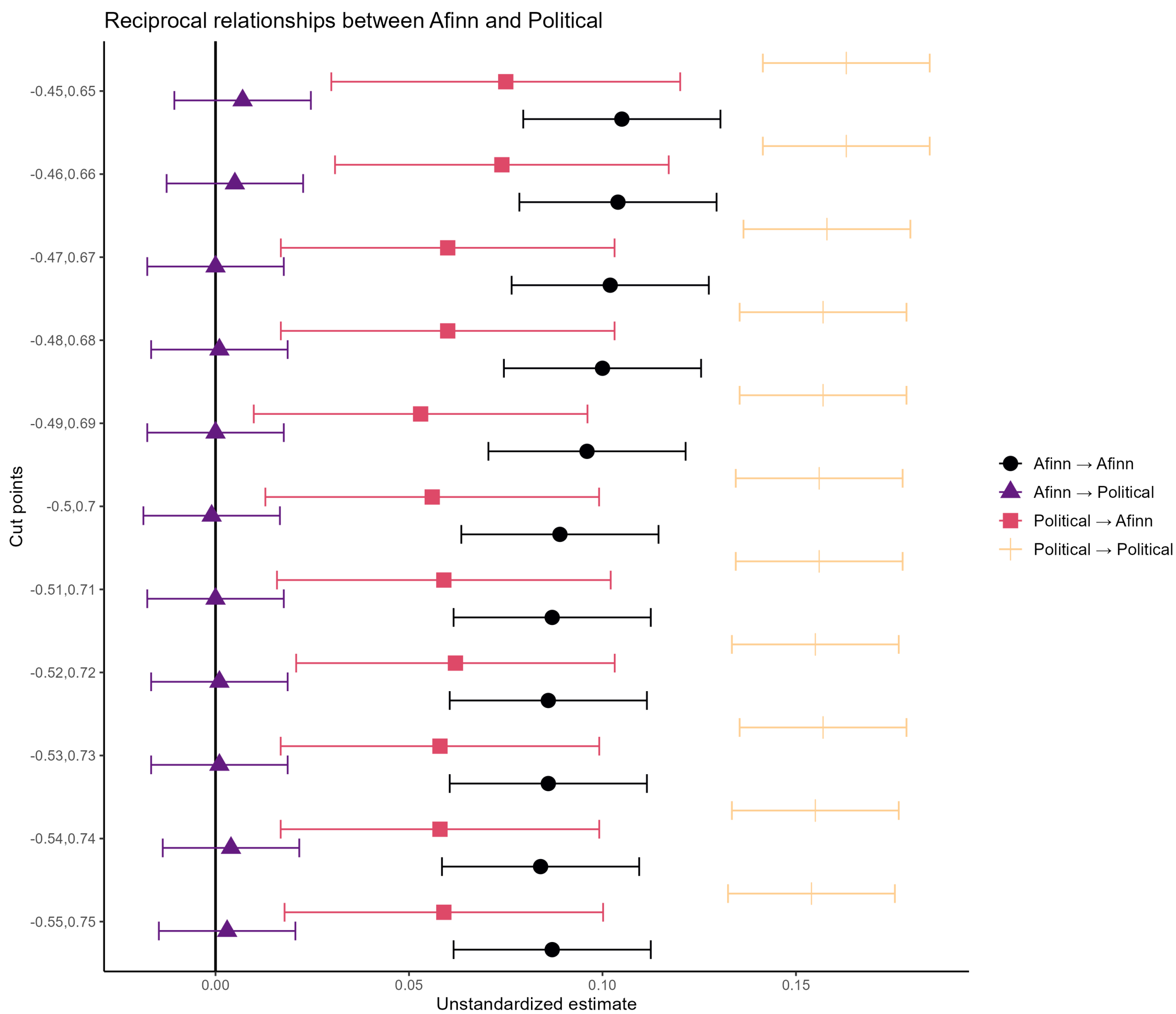

Figure 8: RI-CLPM for Afinn exploring the robustness of the cutpoints

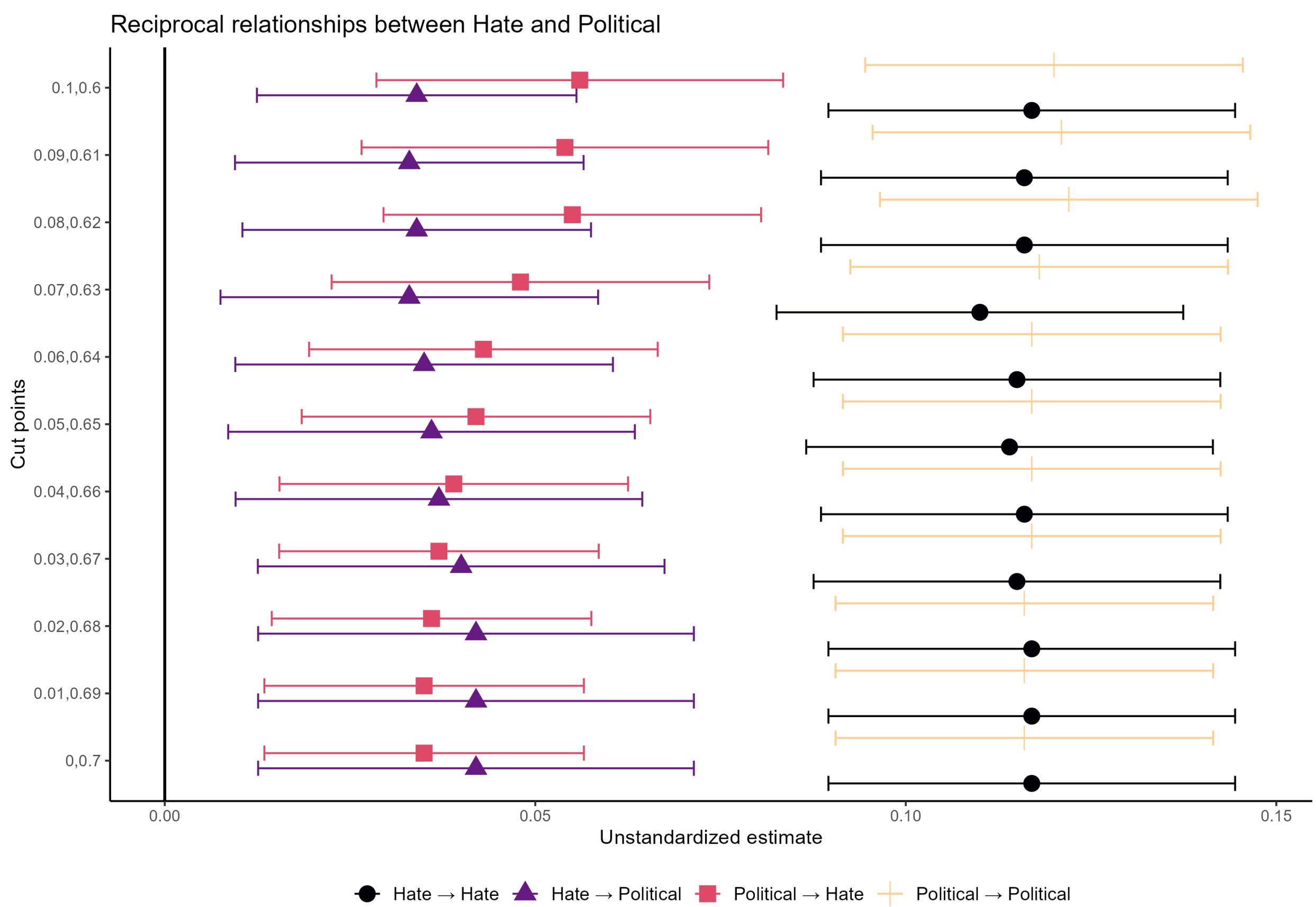

Figure 9: RI-CLPM for Hate exploring the robustness of the cutpoints

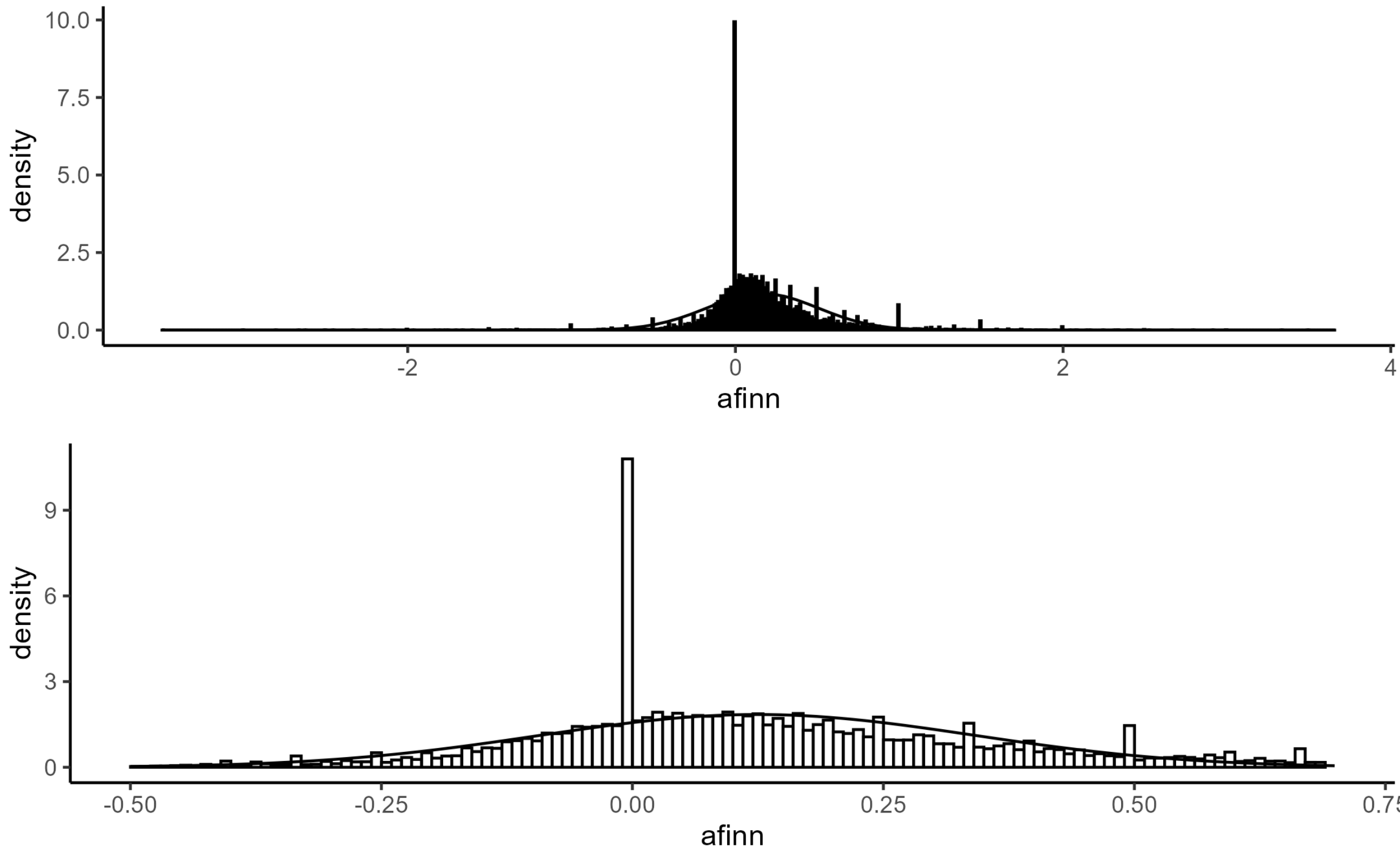

Figure 10: Distributions for Afinn before and after removing outliers

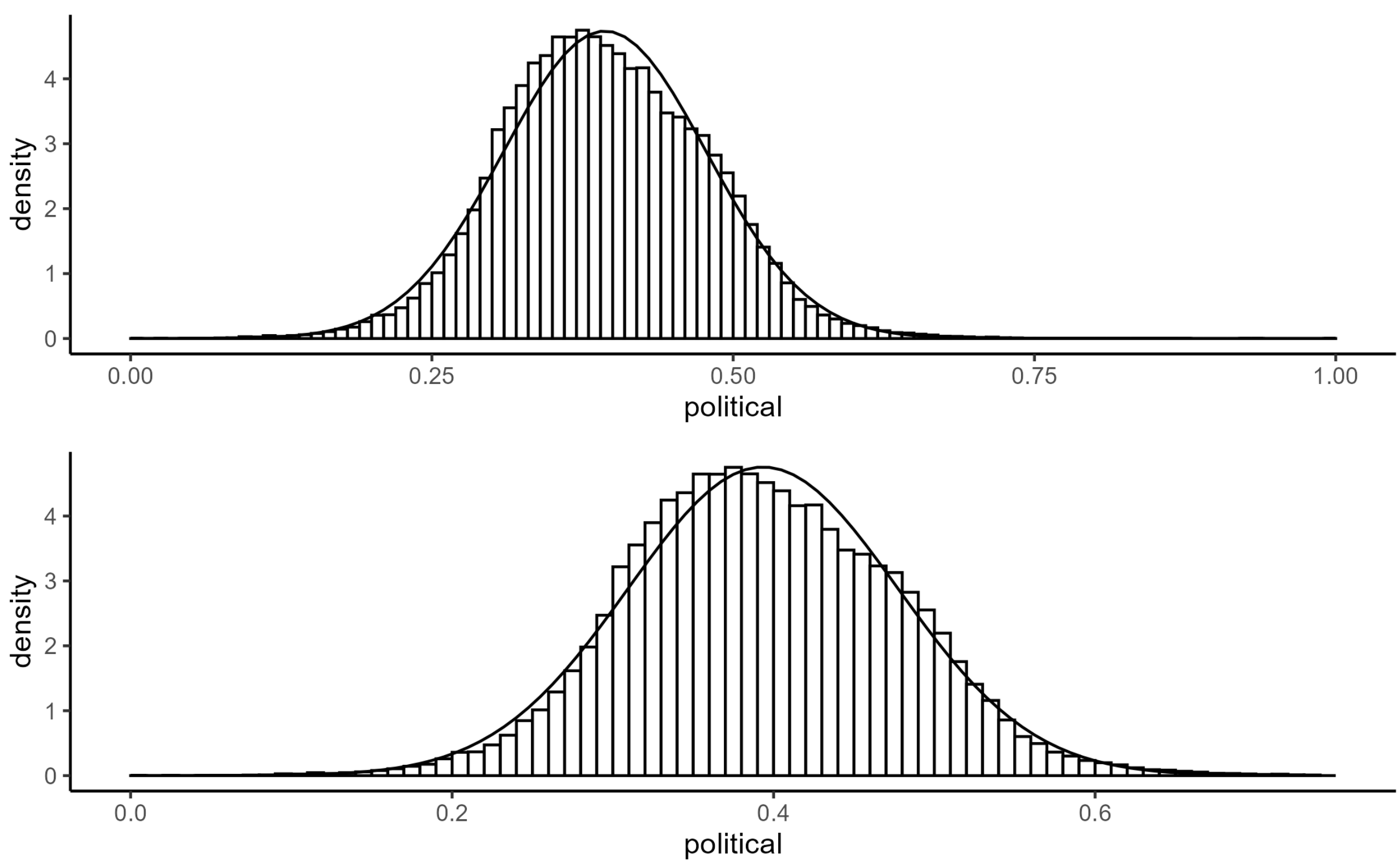

Figure 11: Distributions for political before and after removing outliers

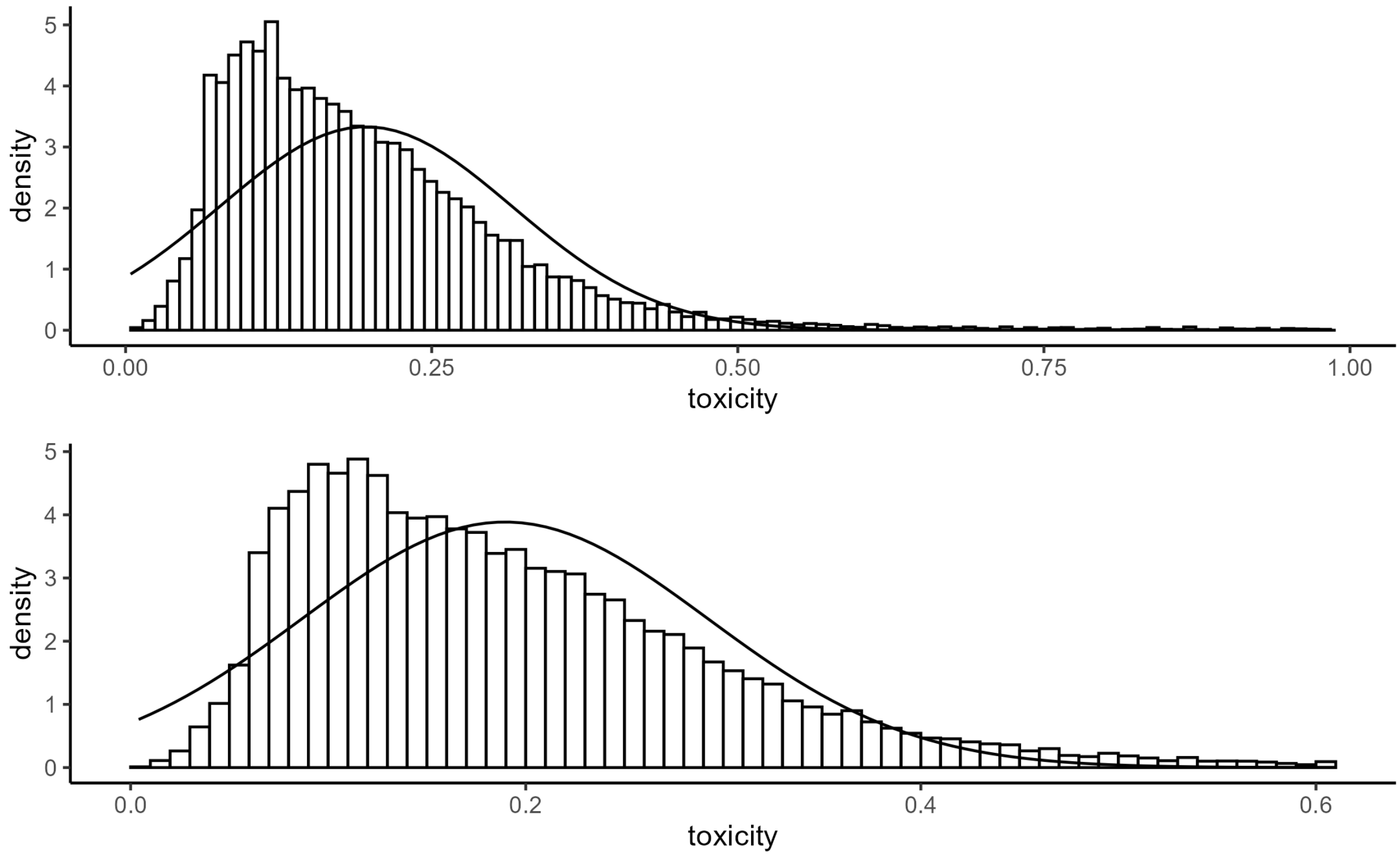

Figure 12: Distributions for toxicity before and after removing outliers

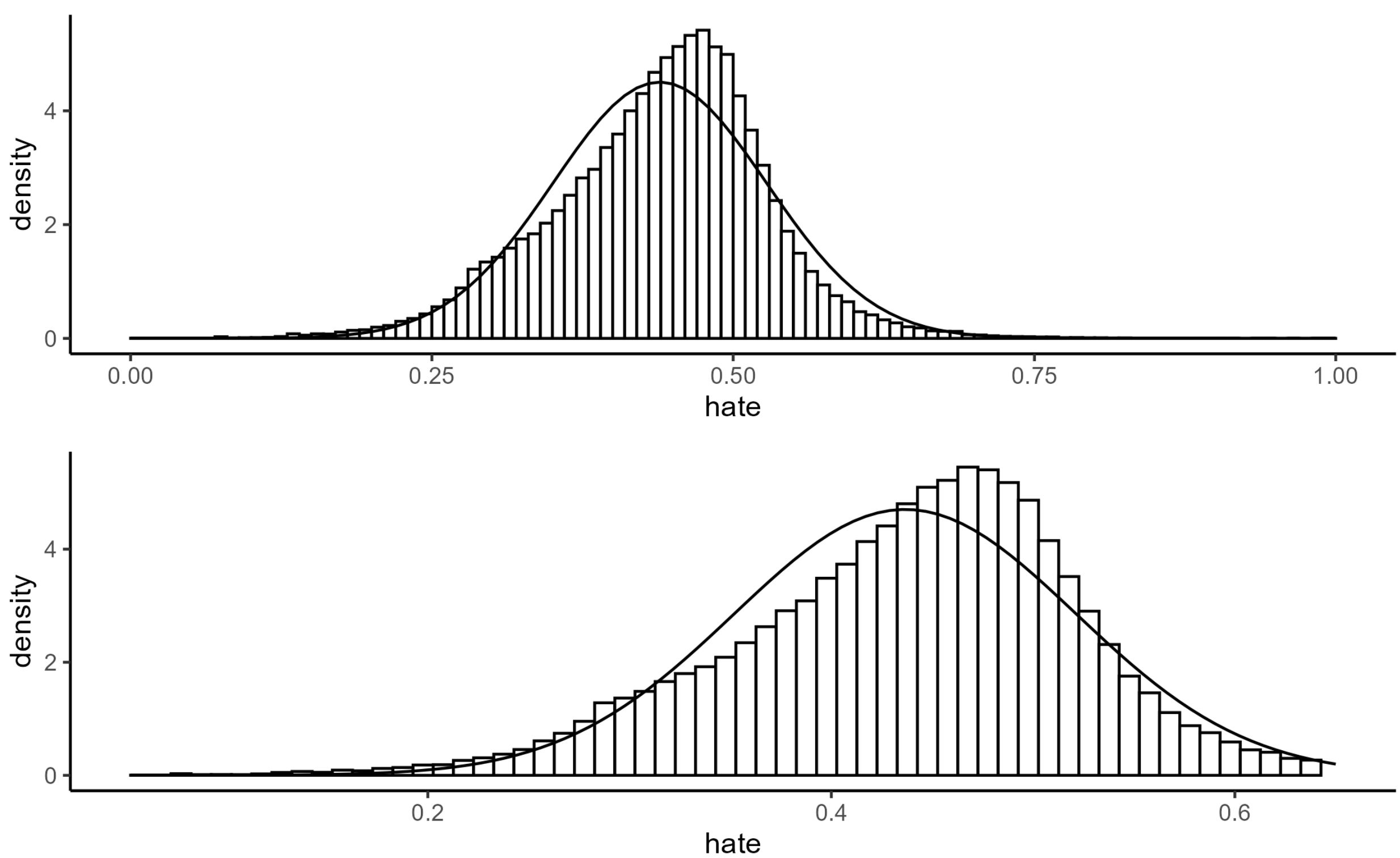

Figure 13: Distributions for hate before and after removing outliers

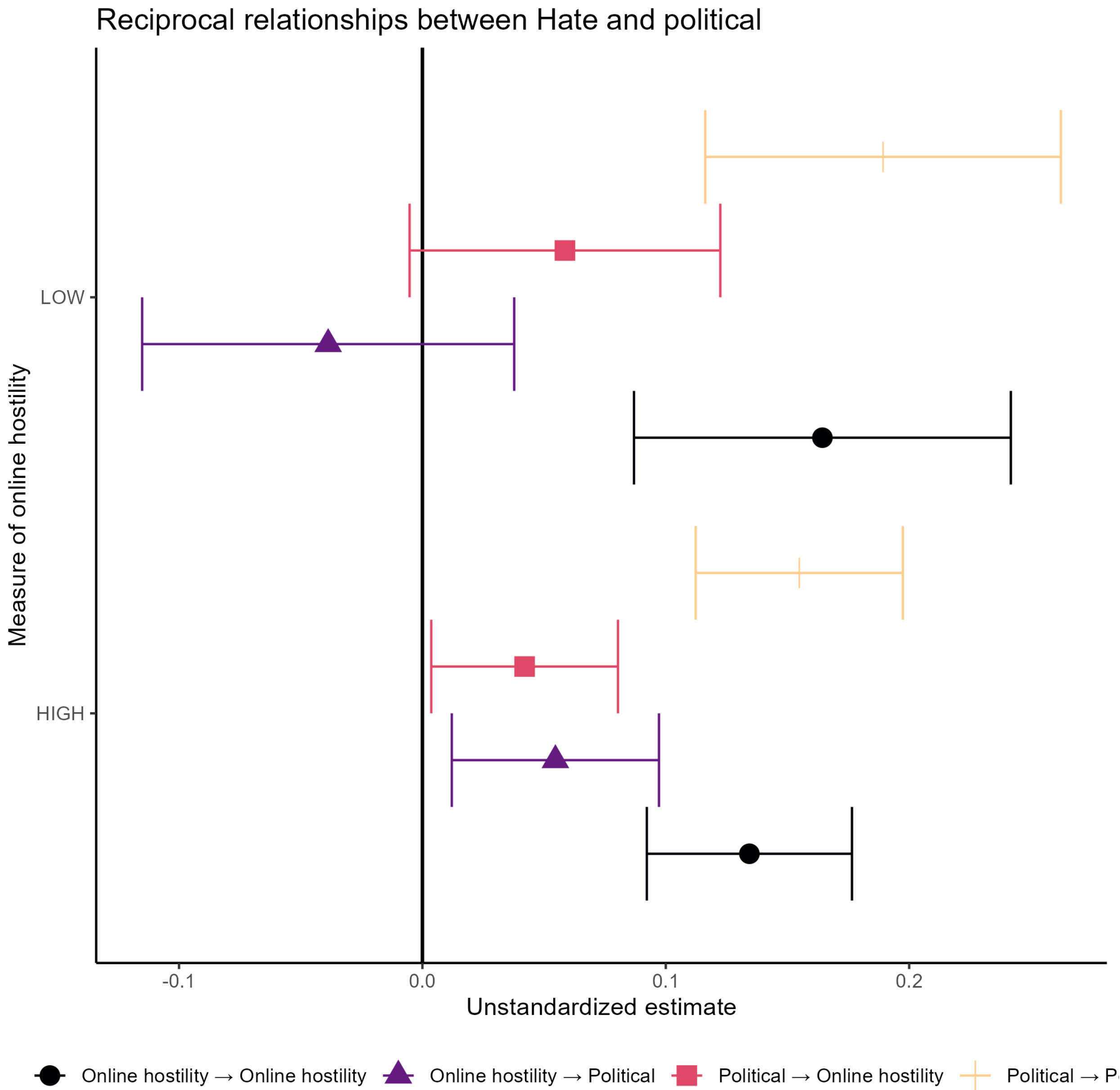

Figure 14: RI-CLPM estimates for the reciprocal effects of hate and political across those high and low on affective polarization.The results represent averages of models estimated using cutpoints in the range [0.5;0.65]. Full results can be seen in table 2.8

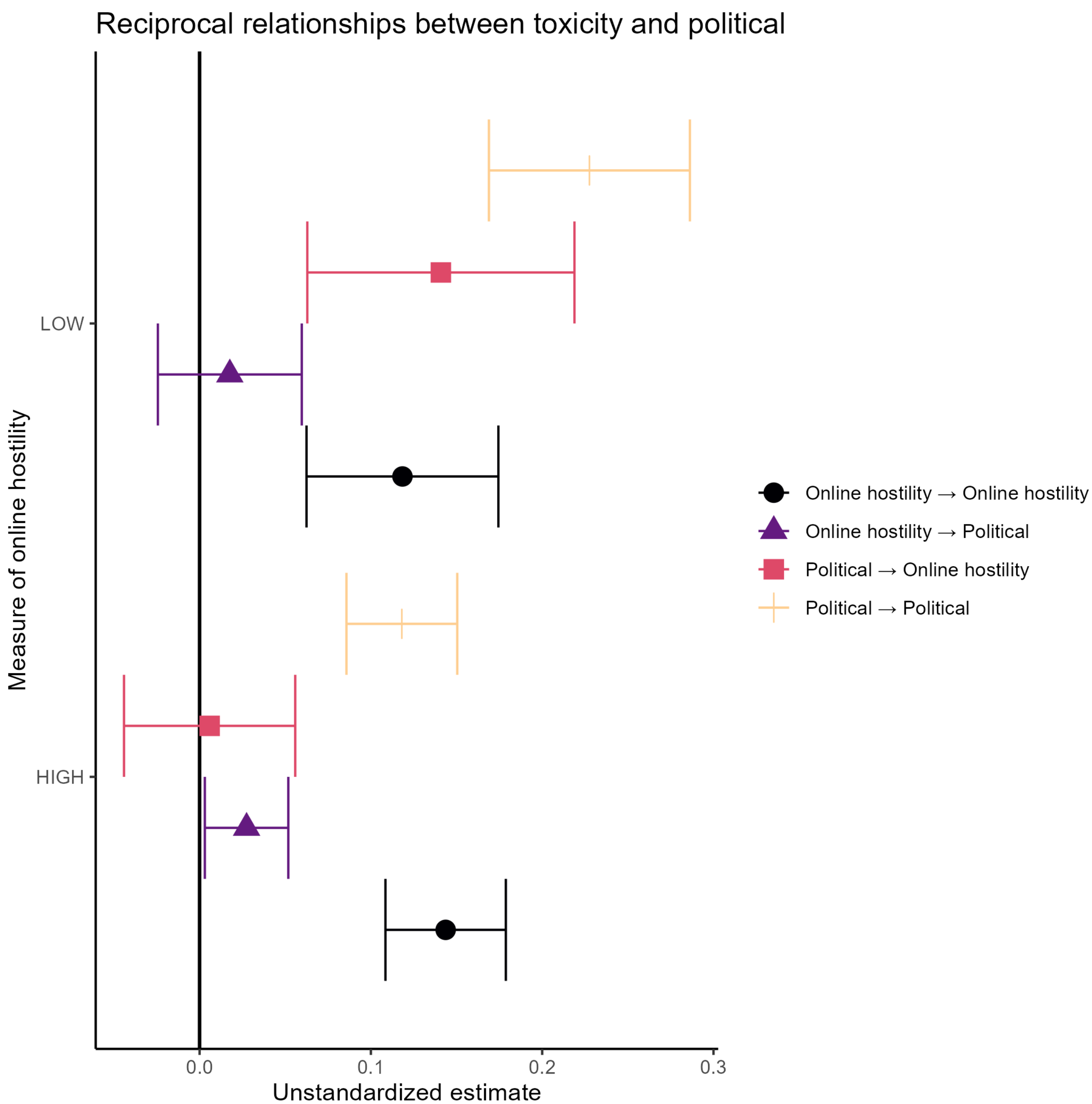

Figure 15: RI-CLPM estimates for the reciprocal effects of hate and toxicity across those high and low on affective polarization

The results represent averages of models estimated using cutpoints in the range [0.5;0.65]

Parameter	Estimate	Standard error	N
Online hostility → Online hostility	0.114	0.014	2377.0
Political → Online hostility	0.052	0.016	2377.0
Online hostility → Political	0.029	0.01	2377.0
Political → Political	0.112	0.013	2377.0
Online hostility → Online hostility	0.114	0.012	2812.0
Political → Online hostility	0.06	0.024	2812.0
Online hostility → Political	0.016	0.007	2812.0
Political → Political	0.124	0.011	2812.0
Online hostility → Online hostility	0.088	0.013	2785.0
Political → Online hostility	0.081	0.028	2785.0
Online hostility → Political	0.0	0.007	2785.0
Political → Political	0.141	0.011	2785.0

Group	Dependent variable	Parameter	5% CI	95 % CI	Estimate	N
HIGH	Afinn	Online hostility → Online hostility	0.09	0.16	0.12	1409.0
HIGH	Afinn	Online hostility → Political	-0.03	0.02	-0.0	1409.0
HIGH	Afinn	Political → Online hostility	0.01	0.12	0.07	1409.0
HIGH	Afinn	Political → Political	0.11	0.18	0.15	1409.0
HIGH	Political hate	Online hostility → Online hostility	0.09	0.18	0.13	1166.0
HIGH	Political hate	Online hostility → Political	0.01	0.1	0.05	1166.0
HIGH	Political hate	Political → Online hostility	0.0	0.08	0.04	1166.0
HIGH	Political hate	Political → Political	0.11	0.2	0.15	1166.0
HIGH	Toxicity	Online hostility → Online hostility	0.11	0.18	0.14	1424.0
HIGH	Toxicity	Online hostility → Political	0.0	0.05	0.03	1424.0
HIGH	Toxicity	Political → Online hostility	-0.04	0.06	0.01	1424.0
HIGH	Toxicity	Political → Political	0.09	0.15	0.12	1424.0
LOW	Afinn	Online hostility → Online hostility	-0.04	0.08	0.02	1409.0
LOW	Afinn	Online hostility → Political	-0.03	0.05	0.01	1409.0
LOW	Afinn	Political → Online hostility	-0.01	0.19	0.09	1409.0
LOW	Afinn	Political → Political	0.2	0.33	0.26	1409.0
LOW	Political hate	Online hostility → Online hostility	0.09	0.24	0.16	1166.0
LOW	Political hate	Online hostility → Political	-0.12	0.04	-0.04	1166.0
LOW	Political hate	Political → Online hostility	-0.01	0.12	0.06	1166.0
LOW	Political hate	Political → Political	0.12	0.26	0.19	1166.0
LOW	Toxicity	Online hostility → Online hostility	0.06	0.17	0.12	1424.0
LOW	Toxicity	Online hostility → Political	-0.02	0.06	0.02	1424.0
LOW	Toxicity	Political → Online hostility	0.06	0.22	0.14	1424.0
LOW	Toxicity	Political → Political	0.17	0.29	0.23	1424.0

Table 20: Output for figures 20 and 14-15

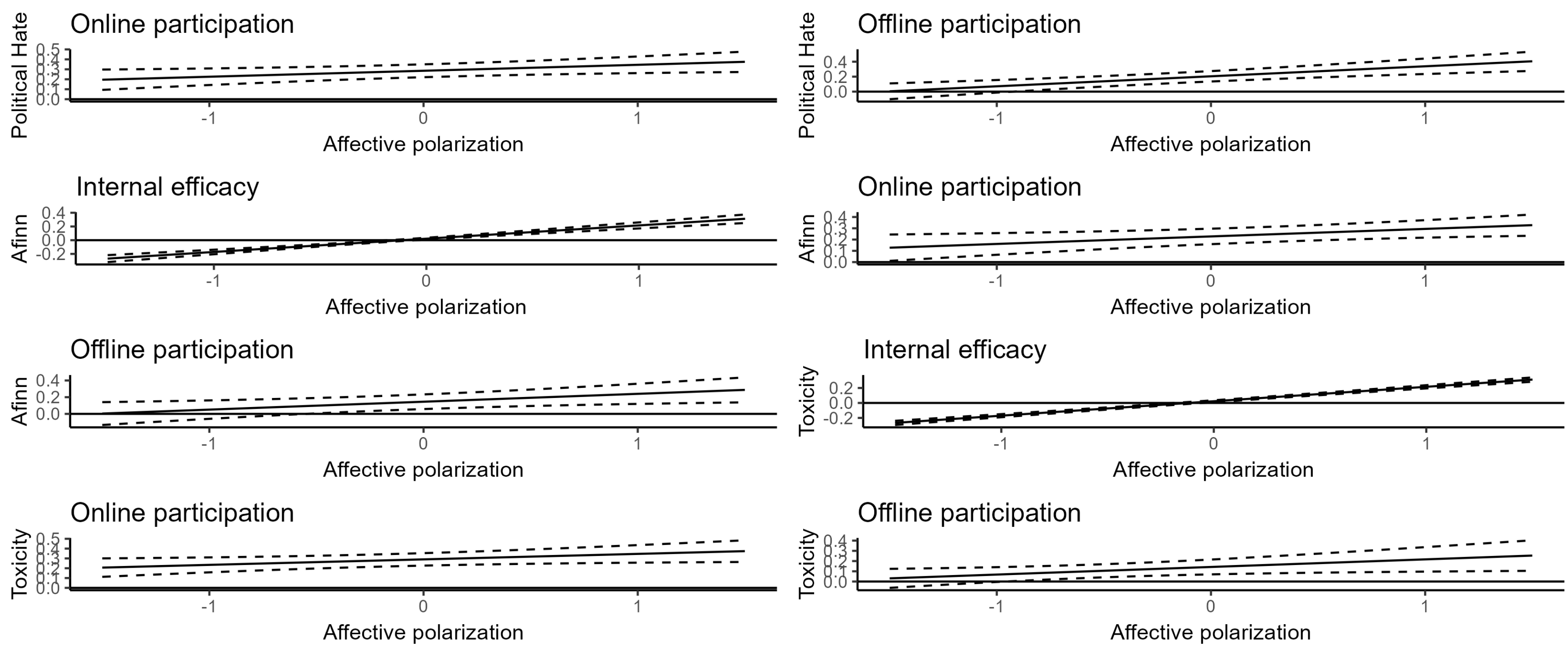

Figure 16: Interaction coefficients between indicators of political engagement, its antecedents, and leader-directed affective polarization on various measures of online hostility

Parameter	Cut point	Estimate	Standard error	N
Hate → Hate	0,0.7	0.117	0.014	2378.0
Political → Hate	0,0.7	0.035	0.011	2378.0
Hate → Political	0,0.7	0.042	0.015	2378.0
Political →Political	0,0.7	0.116	0.013	2378.0
Hate → Hate	0.01,0.69	0.117	0.014	2378.0
Political → Hate	0.01,0.69	0.035	0.011	2378.0
Hate → Political	0.01,0.69	0.042	0.015	2378.0
Political →Political	0.01,0.69	0.116	0.013	2378.0
Hate → Hate	0.02,0.68	0.117	0.014	2378.0
Political → Hate	0.02,0.68	0.036	0.011	2378.0
Hate → Political	0.02,0.68	0.042	0.015	2378.0
Political →Political	0.02,0.68	0.116	0.013	2378.0
Hate → Hate	0.03,0.67	0.115	0.014	2378.0
Political → Hate	0.03,0.67	0.037	0.011	2378.0
Hate → Political	0.03,0.67	0.04	0.014	2378.0
Political →Political	0.03,0.67	0.117	0.013	2378.0
Hate → Hate	0.04,0.66	0.116	0.014	2378.0
Political → Hate	0.04,0.66	0.039	0.012	2378.0
Hate → Political	0.04,0.66	0.037	0.014	2378.0
Political →Political	0.04,0.66	0.117	0.013	2378.0
Hate → Hate	0.05,0.65	0.114	0.014	2377.0
Political → Hate	0.05,0.65	0.042	0.012	2377.0
Hate → Political	0.05,0.65	0.036	0.014	2377.0
Political →Political	0.05,0.65	0.117	0.013	2377.0
Hate → Hate	0.06,0.64	0.115	0.014	2377.0
Political → Hate	0.06,0.64	0.043	0.012	2377.0
Hate → Political	0.06,0.64	0.035	0.013	2377.0
Political →Political	0.06,0.64	0.117	0.013	2377.0
Hate → Hate	0.07,0.63	0.11	0.014	2377.0
Political → Hate	0.07,0.63	0.048	0.013	2377.0
Hate → Political	0.07,0.63	0.033	0.013	2377.0
Political →Political	0.07,0.63	0.118	0.013	2377.0
Hate → Hate	0.08,0.62	0.116	0.014	2377.0
Political → Hate	0.08,0.62	0.055	0.013	2377.0
Hate → Political	0.08,0.62	0.034	0.012	2377.0
Political →Political	0.08,0.62	0.122	0.013	2377.0
Hate → Hate	0.09,0.61	0.116	0.014	2377.0
Political → Hate	0.09,0.61	0.054	0.014	2377.0
Hate → Political	0.09,0.61	0.033	0.012	2377.0
Political →Political	0.09,0.61	0.121	0.013	2377.0
Hate → Hate	0.1,0.6	0.117	0.014	2377.0
Political → Hate	0.1,0.6	0.056	0.014	2377.0
Hate → Political	0.1,0.6	0.034	0.011	2377.0
Political →Political	0.1,0.6	0.12	0.013	2377.0

Parameter	Cut point	Estimate	Standard error	N
Afinn → Afinn	-0.55,0.75	0.087	0.013	2792.0
Political → Afinn	-0.55,0.75	0.059	0.021	2792.0
Afinn → Political	-0.55,0.75	0.003	0.009	2792.0
Political → Political	-0.55,0.75	0.154	0.011	2792.0
Afinn → Afinn	-0.54,0.74	0.084	0.013	2789.0
Political → Afinn	-0.54,0.74	0.058	0.021	2789.0
Afinn → Political	-0.54,0.74	0.004	0.009	2789.0
Political → Political	-0.54,0.74	0.155	0.011	2789.0
Afinn → Afinn	-0.53,0.73	0.086	0.013	2786.0
Political → Afinn	-0.53,0.73	0.058	0.021	2786.0
Afinn → Political	-0.53,0.73	0.001	0.009	2786.0
Political → Political	-0.53,0.73	0.157	0.011	2786.0
Afinn → Afinn	-0.52,0.72	0.086	0.013	2785.0
Political → Afinn	-0.52,0.72	0.062	0.021	2785.0
Afinn → Political	-0.52,0.72	0.001	0.009	2785.0
Political → Political	-0.52,0.72	0.155	0.011	2785.0
Afinn → Afinn	-0.51,0.71	0.087	0.013	2783.0
Political → Afinn	-0.51,0.71	0.059	0.022	2783.0
Afinn → Political	-0.51,0.71	0.0	0.009	2783.0
Political → Political	-0.51,0.71	0.156	0.011	2783.0
Afinn → Afinn	-0.5,0.7	0.089	0.013	2783.0
Political → Afinn	-0.5,0.7	0.056	0.022	2783.0
Afinn → Political	-0.5,0.7	-0.001	0.009	2783.0
Political → Political	-0.5,0.7	0.156	0.011	2783.0
Afinn → Afinn	-0.49,0.69	0.096	0.013	2781.0
Political → Afinn	-0.49,0.69	0.053	0.022	2781.0
Afinn → Political	-0.49,0.69	0.0	0.009	2781.0
Political → Political	-0.49,0.69	0.157	0.011	2781.0
Afinn → Afinn	-0.48,0.68	0.1	0.013	2781.0
Political → Afinn	-0.48,0.68	0.06	0.022	2781.0
Afinn → Political	-0.48,0.68	0.001	0.009	2781.0
Political → Political	-0.48,0.68	0.157	0.011	2781.0
Afinn → Afinn	-0.47,0.67	0.102	0.013	2780.0
Political → Afinn	-0.47,0.67	0.06	0.022	2780.0
Afinn → Political	-0.47,0.67	0.0	0.009	2780.0
Political → Political	-0.47,0.67	0.158	0.011	2780.0
Afinn → Afinn	-0.46,0.66	0.104	0.013	2776.0
Political → Afinn	-0.46,0.66	0.074	0.022	2776.0
Afinn → Political	-0.46,0.66	0.005	0.009	2776.0
Political → Political	-0.46,0.66	0.163	0.011	2776.0
Afinn → Afinn	-0.45,0.65	0.105	0.013	2776.0
Political → Afinn	-0.45,0.65	0.075	0.023	2776.0
Afinn → Political	-0.45,0.65	0.007	0.009	2776.0
Political → Political	-0.45,0.65	0.163	0.011	2776.0

Parameter	Cut point	Estimate	Standard error	N
Toxicity → Toxicity	0,0.55	0.113	0.013	2810.0
Political →Toxicity	0,0.55	0.043	0.019	2810.0
Toxicity → Political	0,0.55	0.018	0.009	2810.0
Political → Political	0,0.55	0.135	0.011	2810.0
Toxicity → Toxicity	0,0.56	0.109	0.012	2810.0
Political →Toxicity	0,0.56	0.049	0.019	2810.0
Toxicity → Political	0,0.56	0.019	0.009	2810.0
Political → Political	0,0.56	0.136	0.011	2810.0
Toxicity → Toxicity	0,0.57	0.11	0.012	2811.0
Political →Toxicity	0,0.57	0.05	0.019	2811.0
Toxicity → Political	0,0.57	0.018	0.009	2811.0
Political → Political	0,0.57	0.136	0.011	2811.0
Toxicity → Toxicity	0,0.58	0.111	0.012	2811.0
Political →Toxicity	0,0.58	0.05	0.019	2811.0
Toxicity → Political	0,0.58	0.02	0.009	2811.0
Political → Political	0,0.58	0.136	0.011	2811.0
Toxicity → Toxicity	0,0.59	0.112	0.012	2811.0
[illegible]	[illegible]	[illegible]	[illegible]	[illegible]

3 Appendix 3: Exploratory results for network analyses

This appendix presents exploratory results for the network part of the analysis.

First, we provide descriptive summaries to accompany Figure **??**. Second, we include a descriptive network visualization for qualitative context; we do not draw inferences about centrality, influence, or echo chambers.

3.1 Measures

We use the *density*, *diameter*, and the *largest component* to measure network *cohesion*. Density refers to [25, 55] "the frequency of realized edges relative to potential edges," where edges simply refers to whether there is an "arrow" pointing to or from a person ("vertice") in our X panel. This measure is scaled to range from 0 to 1, where a higher score indicates that a person has more edges and therefore a denser network. The *diameter* of the graph measures the longest possible path between two vertices [25]; if the path is longer, more people in the network are connected. And, finally, we use the *largest component* to extract the largest number of connected X friends/followers among those low and high on political hostility: The larger this metric, the more connected the network is. We scale it to represent the *proportion* out of the total number of vertices. If social media users cluster together in echo chambers, we should observe multiple "centers of gravity" in the networks, such that those who are low on political hostility should have one or more centers of gravity and those who are high on political hostility should have one or more centers of gravity as well. To investigate this, we calculate so-called "hub scores" and "authority scores," which summarize how a given vertice is connected within the graph (we present these as descriptive, exploratory quantities and do not interpret them as measures of influence). Loosely speaking, an authority is a vertice with many arrows pointing towards it and a hub is a vertice that points to many authorities [25]. We present these graphs and analyses in the appendix.

3.2 Results

The density of the network is much higher for those who are politically hateful (6.59e−4) compared to those low on political hostility (5.59e−5). The diameter – that is, the longest path – is also higher for those high on political hostility, and the largest component among those who are high on political hostility amounts to roughly 10 % of the total number of vertices, whereas it only accounts for roughly 1 % among those low on political hostility. These indicators are descriptive; we do not draw structural conclusions from them.

Density	Largest component share of total	Diameter	Level of political hate
6.59e-04	0.09	9.0	High
8.20e-05	0.01	3.0	Low

Table 22: Measures of connectedness for networks of political hostility followers

Turning to the visualization in Figure 17, we illustrate hub and authority scores for context. The coloring of vertices (darker colors indicate higher political hostility) shows that some users with higher hostility also have higher hub/authority scores. We present the ten highest hub scores and authority scores (Tables 23 and 24). These visuals are descriptive and should not be interpreted as evidence about influence or network structure.

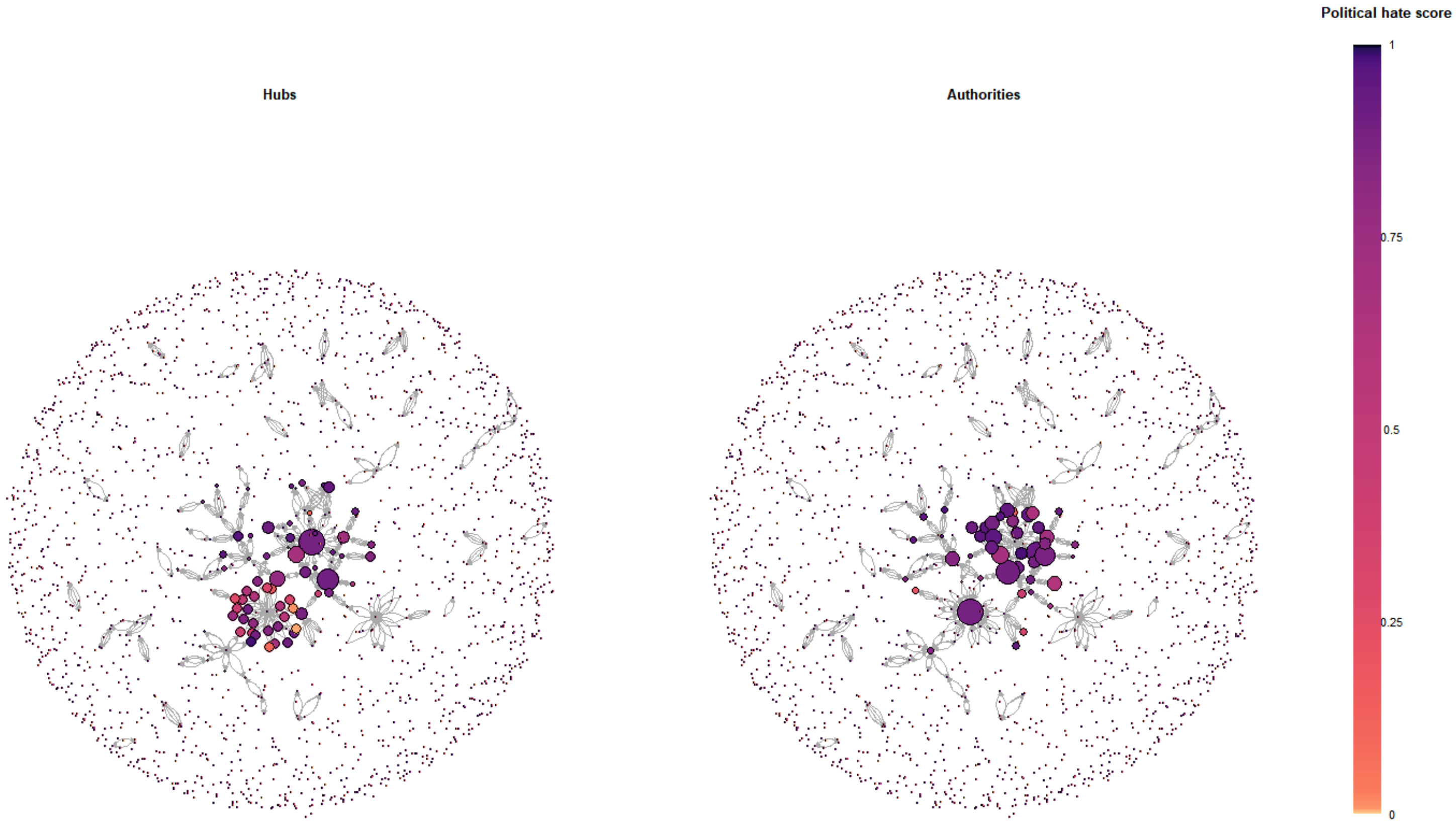

Figure 17: Network visualization of hubs and authorities in the network containing friends and followers who are also survey respondents. The color indicates the level of political hostility and the size of the nodes represent their authority/hub score. The layout is based on the Kamada-Kawai algorithm [26]. The grey color represents the connections ("edges") between the network.

Political hate score	Hub score
0.66	1.0
0.67	0.66
0.54	0.4
0.59	0.34
0.55	0.24
0.65	0.24
0.67	0.23
0.66	0.19
0.7	0.17
0.62	0.17

Table 23: Top ten hub scores

Political hate score	Authority score
0.66	1.0
0.66	0.8
0.69	0.62
0.65	0.59
0.54	0.46
0.72	0.4
0.66	0.32
0.55	0.32
0.71	0.31
0.71	0.3

Table 24: Top ten authority scores

Fig **??** provides a descriptive visualization of connections among study participants split by high and low political hostility. This figure is illustrative only; we do not make claims about central positions, echo chambers, or connectivity beyond this qualitative view.

Figure 18: **Network visualization of those with high and low levels of political hostility.** The size of vertices indicates the number of friends/followers and the color the level of political hostility of the respondent using the "magma" color palette. The layout used is a "ring" layout. The figure is descriptive and does not imply conclusions about clustering or echo chambers.

4 Appendix 4: Additional results for count models

Some relevant evidence already exists for the assertion that hostile users can be visible users. Prior work finds that hateful users may form cohesive networks and that their posts can spread widely [27, 28]. Such studies also report central positions for these users in some settings [27, 28]. Finally, [28,] find that hateful users tend to tweet more than typical users and have more friends, but fewer followers. Importantly, none of these studies investigates *political* hostility, and we do not test these network properties here; our network materials are descriptive only.

4.1 Methods and measures

The models do not take the time aspect into account but are classical count models. To investigate whether those who are more politically hostile also tweet more and have more friends/followers, we estimated, for each dependent variable, a Poisson regression, a negative binomial regression, and a zero-inflated negative binomial regression. The model with the lowest BIC was chosen [29]; see Appendix 1 Tables 11-12. All analyses were implemented in Mplus version 8. Before running the analyses we removed the five percent most extreme counts for each dependent variable separately in order to not make the analyses hinge on a few extreme observations.[1]

For the network analyses, we used *igraph* [8] to analyze and graph the results. If those who are most and least hostile tend to cluster together with their like-minded peers, we would observe relatively distinct networks (echo chambers). To explore this qualitatively, we split our X panelists into two equally sized groups based on their level of political hostility and present descriptive measures of network cohesion and a visualization. We do not report formal centrality or community metrics here.

4.2 Results

4.2.1 Counts of tweets, friends, and followers

The first set of results concerns the count models. Table 25 gives results from models that regress participants' number of followers, friends, and tweets on our measure of political hostility. These models show that political hostility is associated with both the number of followers and number of tweets and, to a lesser extent, number of friends. These analyses are exploratory and descriptive.

Model	parameters	Number of followers	Number of tweets	Number of friends
Count model	Political Hate	2.575<br>[2.147;3.003]*	3.663<br>[3.105;4.221]*	1.320<br>[0.961;1.679]*
	Intercept	4.109<br>[3.886;4.332]*	4.159<br>[3.860;4.457]*	5.458<br>[5.269;5.647]*
Inflation model	Political Hate			-11.022<br>[-14.317;-7.728]*
	Intercept			0.180<br>[-0.976;1.336]
	Number of individuals	3479	3439	1835

Table 25: Negative binomial regression models for political hate

In order to illustrate the association between political hostility and the number of tweets, followers, and friends, Figure 4 calculates predicted values when varying the level of political hostility above and below two standard deviations from the mean. These analyses are exploratory and descriptive. The association between political hostility and activity is sizeable. As the left panel of Figure 4 shows, changing from -2SD to +2SD on political hostility is associated with an increase in the number of tweets from around 150 to around 1150 – an increase of roughly seven and half times the number of tweets. The middle panel shows that higher political hostility is associated with an increase in the number of followers from around 120 to around 420; that is, roughly 3.5 times the number of followers. As the right panel shows, the association

[1] For the investigation of whether those who are politically hostile also tweet more, we only used the respondents from the first wave and only investigate the year 2019 since we were scraping these respondents continually in this period and there is thus not a problem with "exposure."

between political hostility and the number of friends is more modest, with an increase from roughly 300 to 600 – a doubling of the number of friends.

These results suggest that users who are politically hostile may have greater activity and potential reach on X/Twitter, insofar as they tweet more and are likely to reach more followers and, to a lesser extent, more friends. These analyses are exploratory and descriptive, and we do not interpret them as evidence of formal network influence or centrality.

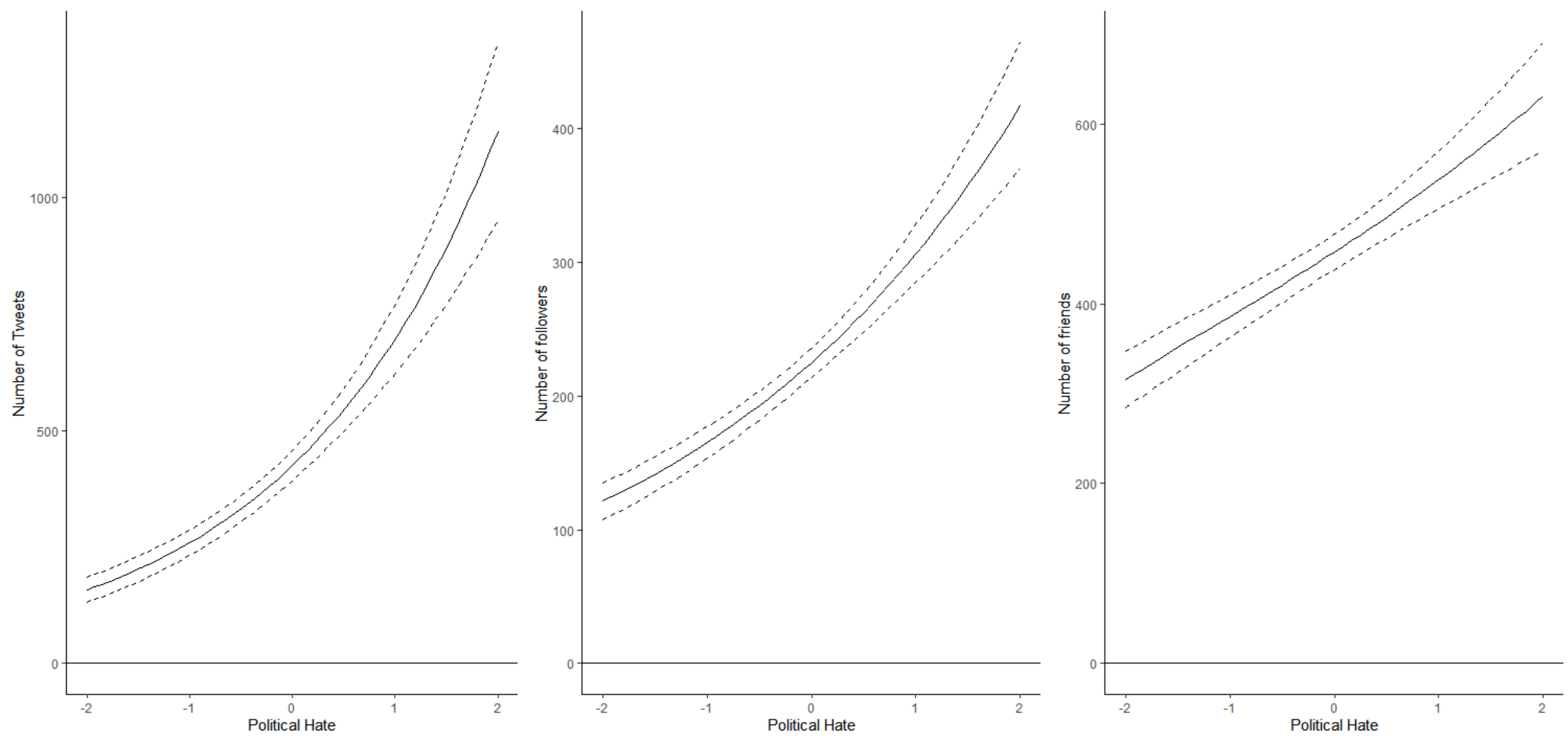

Figure 19: Predicted counts as a function of political hostility

5 Appendix 5: Missing data

Missing values are a source of potential bias in the estimation of any statistical parameter. The classical solution of simply "ignoring" this source of bias by dropping the missing values (listwise deletion) is probably the worst solution to this problem [30]. Our main goal is thus to minimize the bias introduced by missing values, while at the same time acknowledging that no perfect solutions to this problem exist. Using listwise deletion rests on the assumption of missing completely at random (MCAR), whereas newer approaches toward handling missing values rest on the assumption of missing at random (MAR), which is less restrictive. Simplifying somewhat, MCAR means that missing values are completely random, and thus e.g. not influenced by levels of education or other measured or unmeasured factors, whereas MAR rests on the assumption that missing values of a variable are not related to the values of that variable after taking into account (measured) variables that are predictors of missingness [30].

Two main techniques exist to deal with missing values: full information maximum likelihood (FIML) and multiple imputation (MI) [30], each with its own advantages and challenges. For most models we use FIML, since this is the most efficient in terms of having the lowest standard errors, but for the results in Figure **??** the number of missing values is quite large for some constructs, and we thus run into convergence problems. We therefore use MI for these analyses.

Before the final multiple imputation, we used autocorrelation plots to investigate at which point the data become independent [2]. We use the default convergence settings in Mplus for MI and created 50 datasets. Each regression is then run for each dataset and the results averaged automatically in Mplus. These 50 datasets contained all variables used across all models for Figure **??**, and we are thus using the same data across all models and only do one MI for this figure.

We use MI/FIML to deal with unit nonresponse, missing data across waves, and item-nonresponse (a person missing data within a wave for a given item).

[2]For a fuller exposition of the steps used in MI see [30, Chapter6-10]

6 Appendix 6: Ethical considerations

The studies were conducted in accordance with the Code of Conduct for Research Integrity of the primary investigator's university and associated policies of the National Committee for Health Research Ethics.

All studies were conducted online and involved voluntary and informed consent with the possibility of ending participation at any time. Potential respondents were initially selected based on their willingness to share their X id and link their X profile to their survey responses. Before survey respondents were asked to participate in the actual survey they were informed about the content of the project, who the project responsible was and how to contact them, how to withdraw their consent at a later stage, that participation was voluntary, and they were given the option to decline to participate in in the survey. Before given a chance to provide their X id, further in the survey, a detailed description of the research project was again presented to the respondents, such that they could make an informed choice about whether they wanted to participate again. Finally the respondents were informed that none of the X data would be presented in any form outside the research project that would make them personally identifiable.

Participants were paid 500 YouGov points (which roughly translates into $6) for completing the 15-minutes YouGov surveys, which is consistent with standard pay rates of the YouGov survey agency.

7 Appendix 7: Random Intercept Cross-Lagged Panel Models for Behavioral Measures of Online Engagement

The main analyses are not without limitations, as they rely on observational data and are influenced by problems of confounding and reverse causality [31]. Furthermore, the analyses rely on self-reported explanatory variables, which can be biased due to social desirability or measurement error [32].

To increase the internal validity of the conclusions, we turn to so-called Random Intercept Cross-Lagged Panel Models (RI-CLPM) [33] by using the panel structure of the dataset. The RI-CLPM allow us to investigate the reciprocal relationships between political engagement and online hostility over time more thoroughly than our previous analysis. That is, we can ask: Are within-person increases in political engagement followed by online hostility, or is online hostility followed by changes in citizens' engagement? These temporal within-person associations strengthen the descriptive evidence but do not establish causal effects.

Why use the RI-CLPM? Classical cross-lagged models have been criticized for not distinguishing clearly among variation *between* individuals and *within* individuals since the parameter estimates from such a model are a *mix* of the two [33]. The RI-CLPM, in contrast, split the variation into separate estimates for the between variance and within variance. This approach is akin to a fixed effect model – which subtracts the (within-group) mean to isolate the within-individuals variation – and is accomplished by estimating a random intercept for each construct so that the cross-lagged parameter estimates are based only on within-individual variation. The result is a model that can investigate reciprocal relationships, which is not possible in a fixed effect model, as well as hold (time-invariant and possibly unobserved) dispositional traits constant. Time-varying confounding cannot be ruled out [34].

Furthermore, to increase the measurement validity of the conclusions, we turn towards behavioral measures of political engagement rather than merely analyzing survey based measures. Making fuller use of the available data, we are able to repeat these analyses using two *behavioral* measures of online political engagement. First, we exploit the fact that the measure of "political hostility" can be split into two [35]: A word vector of words associated with "political" and a vector of words associated with "hate." We use these two vectors to examine whether people who often tweet about politics – i.e., those who are politically engaged – are also more likely to disseminate hateful content. Again we also use the measures of toxicity and Afinn scores as a replacement for the hate vector to gauge the robustness of the findings.

7.1 Methods and measures

The previous analyses have used the survey waves as the unit of analysis. Here, we are not limited to that since we have daily data on hundreds of X users. To make full use of this, we therefore organize the dataset in terms of months to ensure reliable measures of "political" and "hate" that are not overly influenced by any single tweet but are still highly fine-grained.

The RI-CLPM are estimated in Mplus version 8. Since the RI-CLPM rest on the assumption of normally distributed variables [36], and in order to reduce the influence of outliers, we remove the most extreme observations (see Figures 10-13 in Appendix 1).[3] In the current set-up the model also assumes no measurement error but since the model at each time point relies on a month worth of data this increases the reliability of the measures. The model includes measures of both stability and change. Positive stability scores indicate that those who were (hateful or engaged) at $time_1$ were also likely to be more hateful or engaged relative to their own expected mean at $time_{+1}$. Conversely the reciprocal coefficients, i.e. the cross-lagged paths from online hostility to political engagement (online hostility $\rightarrow$ political) or from engagement to online hostility (political $\rightarrow$ online hostility) indicate that a (positive) deviation from an individual's expected mean in e.g. political engagement or online hostility is associated with a (positive) deviation in the expected mean of political engagement or online hostility at the next time point.

The measures of toxicity scores and Afinn scores are based on data from [35] and thus based on the time period from March 2019 until March 2020. Although we have full data for both our hostility and political measures, we only focus on the period March 2020 until January 2021 since this is the period during which we collected measures of affective polarization. The scores thus represent averages for each month in this period. Since we are also interested in investigating whether these behavioral measures of political

[3] We investigate the robustness of these choices (see Figures 7-9) in Appendix 1

engagement are moderated by subjective feelings of discontent, we include the average score of affective polarization in a second set of analyses to investigate whether the relationships differ by leader-directed affective polarization, as above. We follow [37,] in using a multiple group approach towards investigating moderation hypotheses for the RI-CLPM. Put simply, we create a RI-CLPM separately for those high and low on affective polarization to investigate whether the reciprocal relationships between engagement and hostility change as a function of affective polarization.

7.2 Results

Figure 20 illustrates the reciprocal relationships between our three measures of online hostility (hate, Afinn scores, and toxicity scores) and our measure of online engagement, i.e. our political vector. We first notice that there is a fair amount of individual-level stability over time, indicated by the positive and significant stability estimates (political → political and online hostility → online hostility). This means that X users who have been hostile or politically engaged at a previous point in time tend to remain hostile or politically engaged at later time points. More importantly, we find that within-person increases in political engagement are followed by higher levels of all three measures of online hostility. An increase in political engagement from zero to one, for example, is associated with an increase of 0.088 (95 % CI [0.062;0.113]) in hate on a zero to one scale. This finding is consistent with the claim that higher political engagement is associated with higher hostility, though it does not by itself establish a causal effect.

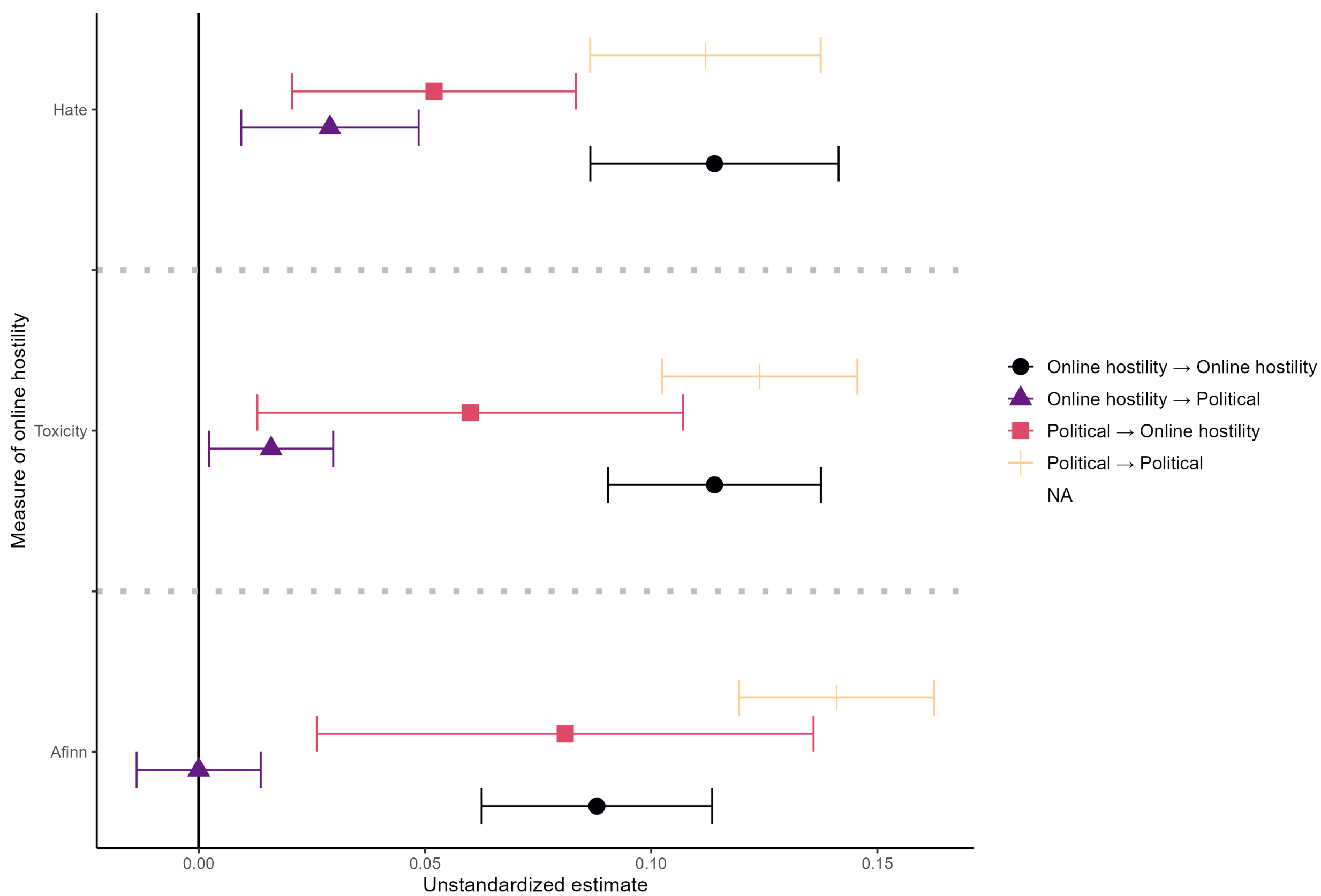

Figure 20: RI-CLPM for online hostility. Full results can be found in Table 20

However, we also find that online hostility, measured using the "hate" word vector and toxicity scores, is followed by higher political engagement (although hostility measured by Afinn scores is not). Still, the estimated associations are generally smaller compared to the reverse association. This finding is perhaps not surprising. The findings above indicated that the association between political engagement and political

hostility differs by levels of leader-directed affective polarization. We would thus expect the association of online hostility with subsequent political engagement to be most apparent for those who are most affectively polarized. This is also generally consistent with the data, as shown in Figures 14-15 in the Appendix. The association of online hostility (measured using toxicity scores and hate) with political engagement is only significant for those high on affective polarization but not for those low on affective polarization.[4]

[4]The effects are illustrated for the average effect using cut points in the range [0,5;0.65] where affective polarization is measured on a zero to one scale, since we have no a priori guess for which cutpoint to choose.

8 Appendix 8: Additional correlations for Denmark

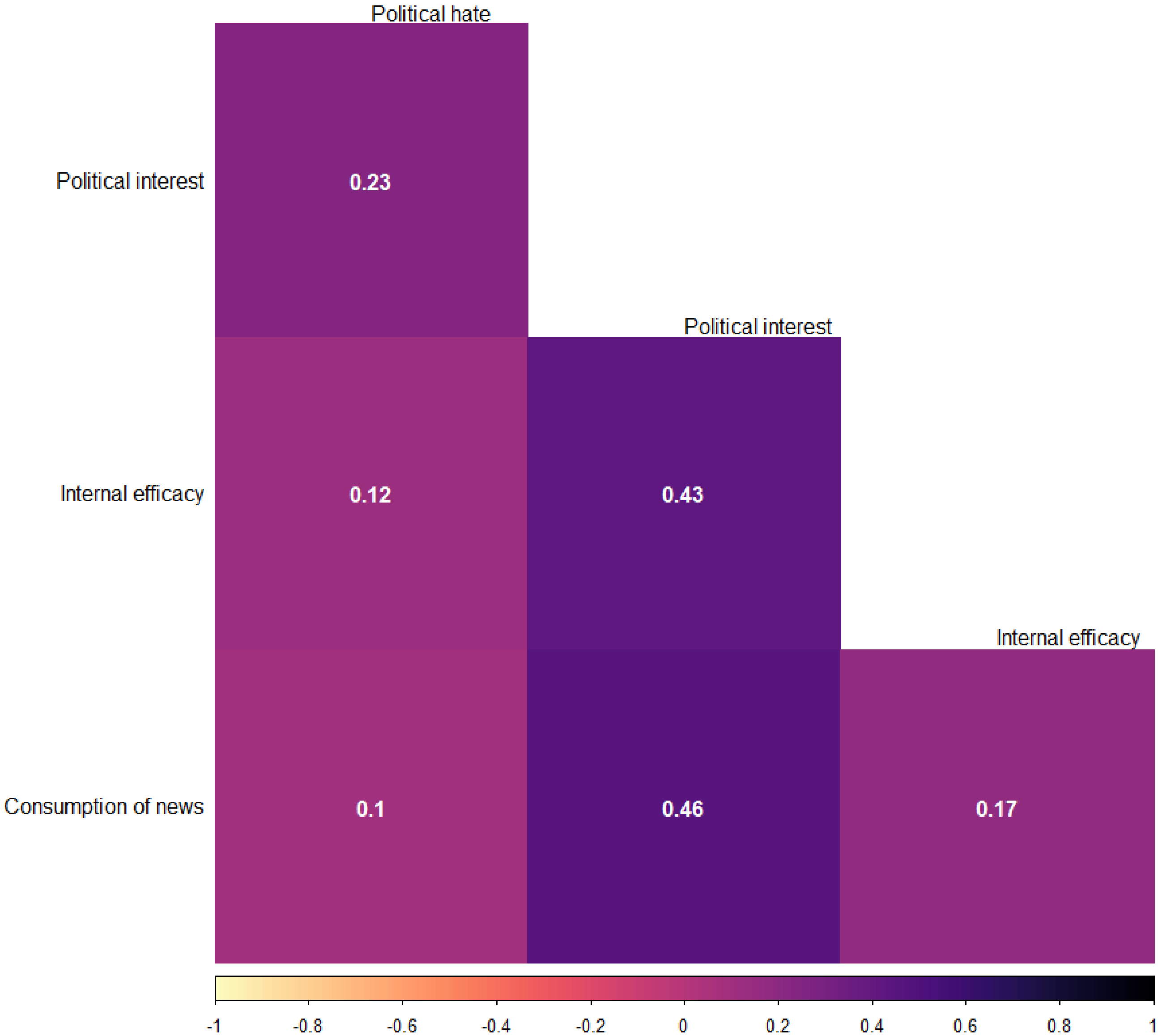

Table 26: Correlations between measures of online hostility and self-reported measures in survey in Denmark

All correlations are significant at a .05 level. The measures used in this analyses are based on standardized measures of these constructs in a Danish context. *Political interest* is measured asking the question "How interested are you in politics", with these answer categories: Very interested, fairly interested, almost not interested, not at all interested, and don't know, using the categories from the Danish Election Studies. The question on *consumption of news* is based on the following question: "How long time do you approximately spend on looking at, reading or listening to political news and current issues on a typical day. I.e. both in news papers, radio, tv and on the internet. Please answer in hours and minutes." The measure of *internal efficacy* is based on two questions. The first question asks: "How much do you think the political system in Denmark provides people like you the opportunity to speak up in terms of what the government does." The second asks: "How much do you think the political system in Denmark makes it possible for people like you to exert an influence on politics?" Both questions use these answer categories: Very much, much, somewhat, very little, not at all.

The data comprises responses from surveys conducted between September 27, 2021, and March 23, 2022. Participants were reached through their digital mailboxes, where they received an invitation titled "Help

research understand hate on social media". This invitation detailed the study's aim to explore the origins and impacts of online hate, and to formulate strategies to mitigate its effects.

The survey targeted a cross-section of the adult Danish population, drawn from a group of 300,000 individuals as identified by Statistics Denmark. From this group, 16,026 were unable to receive electronic mail in their digital mailbox, reducing the effective sample size to 283,974.

In total, 24,996 individuals responded to the survey, equating to a response rate of about 9 percent (24,996/283,974). The relatively low rate of response may be attributed to the fact that the survey invitations were only distributed via digital mailboxes without subsequent reminders, coupled with a potential general fatigue from survey participation due to the coronavirus pandemic

We utilized social security numbers to access public records from various state registries, details of which can be found in https://osf.io/kz7p3/

In order to link the 300,000 individuals to X accounts, we narrowed our dataset to those with a unique combination of first, middle, and last names. We then employed these names to query the X API. When a single X profile correspondingly matched a name, we considered it a successful match. This process resulted in identifying 24,356 distinct X users, of whom 4,931 had posted at least once. Our dataset comprises 3.4 million tweets. To assess levels of hostility, we applied the super-unsupervised approach also used in [35]. Further validation of this matching method can be found in [38].

References

[1] Nie NH, Junn J, Stehlik-Barry K. Education and democratic citizenship in America. Chicago: The University of Chicago Press; 1996.

[2] Mondak JJ. Personality and the foundations of political behavior. Cambridge University Press; 2010.

[3] Verba S, Schlozman KL, Brady HE. Voice and equality: Civic voluntarism in American politics. Harvard University Press; 1995.

[4] Muthén L, Muthén B. Mplus User's Guide. Eighth Edition.; 2017.

[5] R Core Team. R: A Language and Environment for Statistical Computing. Vienna, Austria; 2020.

[6] Hallquist MN, Wiley JF. MplusAutomation: An R Package for Facilitating Large-Scale Latent Variable Analyses in Mplus. Structural Equation Modeling. 2018:1-18.

[7] Wickham H. ggplot2: Elegant Graphics for Data Analysis. Springer-Verlag New York; 2016.

[8] Csardi G, Nepusz T. The igraph software package for complex network research. InterJournal. 2006;Complex Systems:1695. Available from: `https://igraph.org`.

[9] Niemi RG, Craig SC, Mattei F. Measuring internal political efficacy in the 1988 National Election Study. The American Political Science Review. 1991:1407-13.

[10] Denissen JJ, Penke L. Motivational individual reaction norms underlying the Five-Factor model of personality: First steps towards a theory-based conceptual framework. Journal of research in personality. 2008;42(5):1285-302.

[11] DeYoung CG. Intelligence and personality. 2020.

[12] John OP, Naumann LP, Soto CJ. Paradigm shift to the integrative Big Five trait taxonomy: History, measurement, and conceptual issues. 2008.

[13] Krueger RF, Johnson W. Behavioral genetics and personality: A new look at the integration of nature and nurture. 2008.

[14] Watson D, Clark LA. On traits and temperament: General and specific factors of emotional experience and their relation to the five-factor model. Journal of personality. 1992;60(2):441-76.

[15] DeYoung CG. Toward a theory of the Big Five. Psychological Inquiry. 2010;21(1):26-33.

[16] Marcus GE, Theiss-Morse E, Sullivan JL, Wood SL. With malice toward some: How people make civil liberties judgments. Cambridge University Press; 1995.

[17] DeYoung CG. Mapping personality traits onto brain systems: BIS, BAS, FFFS and beyond. 2010.

[18] Druckman JN, Levendusky MS. What Do We Measure When We Measure Affective Polarization? Public Opinion Quarterly. 2019;83(1):114-22.

[19] Iyengar S, Lelkes Y, Levendusky M, Malhotra N, Westwood SJ. The Origins and Consequences of Affective Polarization in the United States. Annual Review of Political Science. 2019 May;22(1):129–146.

[20] Dekker H, Meijerink F. Political cynicism: Conceptualization, operationalization, and explanation. PCS–Politics, Culture and Socialization. 2013;3(1+ 2):9-10.

[21] Asparouhov T, Muthén B. Exploratory structural equation modeling. Structural equation modeling: a multidisciplinary journal. 2009;16(3):397-438.

[22] Hu Lt, Bentler PM. Cutoff criteria for fit indexes in covariance structure analysis: Conventional criteria versus new alternatives. Structural equation modeling: a multidisciplinary journal. 1999;6(1):1-55.

[23] Byrne BM. Structural equation modeling with EQS and EQS/Windows: Basic concepts, applications, and programming. Sage; 1994.

[24] Schumacker RE, Lomax RG. A beginner's guide to structural equation modeling. psychology press; 2004.

[25] Kolaczyk ED, Csárdi G. Statistical analysis of network data with R. vol. 65. Springer; 2014.

[26] Kamada T, Kawai S, et al. An algorithm for drawing general undirected graphs. Information processing letters. 1989;31(1):7-15.

[27] Mathew B, Dutt R, Goyal P, Mukherjee A. Spread of hate speech in online social media. In: Proceedings of the 10th ACM conference on web science; 2019. p. 173-82.

[28] Ribeiro MH, Calais PH, Santos YA, Almeida VA, Meira Jr W. " Like Sheep Among Wolves": Characterizing Hateful Users on Twitter. arXiv preprint arXiv:180100317. 2017.

[29] Muthén BO, Muthén LK, Asparouhov T. Regression and mediation analysis using Mplus. Muthén & Muthén Los Angeles, CA; 2017.

[30] Enders CK. Applied missing data analysis. Guilford press; 2010.

[31] Angrist JD, Pischke JS. Mostly harmless econometrics: An empiricist's companion. Princeton university press; 2008.

[32] Holbrook AL, Krosnick JA. Social desirability bias in voter turnout reports: Tests using the item count technique. Public Opinion Quarterly. 2010;74(1):37-67.

[33] Hamaker EL, Kuiper RM, Grasman RP. A critique of the cross-lagged panel model. Psychological methods. 2015;20(1):102.

[34] Imai K, Kim IS. When should we use unit fixed effects regression models for causal inference with longitudinal data? American Journal of Political Science. 2019;63(2):467-90.

[35] Rasmussen SHR, Bor A, Osmundsen M, Petersen MB. 'Super-unsupervised'classification for labelling text: online political hostility as an illustration. British Journal of Political Science. 2024;54(1):179-200.

[36] Usami S, Murayama K, Hamaker EL. A unified framework of longitudinal models to examine reciprocal relations. Psychological methods. 2019;24(5):637.

[37] Mulder JD, Hamaker EL. Three extensions of the random intercept cross-lagged panel model. Structural Equation Modeling: A Multidisciplinary Journal. 2021:1-11.

[38] Hebbelstrup Rye Rasmussen S, Bor A, Petersen MB. The offline roots of online hostility: Adult and childhood administrative records correlate with individual-level hostility on Twitter. Proceedings of the National Academy of Sciences. 2024;121(44):e2412277121.
