# Supplementary material for: Political engagement and online political hostility among American X/Twitter users
Source: PLoS One. 2026 Nov 11;21(11):e0360061. doi: 10.1371/journal.pone.0360061 (PMC13754149; doi:10.1371/journal.pone.0360061)
Supplement: Supplementary file 3 [file pone.0360061.s003.docx]

# Response to the Academic Editor

**Comment:** The revisions addressed most previous concerns, but additional weaknesses remained in the theoretical background and some causal wording still needed to be toned down.

**Response:** We thank the Academic Editor for this assessment and for the opportunity to revise the manuscript. We have made a set of targeted revisions to sharpen the theoretical framing and further reduce causal language throughout the manuscript. Specifically, we now distinguish between distal antecedents of political engagement, such as education and internal efficacy; motivational and cognitive indicators of engagement, such as political interest and political knowledge; and participatory engagement, such as online and offline participation. We also added a new section on empirical expectations, which states the expectations in explicitly associational terms. In addition, we conducted a manuscript-wide audit of causal language. The title, abstract, introduction, analytical approach, results, RI-CLPM section, discussion, and appendix now consistently describe the findings as observational associations. The RI-CLPM analyses are presented as temporal within-person evidence that reduces concerns about stable time-invariant confounding but does not establish causal effects. We also expanded the discussion of alternative causal models, especially with respect to political sophistication, partisan involvement, and leader-directed affective polarization.

# Journal requirements and additional submission items

**Comment:** Please review the reference list for completeness and correctness, including any retracted articles, and submit the revised manuscript, tracked-changes manuscript, and response letter.

**Response:** We have reviewed the reference list for completeness and correctness and updated the references where necessary to reflect the revised theoretical discussion and measurement discussion. We have not knowingly cited any retracted articles. The revised submission includes a clean manuscript, a marked-up manuscript with tracked changes, and this point-by-point response letter. The Data Availability statement has also been updated to provide the OSF repository for the anonymized minimal dataset and analysis code. Full post-level X/Twitter data remain restricted because of re-identification risks and X Terms of Service restrictions, as described in the manuscript. No changes were made to the financial disclosure statement.

**Comment:** If applicable, deposit laboratory protocols in protocols.io and encourage coauthors to verify ORCID iDs.

**Response:** This study does not involve laboratory protocols, so protocols.io deposition is not applicable. We also appreciate the reminder regarding ORCID verification and will ensure that coauthors verify their ORCID iDs in the submission system where applicable.

# Response to Reviewer #2

**Comment:** All previous comments have been addressed. The manuscript is substantially improved. The remaining reservation concerns the construct validity of the hostility measure: the measure may capture proximity to political or hate-related language rather than hostility as such.

**Response:** We thank Reviewer #2 for the generous and constructive assessment of the revised manuscript. We are pleased that the reviewer views the manuscript as substantially improved and that the previous revisions addressed the main concerns. We also appreciate the remaining reservation regarding construct validity. In response, we added explicit language acknowledging that no automated text-based measure can perfectly separate hostility from proximity to political conflict language. The revised manuscript now states that the SU measure may partly capture proximity to politically salient conflict language or hate-related political vocabulary rather than hostility alone. At the same time, we retain the measure as our primary operationalization because it is supported by several validation exercises and because the main results are assessed against additional measures of negativity, toxicity, and supervised classification. We have also preserved the cautious framing of the network-related analyses: these analyses remain exploratory and descriptive rather than claims about formal network influence or centrality.

# Response to Reviewer #4

We thank Reviewer #4 for the careful and constructive review. We especially appreciate the reviewer’s recognition of the originality and importance of the topic, as well as the concerns about theorization, causal interpretation, and measurement. We agree that the previous version did not sufficiently distinguish among different types of predictors under the broad language of “political resources” and “political engagement.” In response, we have substantially revised the theoretical framing, added clearer empirical expectations, separated distal antecedents from proximate engagement indicators, and made the moderation logic more explicit. We have also further toned down causal language and expanded the discussion of alternative causal models.

## 1. Theoretical framing of political engagement and its antecedents

**Comment:** The theorization was muddy. The manuscript treated education, internal efficacy, interest, knowledge, and participation as a grab bag of “political resources.” It was unclear why internal efficacy or participation should cause hostility, and participation and hostility may both be caused by political sophistication.

**Response:** We agree that the previous version treated conceptually distinct variables too similarly. We have revised the theoretical section to distinguish among three categories. Education and internal efficacy are now described as distal antecedents that may equip citizens with skills, confidence, and perceived competence in politics. Political interest and political knowledge are described as motivational and cognitive indicators of engagement. Online and offline participation are described as participatory engagement, that is, behavioral expressions of engagement. We also clarified that we do not claim that internal efficacy or participation necessarily has a direct causal effect on hostility. Rather, our claim is that politically engaged citizens, and citizens with antecedents that facilitate engagement, may be more likely to enter political conflict on X/Twitter, including antagonistic exchanges. The revised manuscript explicitly discusses the alternative possibility that political sophistication or partisan involvement is a common cause of both participation and hostility. We therefore formulate the expectations in associational terms and avoid treating all indicators as a single undifferentiated resource construct. The supplementary factor analysis is also used to support the decision to treat the indicators separately rather than as one scale.

## 2. Logic and causal status of affective-polarization moderation

**Comment:** The moderation analysis using affective polarization was theoretically unclear and not causally identified. Alternative causal models needed to be discussed.

**Response:** We agree. We added a dedicated theoretical subsection, “Affective polarization as a conditional context.” In the revised manuscript, the logic is that engagement provides attention, motivation, skills, and opportunities to participate in political discussion, while affective polarization may shape whether such participation is expressed in antagonistic terms toward political opponents. We also now explicitly discuss alternative causal models. Affective polarization may increase political interest or participation; political sophistication may increase both affective polarization and hostility; or hostile online interactions may increase affective polarization over time. Because the moderation analyses are observational, we now interpret them as descriptive evidence about conditional associations rather than as causally identified moderation effects.

## 3. Strength of claims about affective polarization

**Comment:** The claims about affective polarization were too strong because only some interaction terms were statistically significant.

**Response:** We agree and have revised the wording throughout the manuscript. The abstract, results, and discussion now state that the moderation evidence is partial and uneven across indicators. The revised results section emphasizes that the conditional pattern is clearest for online and offline participation, while the evidence for more distal antecedents such as education is weaker or inconsistent. We also added multiplicity-adjusted results using Benjamini-Hochberg false-discovery-rate corrections and Bonferroni-adjusted confidence intervals. These analyses reinforce the cautious interpretation: the moderation results are most consistent for participatory engagement and should be treated as exploratory for the remaining indicators. We no longer describe the affective-polarization expectations as uniformly confirmed.

## 4. Measurement of affective polarization

**Comment:** The affective-polarization measure uses party leaders rather than parties or party supporters. This choice needed more discussion and, ideally, robustness checks using a richer measure.

**Response:** We agree that the measure required more precise description and stronger discussion. We now refer to the measure as leader-directed affective polarization. The revised measures section explains that the survey included feeling thermometer ratings for three Republican politicians and three Democratic politicians, which were combined with party identification to distinguish in-party and out-party leaders. The survey did not include parallel thermometer ratings of ordinary party supporters. We therefore could not construct the party-supporter measure suggested by the reviewer. To address this limitation, we added a discussion stating that leader-directed affective polarization may differ from party-directed or supporter-directed affective polarization, and we interpret the moderation analyses accordingly.

## 5. Big Five controls and Dark Triad accounts

**Comment:** The Big Five controls were insufficiently motivated, especially because the literature review discussed Dark Triad traits. It was unclear why Big Five traits were included rather than Dark Triad measures.

**Response:** We have revised this discussion substantially. The Big Five variables are now presented as broad control variables for stable individual differences in motivational, emotional, and cognitive dispositions, not as a direct test of Dark Triad, sadism, or antagonistic-trait accounts. We now explicitly state that the survey did not include Dark Triad or sadism measures and that the analyses therefore cannot directly adjudicate those accounts. We have also reduced the interpretive emphasis on individual Big Five coefficients in the main text and added a limitation noting that future research should examine how political engagement interacts with more specific antagonistic personality constructs.

## 6. Resource-deficit framing

**Comment:** The manuscript awkwardly framed the study against a “resource deficit” approach that lumped together personality explanations and economic marginalization. These should be distinct.

**Response:** We agree and revised the introduction and related-work section accordingly. The revised manuscript now separates socioeconomic and grievance-based explanations from personality and emotion-regulation explanations. We no longer collapse these literatures into a single resource-deficit account. Instead, the manuscript positions our contribution as complementary: online political hostility may be associated not only with deprivation, marginalization, or personality-based explanations, but also with political engagement and its antecedents.

## 7. Generalizability beyond X/Twitter

**Comment:** How generalizable is hostility on Twitter/X to other social media applications? X tends to be more overtly political than other platforms.

**Response:** We agree that the platform context matters. We revised the title, abstract, introduction, results, and discussion to describe the study as an analysis of American X/Twitter users rather than social media users in general. The revised limitations section explicitly states that X/Twitter is a public, text-based, and highly political platform, and that the associations observed here may not generalize directly to platforms with different affordances, audiences, moderation systems, or interaction norms. We now treat cross-platform generalizability as an open empirical question and call for future research on other platforms.

## 8. Multiple comparisons

**Comment:** There are many tests of effectively the same hypothesis. Confidence intervals should be adjusted for multiple comparisons.

**Response:** We agree. We added multiplicity-adjusted results in the S1 Appendix. In the analytical approach section, we now define four families of tests: primary association models, robustness outcome models, affective-polarization interaction models, and exploratory activity/reach models. Within each family we apply Benjamini-Hochberg false-discovery-rate corrections and also report Bonferroni-adjusted confidence intervals for a more conservative reading. The main figures retain conventional 95% confidence intervals for readability, while the appendix reports the adjusted results. These adjustments do not alter the central conclusion that political engagement and its antecedents are associated with online political hostility, but they reinforce the need for caution in interpreting the moderation analyses.

## 9. Clearly delineated hypotheses or expectations

**Comment:** The manuscript would benefit from clearly delineated hypotheses to aid readability.

**Response:** We agree. We added a new section on empirical expectations that states the main expectations in noncausal terms. The expectations now distinguish distal antecedents of engagement, motivational and cognitive indicators of engagement, participatory engagement, and the conditional role of leader-directed affective polarization. We also added an exploratory expectation for the activity and potential-reach analyses and updated the results headings so that the empirical sections correspond more directly to the expectations.

# Additional harmonization of manuscript and appendix

In addition to the specific changes above, we harmonized the terminology used in the main manuscript and S1 Appendix. We replaced broad references to “political resources” where that wording could obscure the distinction between distal antecedents, motivational/cognitive engagement, and participatory engagement. We revised causal or causal-sounding language in the RI-CLPM discussion, and we reframed the network/activity analyses as exploratory analyses of activity and potential reach rather than as evidence of formal influence or centrality. These changes were made to ensure that the revised manuscript consistently reflects the observational and descriptive status of the analyses.
