# Supplementary material for: Political engagement and online political hostility among American X/Twitter users
Source: PLoS One. 2026 Nov 11;21(11):e0360061. doi: 10.1371/journal.pone.0360061 (PMC13754149; doi:10.1371/journal.pone.0360061)
Supplement: Supplementary file 2 [file pone.0360061.s002.docx]

# **Response to Editor and Reviewers**

Manuscript ID: **PONE-D-25-18048**

Title: **“Political Engagement and Online Political Hostility: …”**

## **1. Cover Letter to the Academic Editor**

**Comment (Editor):** Manuscript has merit but needs (a) data & code available for review, (b) clearer ethics/IRB exemption, (c) stronger validation of hostility measure and network analysis, and (d) toned-down causal language.

**Response:**

We thank the Academic Editor for the constructive decision. In the revision we have:

1. Prepared an anonymized repository with the minimal dataset and analysis code for reviewer inspection. The future link is <https://osf.io/82guk/overview> and the view-only access can be accessed at <https://osf.io/82guk/overview?view_only=68d5ef05464b4ef7ba17b72f968d5879>
2. Provided a formal statement from the IRB institution at Aarhus University confirming that the study was not required to undergo full ethical review and, in this case, could not as the IRB institution was only formed after the data collection was initiated. We have expanded the ethics statement accordingly.
3. Reworked the measurement section so that the embedding-based hostility measure is described and validated in the main text, and we added standard metrics (precision, recall, F1, confusion matrix) and comparisons to Perspective API.
4. Narrowed the manuscript scope to focus on the core contribution—the relationship between political engagement and online hostility. Network analyses have been moved to S1 Appendix as exploratory supplementary material with appropriate methodological details and interpretive caveats.
5. Toned down causal language throughout and framed findings as associations.

We address all journal requirements and reviewer comments below.

## **2. Journal / Administrative Requirements**

### **2.1 PLOS ONE style requirements**

**Comment:** Reformat to PLOS ONE style/templates.

**Response:** The manuscript has been reformatted according to PLOS ONE guidelines (section order, figure captions, file naming).

**Manuscript changes:** Updated template and sectioning.

### **2.2 Ethical approval / exemption**

**Comment:** GDPR ≠ ethics. Provide explanation and formal waiver.

**Response:** Our data were collected through a U.S. YouGov panel in which participants gave informed digital consent and could voluntarily provide their Twitter handle for linkage to publicly available tweets. Under Danish law and Aarhus University’s procedures, this type of secondary analysis of voluntarily provided survey data linked to public social media data do not require full IRB/REC review, provided that data were processed under the university’s GDPR framework. We have now obtained a formal statement from Aarhus University’s IRB institution confirming that no further approval was required. In this particular case, approval was furthermore not possible to obtain as the institution was only formed after the data collection was initiated.

**Manuscript changes:** Ethics section expanded to explain (1) source of participants, (2) nature of consent, (3) use of public tweets, (4) institutional waiver.

### **2.3 Compliance with platform terms**

**Comment:** Add statement on terms/conditions.

**Response:** We added that Twitter (now X) data were collected only from public accounts and in accordance with the platform’s terms of service at the time of collection.

**Manuscript changes:** Methods → Data.

### **2.4 Participant consent details**

**Comment:** Specify informed/written/verbal; minors; waivers.

**Response:** We now specify that panel participants gave informed digital/written consent through the panel provider; only adults were included; no minors, no waivers.

**Manuscript changes:** Ethics paragraph in Methods.

### **2.5 Data sharing / minimal dataset**

**Comment:** Make data available now or explain restrictions and provide contact.

**Response:** We have uploaded an anonymized minimal dataset (user-wave hostility scores, main covariates) and the analysis code to an anonymized repository. Full tweet-level data cannot be made publicly available because of potential re-identification; access requests can be directed to [michael@ps.au.dk](mailto:michael@ps.au.dk).

**Manuscript changes:** Data Availability statement updated with repository + contact.

### **2.6 Missing repository / DOI**

**Response:** We added the repository name/DOI in the Data Availability statement.

### **2.7 Funding mismatch**

**Response:** We reconciled the ‘Funding Information’ and ‘Financial Disclosure’ sections so they list the same grant(s).

**Manuscript changes:** Funding section updated.

### **2.8 PLOS LaTeX template**

**Response:** Manuscript converted to the PLOS LaTeX template / reformatted accordingly.

### **2.9 Captions for Supporting Information**

**Response:** Captions for S1–Sx files added at the end of the manuscript and in-text citations updated.

### **2.10 Suggested citations**

**Response:** The reviewers did not request that we cite specific previously published works. Reviewer 1 noted a lack of references defining network concepts; however, as we have moved the exploratory network analysis to the Supporting Information (S1 Appendix) to better focus the manuscript, we did not add these definitions to the main text. Regarding Reviewer 3's comment on the limitations of sentiment dictionaries, we have updated the text to explicitly acknowledge these limitations, but no specific papers were requested for citation.

## **3. Reviewer #1**

### **1. Conceptual vagueness (“precursors”; switching terms)**

**Comment:** Terms “precursors,” “political hate,” “political hostility,” and “toxicity” are used interchangeably.

**Response:** We now define at the start of the theory section that our target construct is **online political hostility** (politically grounded degrading/intimidating content). “Political hate” is now used only as the name of the embedding vector in the SU method; “toxicity” is used only for the Perspective API robustness. We also replaced “precursors” with “antecedents/predictors.”

**Manuscript changes:** Intro + theory + measures clarified.

### **2. Justification of embedding measure**

**Comment:** No theoretical/empirical justification for "political" + "hate."

**Response:** We expanded the measurement section to explain the logic of training embeddings on the panelists' own tweets, constructing a composite vector that captures co-occurrence of political and hostile language, and then scoring tweets by cosine similarity. We now report extensive validation results in the main text, including nearest-neighbor analyses showing that the combined vector is surrounded by clearly hostile and political terms, and correlations with expert labels, Perspective API, and a supervised classifier.

**Manuscript changes:** Methods → Measures (now in main text) + short validation table.

### **3. SU method unclear**

**Comment:** How is the “overall similarity” obtained?

**Response:** We now describe the four steps (train → construct vector → compute cosine similarity per tweet → average to user-wave level) and specify that we used **averaging**.

**Manuscript changes:** Methods → Measures.

### **4. Disorganized methods**

**Comment:** Methods contain unnecessary technicalities; important parts in appendices.

**Response:** We reorganized to a standard structure (Introduction, Related Work, Data, Measures, Methods, Results, Discussion) and moved software details to the supplement; key model info is now in the main text.

**Manuscript changes:** Section order + pruning.

### **5. Weak network analysis**

**Comment:** "Centrality," "echo chamber," "influence" used without metrics; Figure 3 not informative.

**Response:** We appreciate this concern and have reconsidered the scope of our manuscript. Upon reflection, we decided to narrow our focus to the main contribution—the relationship between political engagement and online hostility—and moved the network analyses to S1 Appendix as exploratory supplementary material. In the main text, we now briefly note that exploratory network-level analyses are available in the appendix, with appropriate caveats about their limitations (including that the network is restricted to study participants). We specify the network construction details, report descriptive statistics on tweeting frequency and followers/friends, and tone down claims substantially. This approach allows us to maintain focus on our core theoretical and empirical contribution while making the network material available for interested readers without overstating its robustness.

**Manuscript changes:** Network section moved to S1 Appendix; brief mention with caveats added to main text Discussion.

### **6. Language / style / US-only data**

**Response:** We edited for tone and fixed the specific typos the reviewer noted. We added one paragraph in Data explaining that we used a U.S. panel because the theoretical mechanism is most visible in a highly polarized environment.

**Manuscript changes:** Intro, Data.

## **4. Reviewer #2**

### **1. Measurement validity (main concern)**

**Comment:** The embedding approach may capture "talking about hate + politics" rather than hostile properties; 74% accuracy is not informative.

**Response:**

We agree that an embedding approach could in principle pick up meta-talk about ‘hate’. This is why, we in the BJPS paper on the super-unsupervised method, inspected the nearest neighbours of the ‘political’, ‘hate’, and combined ‘political hate’ vectors. In our data, the neighbours are terms such as ‘hateful’, ‘racist’, ‘intolerance’, ‘supremacist’, and ‘partisan’, that is, terms that are both political and clearly hostile, indicating that the model is capturing hostility-in-use rather than mere mentions of the word ‘hate’. We now report this inspection in the main text.

We accept that accuracy alone is insufficient. In the original validation of the super-unsupervised (SU) measure we used 2,469 expert-coded tweets and obtained 74% agreement against a 50% baseline because the expert labels were balanced. We have now added (i) the baseline accuracy, (ii) precision, recall, and F1, and (iii) the confusion matrix for the held-out test set. We also clarify that the metrics are based on a train/test split of the expert-labelled tweets, which addresses the in-domain/held-out concern.

Furthermore, we now include an interaction model for **share of political tweets** and re-estimate the models on political tweets only to address the concern that high-volume political tweeters are mechanically closer to "political hate." In addition we also include a model where we control for the share of political tweets among all tweets posted by each user. The results from these analyses are presented in the Supporting Information and show that the main results are robust to this additional control; if anything the results are slightly stronger when controlling for the share of political tweets. This suggests that the results are not simply driven by more politically engaged users tweeting more about politics.

We also include correlations between our SU measure and two established measures: such as Perspective API toxicity (r = 0.59) and sentiment (AFINN, r = -0.62, negative because AFINN codes negativity as negative values). This shows that while there is some alignment, our measure captures distinct variance.

We also include brief descriptions of the main other validation results from the original SU paper in the new methods section.

**Manuscript changes:** New validation subsection + extra control in main regression.

### **2. Lexicon measure weak and include correlations with robustness measures**

**Comment:** AFINN is misaligned.

**Response:** We now present AFINN only as a distant robustness check and explicitly note its limitations on social media language. The correlation between AFINN and our embedding measure is r = -0.62 (note: negative because AFINN codes negativity as negative values), showing some alignment but also substantial divergence. Perspective API (r = 0.59) and the supervised classifier are our stronger checks.

**Manuscript changes:** Robustness section reworded.

**Comment:** Include correlations with Perspective API and supervised classifier.

**Response:** We now report correlations with Perspective API, AFINN, and discuss the association with the supervised classifier in the new validation subsection from the original SU paper. In addition we also report a full correlation matrix of key variables in the supplement and reference it in the text.

### **3. Political toxicity**

**Comment:** If the outcome is “political hostility,” restrict toxicity to political tweets.

**Response:** We re-ran toxicity models on tweets classified as political and with a toxicity × political indicator.

**Manuscript changes:** Results → Robustness (with pointer to supplement table).

### **4. Network underspecified**

**Response:** We agree that the network analysis required more specification and careful interpretation than we initially provided. To maintain focus on our core contribution and avoid overstating findings from exploratory network analyses, we have moved this material to S1 Appendix with appropriate methodological details and caveats. The main text now includes only a brief mention of these exploratory results with clear cautionary language about their interpretation.

### **5. Overstated causality**

**Comment:** “Fuels,” “leads to,” “dominates” not warranted.

**Response:** We replaced causal verbs with associative language throughout and clarified that the RI-CLPM helps with temporality and unobserved heterogeneity but does not deliver strong causal identification.

**Manuscript changes:** Abstract, Intro, Discussion.

### **6. Ethics / data-sharing surprise**

**Response:** Addressed under journal requirements: we now provide formal documentation and anonymized materials.

### **7. Minor issues**

**Response:** We fixed PDF artifacts, unified “Twitter (now X)” in a single place and replaced all other occurrences with X, and checked reference formatting.

**Manuscript changes:** Global edits.

## **5. Reviewer #3**

### **1. AFINN is outdated**

**Response:** We agree. We now (i) clearly label AFINN as a secondary/legacy robustness measure, and (ii) report its correlation with our embedding measure to show moderate but imperfect alignment and where it diverges from our expert-labelled hostility measure.

**Manuscript changes:** Robustness section.

### **2. All tweets vs political tweets**

**Response:** We clarified that the main SU score was computed on all tweets but added models on political tweets only and additional robustness checks, such as the Perspective API interaction model and the model controlling for share of political tweets as noted above.

**Manuscript changes:** Methods → Measures; Results → robustness.

### **3. Interpretation of effect sizes**

**Response:** We replaced the 4-SD example with 1.5-SD and softened the “not trivial” wording.

**Manuscript changes:** Section on effect sizes.

### **4. Zero-order correlations**

**Response:** We added a zero-order correlation matrix of key variables to the supplement and reference it in the text.

**Manuscript changes:** Supplement table

### **5. Anonymized scripts**

**Response:** We now provide an anonymized link (OSF) to code and materials, as suggested.

**Manuscript changes:** Data availability.

### **6. Minor presentation issues**

**Response:** We increased marker size in plots, stated explicitly that the data are U.S. data in the Data section, and fixed citation rendering.

**Manuscript changes:** Figures, Data, References.
