# Supplementary material for: Political engagement and online political hostility among American X/Twitter users
Source: PLoS One. 2026 Nov 11;21(11):e0360061. doi: 10.1371/journal.pone.0360061 (PMC13754149; doi:10.1371/journal.pone.0360061)
Supplement: Peer review history

# Peer review: Decision Letter 0

**DOI:** 10.1371/journal.pone.0360061.r001

**Authors:** Vincent Antonio Traag

17 Oct 2025

Dear Dr. Hebbelstrup Rye Rasmussen,

-->First of all, please adhere to PLOS ONE's data sharing policy (https://journals.plos.org/plosone/s/data-availability, in particular "Authors must share the “minimal data set” for their submission") and make your data available for reviewers, rather than upon publication. Similarly, please make all code available for peer review following PLOS ONE's code sharing policy (https://journals.plos.org/plosone/s/materials-software-and-code-sharing). As pointed out by one reviewer, is is also possible to share materials anonymously, for example on OSF directly or through the use of https://anonymous.4open.science/.

Secondly, you indicate that the study does not require an IRB according to Danish law and that the data has been processed in accordance with GDPR regulations according to the University of Aarhus Data Controller Unit. However, GDPR regulations are no substitute for ethical review. Could you please elaborate and explain why no ethical review was required? If possible, please add an exemption from the ethics committee.

Finally, as most reviewers point out, there are substantial questions about the validation and validity of the embedding measure used to operationalise "political hate". Moreover, the network analysis is underdeveloped and requires additional analyses to warrant some of the claims in the manuscript. Finally, as one reviewer points out, it is prudent to reconsider whether causal language is warranted in light of the presented evidence.

Please submit your revised manuscript by Dec 01 2025 11:59PM. If you will need more time than this to complete your revisions, please reply to this message or contact the journal office at ?>plosone@plos.org. When you're ready to submit your revision, log on to https://www.editorialmanager.com/pone/ and select the 'Submissions Needing Revision' folder to locate your manuscript file.

- A rebuttal letter that responds to each point raised by the academic editor and reviewer(s). You should upload this letter as a separate file labeled 'Response to Reviewers'.
- A marked-up copy of your manuscript that highlights changes made to the original version. You should upload this as a separate file labeled 'Revised Manuscript with Track Changes'.
- An unmarked version of your revised paper without tracked changes. You should upload this as a separate file labeled 'Manuscript'.

If you would like to make changes to your financial disclosure, please include your updated statement in your cover letter. Guidelines for resubmitting your figure files are available below the reviewer comments at the end of this letter.

If applicable, we recommend that you deposit your laboratory protocols in protocols.io to enhance the reproducibility of your results. Protocols.io assigns your protocol its own identifier (DOI) so that it can be cited independently in the future. For instructions see: https://journals.plos.org/plosone/s/submission-guidelines#loc-laboratory-protocols. Additionally, PLOS ONE offers an option for publishing peer-reviewed Lab Protocol articles, which describe protocols hosted on protocols.io. Read more information on sharing protocols at https://plos.org/protocols?utm_medium=editorial-email&utm_source=authorletters&utm_campaign=protocols.

We look forward to receiving your revised manuscript.

Kind regards,

Vincent Antonio Traag, Ph.D.

Academic Editor

PLOS ONE

Journal Requirements:

When submitting your revision, we need you to address these additional requirements.

1. Please ensure that your manuscript meets PLOS ONE's style requirements, including those for file naming. The PLOS ONE style templates can be found at

https://journals.plos.org/plosone/s/file?id=wjVg/PLOSOne_formatting_sample_main_body.pdf and

https://journals.plos.org/plosone/s/file?id=ba62/PLOSOne_formatting_sample_title_authors_affiliations.pdf.

2. You indicated that ethical approval was not necessary for your study. We understand that the framework for ethical oversight requirements for studies of this type may differ depending on the setting and we would appreciate some further clarification regarding your research. Could you please provide further details on why your study is exempt from the need for approval and confirmation from your institutional review board or research ethics committee (e.g., in the form of a letter or email correspondence signed by a member of the IRB) that ethics review was not necessary for this study? Please include a copy of the correspondence as an ""Other"" file. Please note that the documentation provided signed by the department head is not sufficient to meet this requirement, and a formal waiver of approval from the IRB is needed.

3. In your Methods section, please include additional information about your dataset and ensure that you have included a statement specifying whether the collection and analysis method complied with the terms and conditions for the source of the data."

4. Please provide additional details regarding participant consent. In the ethics statement in the Methods and online submission information, please ensure that you have specified (1) whether consent was informed and (2) what type you obtained (for instance, written or verbal, and if verbal, how it was documented and witnessed). If your study included minors, state whether you obtained consent from parents or guardians. If the need for consent was waived by the ethics committee, please include this information.

If you are reporting a retrospective study of medical records or archived samples, please ensure that you have discussed whether all data were fully anonymized before you accessed them and/or whether the IRB or ethics committee waived the requirement for informed consent. If patients provided informed written consent to have data from their medical records used in research, please include this information.

5. We note that you have indicated that there are restrictions to data sharing for this study. For studies involving human research participant data or other sensitive data, we encourage authors to share de-identified or anonymized data. However, when data cannot be publicly shared for ethical reasons, we allow authors to make their data sets available upon request. For information on unacceptable data access restrictions, please see http://journals.plos.org/plosone/s/data-availability#loc-unacceptable-data-access-restrictions.

Before we proceed with your manuscript, please address the following prompts:

a) If there are ethical or legal restrictions on sharing a de-identified data set, please explain them in detail (e.g., data contain potentially identifying or sensitive patient information, data are owned by a third-party organization, etc.) and who has imposed them (e.g., a Research Ethics Committee or Institutional Review Board, etc.). Please also provide contact information for a data access committee, ethics committee, or other institutional body to which data requests may be sent.

b) If there are no restrictions, please upload the minimal anonymized data set necessary to replicate your study findings to a stable, public repository and provide us with the relevant URLs, DOIs, or accession numbers. Please see http://www.bmj.com/content/340/bmj.c181.long for guidelines on how to de-identify and prepare clinical data for publication. For a list of recommended repositories, please see https://journals.plos.org/plosone/s/recommended-repositories. You also have the option of uploading the data as Supporting Information files, but we would recommend depositing data directly to a data repository if possible.

Please update your Data Availability statement in the submission form accordingly.

6. Please note that your Data Availability Statement is currently missing [the repository name and/or the DOI/accession number of each dataset OR a direct link to access each database]. If your manuscript is accepted for publication, you will be asked to provide these details on a very short timeline. We therefore suggest that you provide this information now, though we will not hold up the peer review process if you are unable.

7. We note that the grant information you provided in the ‘Funding Information’ and ‘Financial Disclosure’ sections do not match.

When you resubmit, please ensure that you provide the correct grant numbers for the awards you received for your study in the ‘Funding Information’ section.

8. Please update your submission to use the PLOS LaTeX template. The template and more information on our requirements for LaTeX submissions can be found at http://journals.plos.org/plosone/s/latex.

9. Please include captions for your Supporting Information files at the end of your manuscript, and update any in-text citations to match accordingly. Please see our Supporting Information guidelines for more information: http://journals.plos.org/plosone/s/supporting-information.

10. If the reviewer comments include a recommendation to cite specific previously published works, please review and evaluate these publications to determine whether they are relevant and should be cited. There is no requirement to cite these works unless the editor has indicated otherwise.

[Note: HTML markup is below. Please do not edit.]

Reviewers' comments:

Reviewer's Responses to Questions

**Comments to the Author**

1. Is the manuscript technically sound, and do the data support the conclusions?

Reviewer #1: Partly

Reviewer #2: Partly

Reviewer #3: Yes

**********

2. Has the statistical analysis been performed appropriately and rigorously?

Reviewer #1: I Don't Know

Reviewer #2: I Don't Know

Reviewer #3: Yes

**********

3. Have the authors made all data underlying the findings in their manuscript fully available?

The PLOS Data policy

Reviewer #1: No

Reviewer #2: No

Reviewer #3: No

**********

4. Is the manuscript presented in an intelligible fashion and written in standard English?

Reviewer #1: No

Reviewer #2: Yes

Reviewer #3: Yes

**********

Reviewer #1: This manuscript investigates the relationship between political engagement and political hostility on social media platforms, using a US-based twitter dataset + survey data.

The manuscript has an ambitious Scope, the study tackles an important and timely issue which is political hostility online and its "causes", using a combination of self-reported and behavioral data, which is rare to find in the literature.

The linkage of panel survey responses to large-scale Twitter data (over 9 million tweets) is commendable and logistically impressive. The use of Random-Intercept Cross-Lagged Panel Models demonstrates an effort to address concerns of endogeneity and individual heterogeneity. Also, the authors attempt to validate their findings using multiple measures of hostility (word embeddings, sentiment dictionaries, Perspective API) is valuable for robustness of results and generalizability across different proxies of political hostility. Regression analysis is performed with statistical rigour. In general, the manuscript contributes to a growing body of work on the “dark side” of political engagement and affective polarization.

I have, however, some concerns with conceptual vagueness and terminological issues. The term "precursors" appears throughout the manuscript without being explicitly defined. Is it used interchangeably with antecedents or predictors? Its ambiguous use weakens clarity. Similarly, the shift between “political hate,” “political hostility,” and “toxicity” is insufficiently delineated. If the aim is to measure political hostility more broadly than hate speech, the embedding approach centered on a “political hate” vector appears conceptually inconsistent. There is no theoretical or empirical justification for using this specific vector (i.e., “political” + “hate”) as a proxy for political hostility. Affective polarization is treated as a moderator, but the measure is not described in sufficient detail. How is it constructed? Why is it not included in the main text?

Moreover, I have concerns about the methodology. The description of the Super-Unsupervised (SU) embedding-based hostility measure is unclear. It is not clear how the “political hate” vector is constructed, it is not clear how the overall similarity of the tweets to the political hate vector is obtained (summed? averaged? something else?). Moreover, is it a good proxy? How is this methodology based than sentiment analysis or topic modeling or other ML-based methods to label political hostility? The, several methods (e.g., count models, time-series cross-section with pooled OLS, network centrality metrics, and others) are mentioned without explanation or rationale, assuming prior knowledge that is not standard for the generalist audience of PLOS ONE. Finally, the analysis pipeline lacks structure and coherence. The methodological section is disorganized, with unnecessary technicalities (e.g., software packages used for plotting) and insufficient detail on actual statistical models. Moreover, data availability is not confirmed at review time, violating PLOS ONE’s policy, if I interpreted it correctly.

Since my expertise is more on network science than in text mining or inference analysis, I am more confident in saying that the section on online network structure and echo chambers is conceptually underdeveloped and technically weak. I would suggest to either remove it or improve it. Terms like “centrality,” “echo chamber,” and “influence” are used without proper definitions or references and there is a lack of consideration of a vast body of literature on these matters. Stating that some nodes have a lot of connections is not the proper way to assess that these are not in an echo chamber. Visualizations (e.g., Figure 3) are superficial and not analytically informative. No clear metrics (e.g., modularity, assortativity, degree centrality distributions) are presented, which would instead be more informative than the presented figures. The authors may benefit from involving a network science expert to refine both the methodological design and interpretation. The idea of considering the influence of the social network on political hostility is a good idea in my opinion, but the current realization is not sufficiently sound.

I also believe that there is a missed chance to perform a longitudinal network analysis on the evolution of political hostility in relation to partisan interactions: is hostility fuelled by cross-partisan interactions? Are these central users more hostile because they interact with a wider variety of political stances?

There are also some minor issues I would like to mention. The manuscript contains numerous grammatical errors and awkward phrasing, including in the abstract (“fuels hostile” should be “fuels hostility,” “it is political engagement that are driving” → “is driving”). Overly informal language is used in the introduction (e.g., “as any regular user of social media will know”), which is not appropriate for a scientific manuscript. The writing could benefit from editing for conciseness. Redundancy is common, particularly in the background sections. The structure of the manuscript is unclear and deviates from conventional scientific organization. The division of sections is idiosyncratic, and critical methods and results are often buried in appendices. Most relevantly, important results and definitions should not be relegated to the appendices. The main text lacks depth in empirical description.

An additional doubt is: if the authors are affiliated to a Danish university, why performing a US based study? The literature is full of US based analyses especially on the topic of polarization and harmful phenomena on online social media, why not shifting the analysis on a different population?

Recommendation: Major Revision

If the authors are to revise, they should:

1. Clearly define all conceptual constructs, measures, analyses, and models, addressing the concerns stressed above. Justify the use of the word vector "political hate" or change the proxy used to estimate tweets political hostility level.

2. Reorganize the manuscript to follow a standard scientific structure (Introduction, Related Work, Data and Methods, Results, Discussion, Conclusion), or at least improve writing style and structure.

3. Include methodological details and key robustness results in the main text, not just in appendices.

4. Improve the network analysis or remove it and enhance the rest of the contributions

Reviewer #2: This paper links survey measures of political engagement and resources to behavioral indicators of online political hostility among U.S. Twitter/X users. It finds that (i) engagement is positively associated with hostile political content; (ii) this association is stronger among individuals high in affective polarization; and (iii) hostile users have more friends/followers.

Overall: The paper's questions are important, its dataset is impressive, and the findings are useful.

That said, there are major methodological concerns - especially the construct validity of the hostility measure, underspecified network methods, and overly causal language.

### Major issues ###

## Measurement

My main concern is how political hostility is measured.

The main approach uses word2vec embeddings trained on panelists’ tweets and distances to a “political hate” vector (“political” + “hate”). To me, this seems like a highly questionable method. Centrally, it risks capturing whether a user talks about “hate” and “politics”, rather than the _hostile properties_ of the message.

The reported “74 accuracy” against expert labels does not increase confidence in the measure. Accuracy should not be used as a measure, and is in particular not informative without class balance, thresholding, and error decomposition. It is often misleading for imbalanced classes. Please report: baseline accuracy, precision/recall/F1, ROC/AUC, confusion matrix, and whether validation is in-domain or on a held-out set. Also report correlations with AFINN, Perspective API, and the supervised classifier mentioned in the appendix; bring key validation summaries into the main text.

The lexicon-based sentiment measure is not convincing for two reasons: (1) sentiment is conceptually misaligned with hostility; (2) lexicon methods often perform poorly on social media language.

To me, the only measure with some face validity here is Perspective API toxicity - imperfect, but widely used and at least more or less aimed at the target construct.

The problem with just measuring toxicity (rather than _political toxicity_) is that the observed link between “engagement” and “toxicity” in theory might be driven by e.g., sports trash talk. If the research question is _political hostility_, you do need to restrict toxicity scoring to tweets classified as political or interact toxicity with a political-tweet indicator.

If feasible, consider training a modern supervised model (e.g., fine-tuned BERT or a carefully set up zero-shot LLM) and validate it with established protocols and best practices. In all cases, provide full detail on preprocessing, model choices, and validation. And use correct metric for the validation.

On the other hand, because the SU score averages across each user’s tweets, heavy political tweeters may be mechanically closer to “political hate.” So you need to control for share of political tweets (not just volume) and replicate results on matched subsets (e.g., one random political tweet per user per week). The split of “political” vs “hostility” components that's in the appendix is potentially useful here. Maybe move a concise version to the main text to show the political component is not driving it.

## Network analysis

The network analysis also fails to convince. The claim that hostile users “dominate the network” and are not confined to echo chambers is insufficiently substantiated. The manuscript says very little about the network used. How was the network actually made (follower, retweet, mention? snapshot timing? sample boundaries?) What are the risks for interpretation if the graph includes only users in the analytic dataset? There seems to be important issues here.

Moreover, you need to support these claims with proper network measures, not just the rather hard-to-interpret visual that is Figure 3. For instance, assortativity/mixing by hostility, E/I index or modularity for partitions, and comparisons of centrality (degree, eigenvector, betweenness) with uncertainty (e.g., bootstrap or permutation tests). These key numerical evidence should be in the main text, not only in figures. As it stands, Figure 3 is difficult to interpret and may not warrant inclusion without clearer methods and metrics.

## Overstated causality

The manuscript uses causal verbs (“fuels,” “leads to,” “dominates”) that are not supported by the identification strategy. Please tone down causal claims throughout and frame results as associations. Acknowledge alternative mechanisms (topic exposure, elite cues, event-driven spikes) and clarify the limits of the models you employ.

## Ethics and data-sharing

I find the claim that this does not require IRB under Danish law highly surprising. This does not seem compatible with GDPR? I'm moreover not sure if either this nor the data availability statement is compatible with the requirements of the journal, but I leave these questions for the editor.

### Minor issues ###

- Fix text artifacts likely from PDF export (e.g., “a!ective,” “e!cacy”), typos, and double-parenthesis in citaitons

- Is it 'Twitter' or 'X'? I don't really care, but you should stick to one.

- Make sure all references are complete and consistently formatted.

In sum, the study tackles an important problem with valuable data. But there are potentially serious concerns with core aspects of its methodology. I hope to be convinced of the measures' validity by a revised version.

Reviewer #3: This is a very interesting paper that is of broader interest. It is particularly interesting that the authors' take a nuanced stance towards political participation and online hostility, putting forward the argument that despite political participation being normatively desirable, there can be 'too much' of participation.

I have some suggestions that I believe may further strengthen the paper.

- As much as I like the word embedding-based measure the authors use, I (quite strongly) disagree with there alternative measure, the AFINN, and in particular the authors' argument that this is a "standard way". I'd rather say that this is a very outdated approach. In the last years, it has been shown over and over again that sentiment dictionaries do not even agree with each other, let alone that they reliabily measure concepts that they were not initially developed for. Also conceptually, why would the use of so-called 'negative' words even be problematic? We could as well argue that 'positive' words are problematic, as they may signal uncritical adoption. It also seems that there is no validation whatsoever - why not for instance compare the AFINN scores with the expert ratings that are used for the SU-approach?

- Regarding the data, do I understand it correctly that *all* tweets are included, not only political ones? I would assume that this introduces a lot of noise. After all - why would political efficacy influence wether, for instance, someone is more positive or negative about movies/the food they eat/the workings of the railway company etc....?

- I'd like to read more about the interpretation of effect sizes, and at the same time want to warrant about making things bigger than they seem. For instance, in section 7.1, the statement that effects are "not trivial" (p. 13) is corrobated by an example of the effect of a change of 4 (four!) SD's - that's an enormous change of the independent variable that is needed.

- The authors present quite complex models with many variables, and I agree that they make sense. But the authors also allude to potential multicollinearity issues. Given that there are multiple causal directions envisionable, I think the story would be more convincing if the authors also showed more straightforward associations, in particular also zero-order correlations.

Minor comments:

- Rather than specifying that scripts will be made available upon acceptance on OSF, the authors could have opted to include an anonymized link (should be possible via OSF, or via https://anonymous.4open.science/), which would have made it possible for the reviewers to get a clearer picture of what has been done exactly.

- Although it is mentioned in, for instance, the abstract, the section 5.1 Data should mention explicitly the country (US).

- In the plots, I suggest to increase the size of the markers (the triangles, dots, squares)

- The citation commands seem to be partly off and render incorrect brackets etc.

**********

what does this mean?). If published, this will include your full peer review and any attached files.

If you choose “no”, your identity will remain anonymous but your review may still be made public.

**Do you want your identity to be public for this peer review?**  For information about this choice, including consent withdrawal, please see our Privacy Policy

Reviewer #1: **Yes:**  Valentina Pansanella

Reviewer #2: No

Reviewer #3: No

**********

[NOTE: If reviewer comments were submitted as an attachment file, they will be attached to this email and accessible via the submission site. Please log into your account, locate the manuscript record, and check for the action link "View Attachments". If this link does not appear, there are no attachment files.]

While revising your submission, please upload your figure files to the Preflight Analysis and Conversion Engine (PACE) digital diagnostic tool, https://pacev2.apexcovantage.com/. PACE helps ensure that figures meet PLOS requirements. To use PACE, you must first register as a user. Registration is free. Then, login and navigate to the UPLOAD tab, where you will find detailed instructions on how to use the tool. If you encounter any issues or have any questions when using PACE, please email PLOS at figures@plos.org. Please note that Supporting Information files do not need this step.

# Author comment: Author response to Decision Letter 1

**DOI:** 10.1371/journal.pone.0360061.r002

2 Feb 2026

Response to Editor and Reviewers

Manuscript ID: PONE-D-25-18048

Title: “Political Engagement and Online Political Hostility: …”

1. Cover Letter to the Academic Editor

Comment (Editor): Manuscript has merit but needs (a) data & code available for review, (b) clearer ethics/IRB exemption, (c) stronger validation of hostility measure and network analysis, and (d) toned-down causal language.

Response:

We thank the Academic Editor for the constructive decision. In the revision we have:

1. Prepared an anonymized repository with the minimal dataset and analysis code for reviewer inspection. The future link is https://osf.io/82guk/overview and the view-only access can be accessed at https://osf.io/82guk/overview?view_only=68d5ef05464b4ef7ba17b72f968d5879

2. Provided a formal statement from the IRB institution at Aarhus University confirming that the study was not required to undergo full ethical review and, in this case, could not as the IRB institution was only formed after the data collection was initiated. We have expanded the ethics statement accordingly.

3. Reworked the measurement section so that the embedding-based hostility measure is described and validated in the main text, and we added standard metrics (precision, recall, F1, confusion matrix) and comparisons to Perspective API.

4. Narrowed the manuscript scope to focus on the core contribution—the relationship between political engagement and online hostility. Network analyses have been moved to S1 Appendix as exploratory supplementary material with appropriate methodological details and interpretive caveats.

5. Toned down causal language throughout and framed findings as associations.

We address all journal requirements and reviewer comments below.

2. Journal / Administrative Requirements

2.1 PLOS ONE style requirements

Comment: Reformat to PLOS ONE style/templates.

Response: The manuscript has been reformatted according to PLOS ONE guidelines (section order, figure captions, file naming).

Manuscript changes: Updated template and sectioning.

2.2 Ethical approval / exemption

Comment: GDPR ≠ ethics. Provide explanation and formal waiver.

Response: Our data were collected through a U.S. YouGov panel in which participants gave informed digital consent and could voluntarily provide their Twitter handle for linkage to publicly available tweets. Under Danish law and Aarhus University’s procedures, this type of secondary analysis of voluntarily provided survey data linked to public social media data do not require full IRB/REC review, provided that data were processed under the university’s GDPR framework. We have now obtained a formal statement from Aarhus University’s IRB institution confirming that no further approval was required. In this particular case, approval was furthermore not possible to obtain as the institution was only formed after the data collection was initiated.

Manuscript changes: Ethics section expanded to explain (1) source of participants, (2) nature of consent, (3) use of public tweets, (4) institutional waiver.

2.3 Compliance with platform terms

Comment: Add statement on terms/conditions.

Response: We added that Twitter (now X) data were collected only from public accounts and in accordance with the platform’s terms of service at the time of collection.

Manuscript changes: Methods → Data.

2.4 Participant consent details

Comment: Specify informed/written/verbal; minors; waivers.

Response: We now specify that panel participants gave informed digital/written consent through the panel provider; only adults were included; no minors, no waivers.

Manuscript changes: Ethics paragraph in Methods.

2.5 Data sharing / minimal dataset

Comment: Make data available now or explain restrictions and provide contact.

Response: We have uploaded an anonymized minimal dataset (user-wave hostility scores, main covariates) and the analysis code to an anonymized repository. Full tweet-level data cannot be made publicly available because of potential re-identification; access requests can be directed to michael@ps.au.dk.

Manuscript changes: Data Availability statement updated with repository + contact.

2.6 Missing repository / DOI

Response: We added the repository name/DOI in the Data Availability statement.

2.7 Funding mismatch

Response: We reconciled the ‘Funding Information’ and ‘Financial Disclosure’ sections so they list the same grant(s).

Manuscript changes: Funding section updated.

2.8 PLOS LaTeX template

Response: Manuscript converted to the PLOS LaTeX template / reformatted accordingly.

2.9 Captions for Supporting Information

Response: Captions for S1–Sx files added at the end of the manuscript and in-text citations updated.

2.10 Suggested citations

Response: The reviewers did not request that we cite specific previously published works. Reviewer 1 noted a lack of references defining network concepts; however, as we have moved the exploratory network analysis to the Supporting Information (S1 Appendix) to better focus the manuscript, we did not add these definitions to the main text. Regarding Reviewer 3's comment on the limitations of sentiment dictionaries, we have updated the text to explicitly acknowledge these limitations, but no specific papers were requested for citation.

3. Reviewer #1

1. Conceptual vagueness (“precursors”; switching terms)

Comment: Terms “precursors,” “political hate,” “political hostility,” and “toxicity” are used interchangeably.

Response: We now define at the start of the theory section that our target construct is online political hostility (politically grounded degrading/intimidating content). “Political hate” is now used only as the name of the embedding vector in the SU method; “toxicity” is used only for the Perspective API robustness. We also replaced “precursors” with “antecedents/predictors.”

Manuscript changes: Intro + theory + measures clarified.

2. Justification of embedding measure

Comment: No theoretical/empirical justification for "political" + "hate."

Response: We expanded the measurement section to explain the logic of training embeddings on the panelists' own tweets, constructing a composite vector that captures co-occurrence of political and hostile language, and then scoring tweets by cosine similarity. We now report extensive validation results in the main text, including nearest-neighbor analyses showing that the combined vector is surrounded by clearly hostile and political terms, and correlations with expert labels, Perspective API, and a supervised classifier.

Manuscript changes: Methods → Measures (now in main text) + short validation table.

3. SU method unclear

Comment: How is the “overall similarity” obtained?

Response: We now describe the four steps (train → construct vector → compute cosine similarity per tweet → average to user-wave level) and specify that we used averaging.

Manuscript changes: Methods → Measures.

4. Disorganized methods

Comment: Methods contain unnecessary technicalities; important parts in appendices.

Response: We reorganized to a standard structure (Introduction, Related Work, Data, Measures, Methods, Results, Discussion) and moved software details to the supplement; key model info is now in the main text.

Manuscript changes: Section order + pruning.

5. Weak network analysis

Comment: "Centrality," "echo chamber," "influence" used without metrics; Figure 3 not informative.

Response: We appreciate this concern and have reconsidered the scope of our manuscript. Upon reflection, we decided to narrow our focus to the main contribution—the relationship between political engagement and online hostility—and moved the network analyses to S1 Appendix as exploratory supplementary material. In the main text, we now briefly note that exploratory network-level analyses are available in the appendix, with appropriate caveats about their limitations (including that the network is restricted to study participants). We specify the network construction details, report descriptive statistics on tweeting frequency and followers/friends, and tone down claims substantially. This approach allows us to maintain focus on our core theoretical and empirical contribution while making the network material available for interested readers without overstating its robustness.

Manuscript changes: Network section moved to S1 Appendix; brief mention with caveats added to main text Discussion.

6. Language / style / US-only data

Response: We edited for tone and fixed the specific typos the reviewer noted. We added one paragraph in Data explaining that we used a U.S. panel because the theoretical mechanism is most visible in a highly polarized environment.

Manuscript changes: Intro, Data.

4. Reviewer #2

1. Measurement validity (main concern)

Comment: The embedding approach may capture "talking about hate + politics" rather than hostile properties; 74% accuracy is not informative.

Response:

We agree that an embedding approach could in principle pick up meta-talk about ‘hate’. This is why, we in the BJPS paper on the super-unsupervised method, inspected the nearest neighbours of the ‘political’, ‘hate’, and combined ‘political hate’ vectors. In our data, the neighbours are terms such as ‘hateful’, ‘racist’, ‘intolerance’, ‘supremacist’, and ‘partisan’, that is, terms that are both political and clearly hostile, indicating that the model is capturing hostility-in-use rather than mere mentions of the word ‘hate’. We now report this inspection in the main text.

We accept that accuracy alone is insufficient. In the original validation of the super-unsupervised (SU) measure we used 2,469 expert-coded tweets and obtained 74% agreement against a 50% baseline because the expert labels were balanced. We have now added (i) the baseline accuracy, (ii) precision, recall, and F1, and (iii) the confusion matrix for the held-out test set. We also clarify that the metrics are based on a train/test split of the expert-labelled tweets, which addresses the in-domain/held-out concern.

Furthermore, we now include an interaction model for share of political tweets and re-estimate the models on political tweets only to address the concern that high-volume political tweeters are mechanically closer to "political hate." In addition we also include a model where we control for the share of political tweets among all tweets posted by each user. The results from these analyses are presented in the Supporting Information and show that the main results are robust to this additional control; if anything the results are slightly stronger when controlling for the share of political tweets. This suggests that the results are not simply driven by more politically engaged users tweeting more about politics.

We also include correlations between our SU measure and two established measures: such as Perspective API toxicity (r = 0.59) and sentiment (AFINN, r = -0.62, negative because AFINN codes negativity as negative values). This shows that while there is some alignment, our measure captures distinct variance.

We also include brief descriptions of the main other validation results from the original SU paper in the new methods section.

Manuscript changes: New validation subsection + extra control in main regression.

2. Lexicon measure weak and include correlations with robustness measures

Comment: AFINN is misaligned.

Response: We now present AFINN only as a distant robustness check and explicitly note its limitations on social media language. The correlation between AFINN and our embedding measure is r = -0.62 (note: negative because AFINN codes negativity as negative values), showing some alignment but also substantial divergence. Perspective API (r = 0.59) and the supervised classifier are our stronger checks.

Manuscript changes: Robustness section reworded.

Comment: Include correlations with Perspective API and supervised classifier.

Response: We now report correlations with Perspective API, AFINN, and discuss the association with the supervised classifier in the new validation subsection from the original SU paper. In addition we also report a full correlation matrix of key variables in the supplement and reference it in the text.

3. Political toxicity

Comment: If the outcome is “political hostility,” restrict toxicity to political tweets.

Response: We re-ran toxicity models on tweets classified as political and with a toxicity × political indicator.

Manuscript changes: Results → Robustness (with pointer to supplement table).

4. Network underspecified

Response: We agree that the network analysis required more specification and careful interpretation than we initially provided. To maintain focus on our core contribution and avoid overstating findings from exploratory network analyses, we have moved this material to S1 Appendix with appropriate methodological details and caveats. The main text now includes only a brief mention of these exploratory results with clear cautionary language about their interpretation.

5. Overstated causality

Comment: “Fuels,” “leads to,” “dominates” not warranted.

Response: We replaced causal verbs with associative language throughout and clarified that the RI-CLPM helps with temporality and unobserved heterogeneity but does not deliver strong causal identification.

Manuscript changes: Abstract, Intro, Discussion.

6. Ethics / data-sharing surprise

Response: Addressed under journal requirements: we now provide formal documentation and anonymized materials.

7. Minor issues

Response: We fixed PDF artifacts, unified “Twitter (now X)” in a single place and replaced all other occurrences with X, and checked reference formatting.

Manuscript changes: Global edits.

5. Reviewer #3

1. AFINN is outdated

Response: We agree. We now (i) clearly label AFINN as a secondary/legacy robustness measure, and (ii) report its correlation with our embedding measure to show moderate but imperfect alignment and where it diverges from our expert-labelled hostility measure.

Manuscript changes: Robustness section.

2. All tweets vs political tweets

Response: We clarified that the main SU score was computed on all tweets but added models on political tweets only and additional robustness checks, such as the Perspective API interaction model and the model controlling for share of political tweets as noted above.

Manuscript changes: Methods → Measures; Results → robustness.

3. Interpretation of effect sizes

Response: We replaced the 4-SD example with 1.5-SD and softened the “not trivial” wording.

Manuscript changes: Section on effect sizes.

4. Zero-order correlations

Response: We added a zero-order correlation matrix of key variables to the supplement and reference it in the text.

Manuscript changes: Supplement table

5. Anonymized scripts

Response: We now provide an anonymized link (OSF) to code and materials, as suggested.

Manuscript changes: Data availability.

6. Minor presentation issues

Response: We increased marker size in plots, stated explicitly that the data are U.S. data in the Data section, and fixed citation rendering.

Manuscript changes: Figures, Data, References.

Attachment Submitted filename: letter_to_reviewers.docx

# Peer review: Decision Letter 1

**DOI:** 10.1371/journal.pone.0360061.r003

**Authors:** Vincent Antonio Traag

10 May 2026

Dear Dr. Hebbelstrup Rye Rasmussen,

Thank you for submitting your manuscript to PLOS ONE. After careful consideration, we feel that it has merit but does not fully meet PLOS ONE’s publication criteria as it currently stands. Therefore, we invite you to submit a revised version of the manuscript that addresses the points raised during the review process.

The revisions you made to the current manuscript seem to have addressed most of the previous concerns by the reviewers, even if some limitations remain. Unfortunately, some of the reviewers of your original manuscript were no longer available. A new reviewer who kindly stepped in noted a few other weaknesses, mostly related to the theoretical background. In addition, although you already toned down some of the causal wording, some additional causal concerns remain. It should be possible to address these comments without having to present new results, which is why minor revisions of the manuscript are in order.

Please submit your revised manuscript by Jun 24 2026 11:59PM. If you will need more time than this to complete your revisions, please reply to this message or contact the journal office at plosone@plos.org. When you're ready to submit your revision, log on to https://www.editorialmanager.com/pone/ and select the 'Submissions Needing Revision' folder to locate your manuscript file.

- A letter that responds to each point raised by the academic editor and reviewer(s). You should upload this letter as a separate file labeled 'Response to Reviewers'.
- A marked-up copy of your manuscript that highlights changes made to the original version. You should upload this as a separate file labeled 'Revised Manuscript with Track Changes'.
- An unmarked version of your revised paper without tracked changes. You should upload this as a separate file labeled 'Manuscript'.

If you would like to make changes to your financial disclosure, please include your updated statement in your cover letter. Guidelines for resubmitting your figure files are available below the reviewer comments at the end of this letter.

If applicable, we recommend that you deposit your laboratory protocols in protocols.io to enhance the reproducibility of your results. Protocols.io assigns your protocol its own identifier (DOI) so that it can be cited independently in the future. For instructions see: https://journals.plos.org/plosone/s/submission-guidelines#loc-laboratory-protocols. Additionally, PLOS ONE offers an option for publishing peer-reviewed Lab Protocol articles, which describe protocols hosted on protocols.io. Read more information on sharing protocols at https://plos.org/protocols?utm_medium=editorial-email&utm_source=authorletters&utm_campaign=protocols.

As the corresponding author, your ORCID iD is verified in the submission system and will appear in the published article. PLOS supports the use of ORCID, and we encourage all coauthors to register for an ORCID iD and use it as well. Please encourage your coauthors to verify their ORCID iD within the submission system before final acceptance, as unverified ORCID iDs will not appear in the published article. *Only*  the individual author can complete the verification step; PLOS staff *cannot*  verify ORCID iDs on behalf of authors.

We look forward to receiving your revised manuscript.

Kind regards,

Vincent Antonio Traag, Ph.D.

Academic Editor

PLOS One

Journal Requirements:

If the reviewer comments include a recommendation to cite specific previously published works, please review and evaluate these publications to determine whether they are relevant and should be cited. There is no requirement to cite these works unless the editor has indicated otherwise.

Please review your reference list to ensure that it is complete and correct. If you have cited papers that have been retracted, please include the rationale for doing so in the manuscript text, or remove these references and replace them with relevant current references. Any changes to the reference list should be mentioned in the rebuttal letter that accompanies your revised manuscript. If you need to cite a retracted article, indicate the article’s retracted status in the References list and also include a citation and full reference for the retraction notice.

[Note: HTML markup is below. Please do not edit.]

Reviewers' comments:

Reviewer's Responses to Questions

**Comments to the Author**

Reviewer #2: All comments have been addressed

Reviewer #4: (No Response)

**********

2. Is the manuscript technically sound, and do the data support the conclusions?

Reviewer #2: Yes

Reviewer #4: Partly

**********

3. Has the statistical analysis been performed appropriately and rigorously?

Reviewer #2: Yes

Reviewer #4: Yes

**********

4. Have the authors made all data underlying the findings in their manuscript fully available?

The PLOS Data policy

Reviewer #2: Yes

Reviewer #4: (No Response)

**********

5. Is the manuscript presented in an intelligible fashion and written in standard English?

Reviewer #2: Yes

Reviewer #4: Yes

**********

Reviewer #2: The authors have made a commendable effort to address the concerns raised in the first round. The manuscript is clearly improved. They have clarified terminology, reduced overly strong causal claims, expanded the explanation of the SU measure, added standard validation metrics, and included additional robustness checks. They have also narrowed the contribution by moving the weaker network analysis out of the main text and into the appendix, which is a sensible adjustment.

My main concern in the original review was the construct validity of the hostility measure. The revisions improve transparency and strengthen the empirical support, with reported validation metrics and additional checks - they're appropriate and helpful. That said, my underlying reservation is not fully resolved. I still see a risk that the measure captures proximity to political or hate-related language rather than hostility as such. However, I can live with this reservation unresolved.

On the network analysis, I think the authors handled the issue appropriately by reframing it as exploratory and removing it from the core contribution. This effectively removes what was previously a key weakness.

I believe the authors have addressed most concerns sufficiently for publication. The paper is substantially stronger, and the remaining issues are best understood as limitations rather than fatal flaws. On that basis, I'm comfortable recommending publication.

Reviewer #4: The manuscript “Political Engagement and Online Political Hostility: Hostility is Higher Among American Twitter Users with Higher Political Resources, Participation and Engagement” presents an analysis of Twitter data matched to a multi-wave survey that aims to correlate political participation and its antecedents to online hostility. They find an association between the two that is robust to different measurement strategies that is generally stronger among affectively polarized individuals. They further find hostile Twitter users are influential within their networks.

I was not an original reviewer on this manuscript for PLOS One, though I did review it for another journal. Many of the points I raised are still relevant, so I include them below, along with others that have come up with another fresh read of the revised manuscript.

My overall evaluation remains much the same. There are a lot of interesting findings in this manuscript and the data is great. I also share with the authors a desire for more research on what they call the “dark side” of political participation. I think the topic is very important and highly original. But I do have significant concerns with the theorization and structure of the manuscript that would need to be addressed before publication. I have additional points on the strength of claims being made and measurement validity. I note my concerns roughly in order of importance.

• My biggest concern with the manuscript is the muddiness of the theorization. The authors did a good job of highlighting how political engagement can have unintended consequences, like motivated reasoning. But this only applies to some of their variables under the umbrella of “political resources”. I see the case for political interest and knowledge, and education as an antecedent of both based on the classic Lodge and Taber argument, but why would internal efficacy or either form of participation cause hostility? It seems to me participation and hostility may be both caused by political sophistication (interest/knowledge) independently. The authors have a grab bag of indicators of “political resources”, but there needs to be more theory to justify inclusion of some of these.

• This theoretical muddiness also applies to the moderation analysis using affective polarization. Affective polarization can cause political interest and knowledge (or the reverse), which then causes hostility. Or it could cause both of them independently, producing a spurious relationship between resources and hostility. I don’t have a clear sense of the causal model. And the moderating effect of AP is not causally identified in these analyses. I don’t mind a descriptive exercise, but the logic of the moderation needs to at least be fully spelled out and alternative models discussed and addressed. As it stands, I have trouble interpreting the moderation analyses, which really feels tacked on. The theorization of the main effects and the moderator need considerable sharpening, especially when making claims about political engagement having a “dark side”. We more discussion of why that is, and why AP moderates these results.

• The authors also make claims that are perhaps too strong about the moderating role of affective polarization (notwithstanding the theoretical ambiguities I mentioned). Only half of the interaction terms are statistically significant. It is much too strong to claim these expectations are confirmed. There is variation in these effects that deserve some unpacking

• I’m surprised at the measurement approach for affective polarization. Why these, not necessarily representative, party leaders rather than the parties themselves? Or, ideally, focus on feelings towards the supporters of these parties? Ideally the authors can show the robustness of their findings with an alternative, richer measure of affective polarization. This needs more discussion given the robust debate on measurement in affective polarization work

• I’m not clear on why the Big-5 are being used as controls, when the focus of the literature review was on the Dark Triad. Why use Big-5 rather than the Dark Triad, given the previous findings? The authors write about findings about neuroticism and extraversion, but they didn’t really set up these findings by discussing the connection of the Big-5 to online hostility in the main text. What does previous research find in relation to extraversion/neuroticism and online hostility? Ultimately, I’m not sure these results belong in the manuscript absent stronger motivation.

• The authors awkwardly frame their study in juxtaposition to a “resource deficit” approach that lumps in personality explanations (i.e., Dark Triad) with being economically marginalized. I would keep these two types of explanation distinct from one another. They are very different.

• How generalizable is hostility on Twitter to other social media applications. Usage on X tends to be much more overtly political than other applications. Do we expect these relationships to be attenuated for other forms of hostility?

• There are a lot of tests of effectively the same hypothesis. It’s probably appropriate to adjust CIs for multiple comparisons in this case.

• I recommend having clearly delineated hypotheses in the manuscript to aid readability

**********

what does this mean?). If published, this will include your full peer review and any attached files.

If you choose “no”, your identity will remain anonymous but your review may still be made public.

**Do you want your identity to be public for this peer review?**  For information about this choice, including consent withdrawal, please see our Privacy Policy

Reviewer #2: No

Reviewer #4: No

**********

[NOTE: If reviewer comments were submitted as an attachment file, they will be attached to this email and accessible via the submission site. Please log into your account, locate the manuscript record, and check for the action link "View Attachments". If this link does not appear, there are no attachment files.]

To ensure your figures meet our technical requirements, please review our figure guidelines: https://journals.plos.org/plosone/s/figures

You may also use PLOS’s free figure tool, NAAS, to help you prepare publication quality figures: https://journals.plos.org/plosone/s/figures#loc-tools-for-figure-preparation.

NAAS will assess whether your figures meet our technical requirements by comparing each figure against our figure specifications.

# Author comment: Author response to Decision Letter 2

**DOI:** 10.1371/journal.pone.0360061.r004

26 Aug 2026

Response to the Academic Editor

Comment: The revisions addressed most previous concerns, but additional weaknesses remained in the theoretical background and some causal wording still needed to be toned down.

Response: We thank the Academic Editor for this assessment and for the opportunity to revise the manuscript. We have made a set of targeted revisions to sharpen the theoretical framing and further reduce causal language throughout the manuscript. Specifically, we now distinguish between distal antecedents of political engagement, such as education and internal efficacy; motivational and cognitive indicators of engagement, such as political interest and political knowledge; and participatory engagement, such as online and offline participation. We also added a new section on empirical expectations, which states the expectations in explicitly associational terms. In addition, we conducted a manuscript-wide audit of causal language. The title, abstract, introduction, analytical approach, results, RI-CLPM section, discussion, and appendix now consistently describe the findings as observational associations. The RI-CLPM analyses are presented as temporal within-person evidence that reduces concerns about stable time-invariant confounding but does not establish causal effects. We also expanded the discussion of alternative causal models, especially with respect to political sophistication, partisan involvement, and leader-directed affective polarization.

Journal requirements and additional submission items

Comment: Please review the reference list for completeness and correctness, including any retracted articles, and submit the revised manuscript, tracked-changes manuscript, and response letter.

Response: We have reviewed the reference list for completeness and correctness and updated the references where necessary to reflect the revised theoretical discussion and measurement discussion. We have not knowingly cited any retracted articles. The revised submission includes a clean manuscript, a marked-up manuscript with tracked changes, and this point-by-point response letter. The Data Availability statement has also been updated to provide the OSF repository for the anonymized minimal dataset and analysis code. Full post-level X/Twitter data remain restricted because of re-identification risks and X Terms of Service restrictions, as described in the manuscript. No changes were made to the financial disclosure statement.

Comment: If applicable, deposit laboratory protocols in protocols.io and encourage coauthors to verify ORCID iDs.

Response: This study does not involve laboratory protocols, so protocols.io deposition is not applicable. We also appreciate the reminder regarding ORCID verification and will ensure that coauthors verify their ORCID iDs in the submission system where applicable.

Response to Reviewer #2

Comment: All previous comments have been addressed. The manuscript is substantially improved. The remaining reservation concerns the construct validity of the hostility measure: the measure may capture proximity to political or hate-related language rather than hostility as such.

Response: We thank Reviewer #2 for the generous and constructive assessment of the revised manuscript. We are pleased that the reviewer views the manuscript as substantially improved and that the previous revisions addressed the main concerns. We also appreciate the remaining reservation regarding construct validity. In response, we added explicit language acknowledging that no automated text-based measure can perfectly separate hostility from proximity to political conflict language. The revised manuscript now states that the SU measure may partly capture proximity to politically salient conflict language or hate-related political vocabulary rather than hostility alone. At the same time, we retain the measure as our primary operationalization because it is supported by several validation exercises and because the main results are assessed against additional measures of negativity, toxicity, and supervised classification. We have also preserved the cautious framing of the network-related analyses: these analyses remain exploratory and descriptive rather than claims about formal network influence or centrality.

Response to Reviewer #4

We thank Reviewer #4 for the careful and constructive review. We especially appreciate the reviewer’s recognition of the originality and importance of the topic, as well as the concerns about theorization, causal interpretation, and measurement. We agree that the previous version did not sufficiently distinguish among different types of predictors under the broad language of “political resources” and “political engagement.” In response, we have substantially revised the theoretical framing, added clearer empirical expectations, separated distal antecedents from proximate engagement indicators, and made the moderation logic more explicit. We have also further toned down causal language and expanded the discussion of alternative causal models.

1. Theoretical framing of political engagement and its antecedents

Comment: The theorization was muddy. The manuscript treated education, internal efficacy, interest, knowledge, and participation as a grab bag of “political resources.” It was unclear why internal efficacy or participation should cause hostility, and participation and hostility may both be caused by political sophistication.

Response: We agree that the previous version treated conceptually distinct variables too similarly. We have revised the theoretical section to distinguish among three categories. Education and internal efficacy are now described as distal antecedents that may equip citizens with skills, confidence, and perceived competence in politics. Political interest and political knowledge are described as motivational and cognitive indicators of engagement. Online and offline participation are described as participatory engagement, that is, behavioral expressions of engagement. We also clarified that we do not claim that internal efficacy or participation necessarily has a direct causal effect on hostility. Rather, our claim is that politically engaged citizens, and citizens with antecedents that facilitate engagement, may be more likely to enter political conflict on X/Twitter, including antagonistic exchanges. The revised manuscript explicitly discusses the alternative possibility that political sophistication or partisan involvement is a common cause of both participation and hostility. We therefore formulate the expectations in associational terms and avoid treating all indicators as a single undifferentiated resource construct. The supplementary factor analysis is also used to support the decision to treat the indicators separately rather than as one scale.

2. Logic and causal status of affective-polarization moderation

Comment: The moderation analysis using affective polarization was theoretically unclear and not causally identified. Alternative causal models needed to be discussed.

Response: We agree. We added a dedicated theoretical subsection, “Affective polarization as a conditional context.” In the revised manuscript, the logic is that engagement provides attention, motivation, skills, and opportunities to participate in political discussion, while affective polarization may shape whether such participation is expressed in antagonistic terms toward political opponents. We also now explicitly discuss alternative causal models. Affective polarization may increase political interest or participation; political sophistication may increase both affective polarization and hostility; or hostile online interactions may increase affective polarization over time. Because the moderation analyses are observational, we now interpret them as descriptive evidence about conditional associations rather than as causally identified moderation effects.

3. Strength of claims about affective polarization

Comment: The claims about affective polarization were too strong because only some interaction terms were statistically significant.

Response: We agree and have revised the wording throughout the manuscript. The abstract, results, and discussion now state that the moderation evidence is partial and uneven across indicators. The revised results section emphasizes that the conditional pattern is clearest for online and offline participation, while the evidence for more distal antecedents such as education is weaker or inconsistent. We also added multiplicity-adjusted results using Benjamini-Hochberg false-discovery-rate corrections and Bonferroni-adjusted confidence intervals. These analyses reinforce the cautious interpretation: the moderation results are most consistent for participatory engagement and should be treated as exploratory for the remaining indicators. We no longer describe the affective-polarization expectations as uniformly confirmed.

4. Measurement of affective polarization

Comment: The affective-polarization measure uses party leaders rather than parties or party supporters. This choice needed more discussion and, ideally, robustness checks using a richer measure.

Response: We agree that the measure required more precise description and stronger discussion. We now refer to the measure as leader-directed affective polarization. The revised measures section explains that the survey included feeling thermometer ratings for three Republican politicians and three Democratic politicians, which were combined with party identification to distinguish in-party and out-party leaders. The survey did not include parallel thermometer ratings of ordinary party supporters. We therefore could not construct the party-supporter measure suggested by the reviewer. To address this limitation, we added a discussion stating that leader-directed affective polarization may differ from party-directed or supporter-directed affective polarization, and we interpret the moderation analyses accordingly.

5. Big Five controls and Dark Triad accounts

Comment: The Big Five controls were insufficiently motivated, especially because the literature review discussed Dark Triad traits. It was unclear why Big Five traits were included rather than Dark Triad measures.

Response: We have revised this discussion substantially. The Big Five variables are now presented as broad control variables for stable individual differences in motivational, emotional, and cognitive dispositions, not as a direct test of Dark Triad, sadism, or antagonistic-trait accounts. We now explicitly state that the survey did not include Dark Triad or sadism measures and that the analyses therefore cannot directly adjudicate those accounts. We have also reduced the interpretive emphasis on individual Big Five coefficients in the main text and added a limitation noting that future research should examine how political engagement interacts with more specific antagonistic personality constructs.

6. Resource-deficit framing

Comment: The manuscript awkwardly framed the study against a “resource deficit” approach that lumped together personality explanations and economic marginalization. These should be distinct.

Response: We agree and revised the introduction and related-work section accordingly. The revised manuscript now separates socioeconomic and grievance-based explanations from personality and emotion-regulation explanations. We no longer collapse these literatures into a single resource-deficit account. Instead, the manuscript positions our contribution as complementary: online political hostility may be associated not only with deprivation, marginalization, or personality-based explanations, but also with political engagement and its antecedents.

7. Generalizability beyond X/Twitter

Comment: How generalizable is hostility on Twitter/X to other social media applications? X tends to be more overtly political than other platforms.

Response: We agree that the platform context matters. We revised the title, abstract, introduction, results, and discussion to describe the study as an analysis of American X/Twitter users rather than social media users in general. The revised limitations section explicitly states that X/Twitter is a public, text-based, and highly political platform, and that the associations observed here may not generalize directly to platforms with different affordances, audiences, moderation systems, or interaction norms. We now treat cross-platform generalizability as an open empirical question and call for future research on other platforms.

8. Multiple comparisons

Comment: There are many tests of effectively the same hypothesis. Confidence intervals should be adjusted for multiple comparisons.

Response: We agree. We added multiplicity-adjusted results in the S1 Appendix. In the analytical approach section, we now define four families of tests: primary association models, robustness outcome models, affective-polarization interaction models, and exploratory activity/reach models. Within each family we apply Benjamini-Hochberg false-discovery-rate corrections and also report Bonferroni-adjusted confidence intervals for a more conservative reading. The main figures retain conventional 95% confidence intervals for readability, while the appendix reports the adjusted results. These adjustments do not alter the central conclusion that political engagement and its antecedents are associated with online political hostility, but they reinforce the need for caution in interpreting the moderation analyses.

9. Clearly delineated hypotheses or expectations

Comment: The manuscript would benefit from clearly delineated hypotheses to aid readability.

Response: We agree. We added a new section on empirical expectations that states the main expectations in noncausal terms. The expectations now distinguish distal antecedents of engagement, motivational and cognitive indicators of engagement, participatory engagement, and the conditional role of leader-directed affective polarization. We also added an exploratory expectation for the activity and potential-reach analyses and updated the results headings so that the empirical sections correspond more directly to the expectations.

Additional harmonization of manuscript and appendix

In addition to the specific changes above, we harmonized the terminology used in the main manuscript and S1 Appendix. We replaced broad references to “political resources” where that wording could obscure the distinction between distal antecedents, motivational/cognitive engagement, and participatory engagement. We revised causal or causal-sounding language in the RI-CLPM discussion, and we reframed the network/activity analyses as exploratory analyses of activity and potential reach rather than as evidence of formal influence or centrality. These changes were made to ensure that the revised manuscript consistently reflects the observational and descriptive status of the analyses.

Attachment Submitted filename: letter to reviewers MBP[8].docx

# Peer review: Decision Letter 2

**DOI:** 10.1371/journal.pone.0360061.r005

**Authors:** Vincent Antonio Traag

23 Sep 2026

Political Engagement and Online Political Hostility Among American X/Twitter Users

PONE-D-25-18048R2

Dear Dr. Hebbelstrup Rye Rasmussen,

We’re pleased to inform you that your manuscript has been judged scientifically suitable for publication and will be formally accepted for publication once it meets all outstanding technical requirements.

Within one week, you’ll receive an e-mail detailing the required amendments. When these have been addressed, you’ll receive a formal acceptance letter and your manuscript will be scheduled for publication.

An invoice will be generated when your article is formally accepted. Please note, if your institution has a publishing partnership with PLOS and your article meets the relevant criteria, all or part of your publication costs will be covered. Please make sure your user information is up-to-date by logging into Editorial Manager at Editorial Manager® and clicking the ‘Update My Information' link at the top of the page. For questions related to billing, please contact billing support.

If your institution or institutions have a press office, please notify them about your upcoming paper to help maximize its impact. If they’ll be preparing press materials, please inform our press team as soon as possible -- no later than 48 hours after receiving the formal acceptance. Your manuscript will remain under strict press embargo until 2 pm Eastern Time on the date of publication. For more information, please contact onepress@plos.org.

Kind regards,

Vincent Antonio Traag, Ph.D.

Academic Editor

PLOS One

Additional Editor Comments (optional):

Reviewers' comments:

Reviewer's Responses to Questions

**Comments to the Author**

Reviewer #4: All comments have been addressed

**********

2. Is the manuscript technically sound, and do the data support the conclusions?

Reviewer #4: Yes

**********

3. Has the statistical analysis been performed appropriately and rigorously?

Reviewer #4: Yes

**********

4. Have the authors made all data underlying the findings in their manuscript fully available?

The PLOS Data policy

Reviewer #4: Yes

**********

5. Is the manuscript presented in an intelligible fashion and written in standard English?

Reviewer #4: Yes

**********

Reviewer #4: The authors have done an excellent job responding to my concerns. It is theoretically richer than the previous version and I appreciate the additional transparency from the authors on the limitations of design. I recommend publication.

**********

what does this mean?). If published, this will include your full peer review and any attached files.

If you choose “no”, your identity will remain anonymous but your review may still be made public.

**Do you want your identity to be public for this peer review?**  For information about this choice, including consent withdrawal, please see our Privacy Policy

Reviewer #4: No

**********

# Editor report: Acceptance letter

**DOI:** 10.1371/journal.pone.0360061.r006

**Authors:** Vincent Antonio Traag

PONE-D-25-18048R2

PLOS One

Dear Dr. Hebbelstrup Rye Rasmussen,

I'm pleased to inform you that your manuscript has been deemed suitable for publication in PLOS One. Congratulations! Your manuscript is now being handed over to our production team.

At this stage, our production department will prepare your paper for publication. This includes ensuring the following:

* All references, tables, and figures are properly cited

* All relevant supporting information is included in the manuscript submission,

* There are no issues that prevent the paper from being properly typeset

You will receive further instructions from the production team, including instructions on how to review your proof when it is ready. Please keep in mind that we are working through a large volume of accepted articles, so please give us a few days to review your paper and let you know the next and final steps.

Lastly, if your institution or institutions have a press office, please let them know about your upcoming paper now to help maximize its impact. If they'll be preparing press materials, please inform our press team within the next 48 hours. Your manuscript will remain under strict press embargo until 2 pm Eastern Time on the date of publication. For more information, please contact onepress@plos.org.

You will receive an invoice from PLOS for your publication fee after your manuscript has reached the completed accept phase. If you receive an email requesting payment before acceptance or for any other service, this may be a phishing scheme. Learn how to identify phishing emails and protect your accounts at https://explore.plos.org/phishing.

If we can help with anything else, please email us at customercare@plos.org.

Thank you for submitting your work to PLOS One and supporting open access.

Kind regards,

PLOS One Editorial Office Staff

on behalf of

Dr. Vincent Antonio Traag

Academic Editor

PLOS One
